# Supplementary material for: Immune correlates analysis of mRNA-1345 RSV vaccine efficacy clinical trial
Source: Nat Commun. 2025 Jul 3;16:6118. doi: 10.1038/s41467-025-61153-x (PMC12229610; doi:10.1038/s41467-025-61153-x)
Supplement: Supplementary file 1 — Supplementary Information [file 41467_2025_61153_MOESM1_ESM.pdf]

Supplementary Information for

Immune Correlates Analysis of mRNA-1345 RSV Vaccine Efficacy Clinical Trial

Chong Ma et al

Corresponding author: Lingyi Zheng, lingyi.zheng@modernatx.com

The PDF file includes:

Supplementary Methods
Figs. S1 to S39
Tables S1 to S18
Statistical Analysis Plan
Immunogenicity Sampling Plan

Supplementary Methods

Day 29 antibody markers were assessed using the serum samples collected from the participants at their Day 29 visit after receiving the treatment dose, which was presumed to approximate the peak elicited immune response antibody. Theoretically the "Day 29 visit" should be 28 days after the injection of the treatment dose; however, there is a great deal of variability on the date of the "Day 29 visit"; hence, in this immune correlate analysis, a qualified "Day 29 visit" was defined within the window (15, 43) days after the injection of the treatment dose. For ease notation, "Day 29" is exchangeable with the "Day 29 visit"; "Day 29 marker" is exchangeable with the "Day 29 antibody marker"; and "Baseline marker" is exchangeable with the "Baseline antibody marker."

Per-Protocol Efficacy Set for Immune Correlate Analysis (PPESICA)

The study population for the immune correlates analysis consists of all participants in the per-protocol efficacy (PPE) set who had their Day 29 visit during the window (15, 43) days after the injection of mRNA-1345 or placebo. Hereafter, it refers to per-protocol efficacy set for immune correlate analysis (PPESICA) (Table S3).

Case-Cohort Immunogenicity Analysis Set and Day 29 Case-Cohort Set

The case-cohort sampling design according to the mRNA-1345-P301 immunogenicity subset sampling plan was implemented to (1) select a stratified random subcohort (immunogenicity subcohort) from the Full Analysis Set (FAS) (approximately 35,500 participants at the data cut for the first interim analysis (IA1)) based on three baseline characteristics and (2) select all post-injection cases of respiratory syncytial virus-acute respiratory disease (RSV-ARD) cases (between Day 1 up to 24 months after injection). The three baseline characteristics consist of age (60-74 vs. ≥75 years), lower respiratory tract disease (LRTD) risk status (present vs. absent), and region (Northern Hemisphere vs. Southern Hemisphere), respectively. The LRTD risk is defined based on the study participant's relevant past and current medical history, and participants are categorized as LRTD risk present (hereafter, LRTD at-risk) if they have at least one of two risk factors at screening, congestive heart failure or chronic obstructive pulmonary disease (COPD). The Southern Hemisphere includes Argentina, Australia, Chile, New Zealand, and South Africa, and the Northern Hemisphere includes Bangladesh, Belgium, Canada, Colombia, Costa Rica, Finland, Germany, Mexico, Japan, Panama, Poland, Singapore, South Korea, Spain, Taiwan, the United Kingdom and the United States (including Puerto Rico). In the immunogenicity subcohort, participants are selected at random from seven strata by combining each category of three baseline characteristics, except that the participants with age ≥75 years and LRTD risk present are merged by regions. Combining all post-injection RSV-ARD cases, the overall selected participants per the case-cohort sampling design refer to the case-cohort immunogenicity analysis set (CCIAS), and these participants were assessed by the immunologic antibody marker at Day 1 and Day 29.

The Day 29 case-cohort set is a general notation but with slight variability in the correlate analysis for each RSV endpoint (RSV-LRTD-2+, RSV-LRTD-3+, and RSV-ARD) due to the different definitions of endpoints. Almost 100% of the four immunologic antibody marker data were available at Day 1 and Day 29, except a few not applicable or missing values in the four immunologic antibody markers at Day 1 and Day 29.

Baseline Risk Score

For each RSV endpoint (RSV-LRTD-2+, RSV-LRTD-3+, and RSV-ARD), a baseline risk model for predicting the occurrence of RSV endpoint was built by using machine learning on the placebo recipients in the PPESICA. Then the fitted baseline risk model was used to estimate the risk of RSV endpoint for each participant in the PPESICA, which was referred to as the "baseline risk score." A total of 17 prespecified baseline characteristics were used to build the baseline risk model, including World Bank Country Income Level in 2022, geographical region, baseline body mass index group, age, sex, ethnicity, LRTD risk, history of coronavirus disease 2019 (COVID-19), hospitalization due to COVID-19, baseline Edmonton frail scale, preexisting comorbidities of interest, risk factors (COPD, asthma, chronic respiratory disease, diabetes, chronic heart failure, advanced liver or renal disease) (Fig. S37). COPD risk factor was removed before fitting the model due to the high correlation with the LRTD at-risk factor by Spearman correlation > 0.9. Moreover, seven learners were used in machine learning of the placebo recipient data, including linear models (SL.mean, SL.glm, SL.glm.interaction), a nonlinear model (SL.gam), and the ensemble learning models (SL.glmnet, SL.ranger, SL.xgboost), respectively. The three methods, SL.glm, SL.glm.interaction, and SL.gam, used both all baseline covariates and screened covariates (selected by glmnet and univariate correlation methods). The univariate correlation method is to select the top covariates in which the P-value of the Pearson correlation between the covariate and the outcome of RSV endpoint is <0.1. Table S15 showed the weights assigned by the Super Learner algorithm to each individual model for predicting the occurrence of each RSV endpoint, and the specific estimated models for each RSV endpoint were shown in Tables S16 to S18. Figure S38 showed the classification accuracy metric of cross-validated area under the receiver operating characteristic curve (CV-AUC) in placebo and vaccine recipients, respectively. Figure S39 showed high or moderate correlations of estimated baseline risk scores for RSV endpoints, supporting that the goodness of fit by the baseline risk models was satisfied.

Baseline Risk Factors

The actual stratification factors, including age (≥60 and <75 years of age vs. ≥75 years age) LRTD at-risk, and the baseline risk scores, were adjusted in studying the correlate of the antibody marker with each RSV endpoint.

Inverse Probability of Sampling Weight

Inverse probability of sampling weight was calculated for the Day 29 case cohort set per each RSV endpoint based on the two-phase sampling structure. For each RSV endpoint, the "phase 1 population" represents the PPESICA by excluding the participants who had the RSV endpoint onset or censored by 7 days after the Day 29 visit, and the "phase 2 population" represents the Day 29 case-cohort set who had both Day 1 and Day 29 immunogenicity data and had the RSV endpoints onset or censored >7 days after Day 29 visit. Essentially, the "phase 2 population" is a subset of the "phase 1 population." Based on the case-cohort sampling design, the "phase 1 population" can be categorized into 16 strata, including the vaccine case stratum, placebo case stratum, and seven baseline characteristic strata (Figure S3) in the vaccine non-case set and the placebo non-case set, respectively. For each stratum x, n_x represents the stratum size in "phase 2 population," and N_x represents the stratum size in the "phase 1 population", and in each stratum x, the sampling weight for each participant $\pi_i(x)$ was estimated by $\hat{\pi}_i(x) = n_x/N_x$, and the inverse probability of sampling weight $w_i(x)$ was estimated by $\hat{w}_i(x) = 1/\hat{\pi}_i(x)$. Note that for

each RSV endpoint, there was only a slight difference in the corresponding "phase 1 population" and "phase 2 population," and so was the inverse probability of sampling weight.

Correlates of Risk and Correlates of Protection

The univariable inverse probability of sampling weighted Cox proportional hazards regression model was used to conduct the mediation analysis and estimate the cumulative incidence of each RSV endpoint through 345 days after the Day 29 visit (hereafter referred to as study period) per vaccine tertile subgroup, across a range of marker levels during the study period, and above a range of marker levels during study period, respectively.

In addition, the univariate (qualitative) inverse probability of sampling weighted Cox proportional hazards regression model was used to study the correlation of qualitative antibody levels (placebo, low, medium, high vaccine tertiles) with RSV endpoint. Specifically, the vaccine recipients in the Day 29 case-cohort set were categorized into tertiles using the one-third $S(1)_{0.33}$ and two-third quantiles $S(1)_{0.67}$ of the antibody marker levels in the vaccine recipients, and a qualitative antibody marker variable was constructed by ordering the placebo arm, low ($\leq S(1)_{0.33}$), medium $[S(1)_{0.33}, S(1)_{0.67}]$, and high ($\geq S(1)_{0.67}$) vaccine tertiles such that the placebo arm was used as the reference base level. Point estimate with 95% confidence interval for the hazard ratio of RSV endpoint comparing each vaccine tertile to placebo was estimated, and the two-sided P-values (Wald test) and family-wise error rate–adjusted P-values (the Hommel method) were reported.

Cumulative incidence of RSV endpoint during study period

Cumulative incidence of RSV endpoint by a study time of interest t_F after the Day 29 visit at assigned antibody marker levels was estimated for the vaccine and placebo population, respectively, by $\hat{P}(T \leq t_F|S, A) = \frac{\sum_{i=1}^{n_A} w_i \hat{P}(T \leq t_F|S,A,X_i)}{\sum_{i=1}^{n_A} w_i}$, where T is the time-to-event for the RSV endpoint, A is the treatment of receiving vaccine or placebo injections and $A = 1$ for vaccine and $A = 0$ for placebo, S is the antibody marker. t_F is the maximum RSV event time during the study period, and n_A is the number of participants receiving the treatment A in the Day 29 case-cohort set. Specifically, for the ith participant in the treatment group A in the Day 29 case-cohort set, w_i is the corresponding inverse probability of sampling weight and X_i demonstrates the baseline risk factors.

Cumulative incidence of RSV endpoint during study period above marker thresholds

Cumulative incidence of RSV endpoint by a study time of interest t_F after the Day 29 visit above an assigned marker level in vaccine recipients was defined by $P(T \leq t_F|S \geq s_0) = \frac{\int_{s_0}^{\infty} P(T \leq t_F|S=s) f_S(s) ds}{\int_{s_0}^{\infty} f_S(s) ds}$, where $P(T \leq t_F|S \geq s_0)$ was estimated by $\hat{P}(T \leq t_F|S \geq s_0) = \frac{\sum_i w_i \hat{P}(T \leq t_F|S=s_i) I(s_i \geq s_0)}{\sum_i w_i I(s_i \geq s_0)}$, $s_i \sim f_S(s)$, and $\hat{P}(T \leq t_F|S = s_i) = \frac{\sum_j w_j \hat{P}(T \leq t_F|X=x_j, S=s)}{\sum_j w_j}$, $x_j \sim f_X(x)$. Note that f_S represents the distribution of the elicited antibody marker levels by taking vaccine, and f_X represents the distribution of baseline risk factors in the vaccinees, and s_0 is an assigned antibody marker level. And s_i represents a random sample drawing from the distribution $f_S(s)$, and w_i is for the corresponding sampling weight for s_i. Similarly, x_j represents a random sample drawing from the distribution $f_X(x)$, and w_j is for the corresponding sampling weight for x_j.

Mediation analysis

In mediation analysis, the vaccine efficacy mediator was estimated by the ratio of indirect vaccine effect to the overall vaccine effect, i.e., $PM = 1 - \frac{\log(RR_{DE})}{\log(RR)}$, where

$$\underbrace{\frac{E_{S(1)}\left[E[Y(1)=1|S(1)]\right]}{E_{S(0)}\left[E[Y(0)=1|S(0)]\right]}}_{RR} = \underbrace{\sqrt{\frac{E_{S(1)}\left[E[Y(1)=1|S(1)]\right]}{E_{S(0)}\left[E[Y(1)=1|S(0)]\right]}\frac{E_{S(1)}\left[E[Y(0)=1|S(1)]\right]}{E_{S(0)}\left[E[Y(0)=1|S(0)]\right]}}}_{RR_{IDE}} \times$$

$$\underbrace{\sqrt{\frac{E_{S(1)}\left[E[Y(1)=1|S(1)]\right]}{E_{S(1)}\left[E[Y(0)=1|S(1)]\right]}\frac{E_{S(0)}\left[E[Y(1)=1|S(0)]\right]}{E_{S(0)}\left[E[Y(0)=1|S(0)]\right]}}}_{RR_{DE}}$$

RR is the relative risk of RSV endpoint comparing the vaccine population to the placebo population, which can be decomposed into an indirect effect RR_{IDE} and a direct effect RR_{DE}. RR_{IDE} is the geometric mean of the relative risk of RSV endpoint in vaccine population comparing conditional on the actual elicited immune response marker levels by taking vaccine to conditional on the counterfactual elicited immune response marker levels by taking placebo and the relative risk of RSV endpoint in placebo population comparing conditional on the counterfactual elicited immune response marker levels by taking vaccine to conditional on the actual elicited immune response marker levels by taking placebo. And RR_{DE} is the geometric mean of the relative risk of RSV endpoint comparing vaccine population with the actual elicited immune response marker levels by taking vaccine to placebo population with the counterfactual elicited immune response marker levels by taking vaccine and the relative risk of RSV endpoint comparing vaccine population with the counterfactual elicited immune response marker levels by taking placebo to the placebo population with the actual elicited immune response marker levels by taking placebo. $E[Y(a)=1|S(a')] = E_X\left[E[Y(a)=1|S(a'),X]\right] = E_X\left[E[Y=1|A=a,S(a'),X]\right] = E_X[P(T \le t_F|A=a,S(a'),X)]$, represents the marginal probability of getting an RSV endpoint for the population receiving treatment a with the actual or counterfactual elicited immune response marker levels by taking the treatment a'. Note that $Y = I(T \le t_F)$, and $S(a')$ represents the elicited immune response marker levels by taking the treatment a'.

Supplementary Figures

RSV-LRTD-2+ Symptoms		Cases, n/N[a,b] mRNA-1345 N=18,112	Cases, n/N[a,b] Placebo N=18,045	Vaccine Efficacy, % (95% CI)[c]
	Overall	47/18,112	127/18,045	63.3 (48.7, 73.7)
RSV Subtype	RSV-A	24/18,112	78/18,045	69.4 (51.7, 80.6)
	RSV-B	22/18,112	50/18,045	56.3 (27.9, 73.5)
Age Group	60 to 69 years	31/11,219	77/11,170	60.1 (39.5, 73.7)
	70 to 79 years	10/5464	45/5439	78.0 (56.3, 88.9)
	≥80 years	6/1429	5/1436	NE
Frailty Status	Fit (0-3)	37/13,417	104/13,274	65.0 (49.0, 75.9)
	Vulnerable/Frail (≥4)	9/3817	17/3884	46.5 (-20.0, 76.2)
Comorbidities	No comorbidities	31/12,751	76/12,796	59.5 (38.5, 73.4)
	≥1 Cormorbidities[d]	16/5361	51/5249	69.3 (46.1, 82.5)

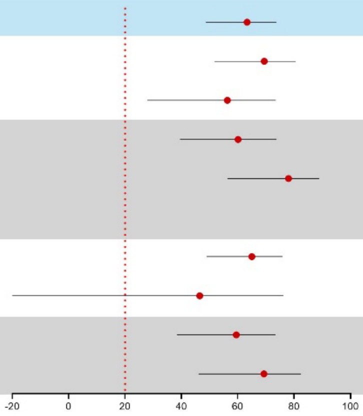

RSV-LRTD-3+ Symptoms		Cases, n/N[a,b] mRNA-1345 N=18,112	Cases, n/N[a,b] Placebo N=18,045	Vaccine Efficacy, % (95% CI)[c]
	Overall	19/18,112	51/18,045	63.0 (37.3, 78.2)
RSV Subtype	RSV-A	10/18,112	30/18,045	66.9 (32.2, 83.8)
	RSV-B	9/18,112	22/18,045	59.3 (11.7, 81.3)
Age Group	60 to 69 years	13/11,219	31/11,170	58.3 (20.3, 78.2)
	70 to 79 years	4/5464	18/5439	77.9 (34.7, 92.5)
	≥80 years	2/1429	2/1436	NE
Frailty Status	Fit (0-3)	13/13,417	41/13,274	68.7 (41.6, 83.2)
	Vulnerable/Frail (≥4)	6/3817	8/3884	NE
Comorbidities	No comorbidities	9/12,751	23/12,796	60.7 (15.2, 81.8)
	≥1 Cormorbidities[d]	10/5361	28/5249	65.0 (28.0, 83.0)

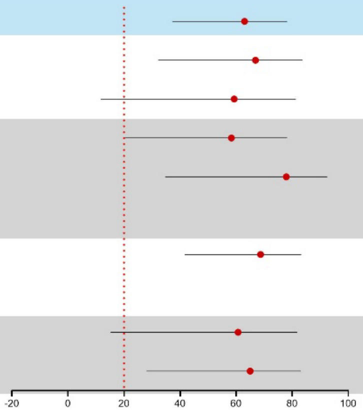

RSV-ARD		Cases, n/N[a,b] mRNA-1345 N=18,112	Cases, n/N[a,b] Placebo N=18,045	Vaccine Efficacy, % (95% CI)[c]
	Overall	86/18,122	185/18,045	53.9 (40.5, 64.3)
RSV Subtype	RSV-A	43/18,112	106/18,045	59.7 (42.5, 71.7)
	RSV-B	42/18,112	80/18,045	47.9 (24.3, 64.1)
Age Group	60 to 69 years	56/11,219	109/11,170	49.1 (29.8, 63.1)
	70 to 79 years	23/5464	69/5439	67.0 (47.0, 79.4)
	≥80 years	7/1429	7/1436	NE
Frailty Status	Fit (0-3)	67/13,417	146/13,274	54.8 (39.6, 66.2)
	Vulnerable/Frail (≥4)	15/3817	27/3884	44.0 (-5.3, 70.2)
Comorbidities	No comorbidities	60/12,751	127/12,796	53.2 (36.4, 65.6)
	≥1 Cormorbidities[d]	26/5361	58/5249	56.1 (30.3, 72.4)

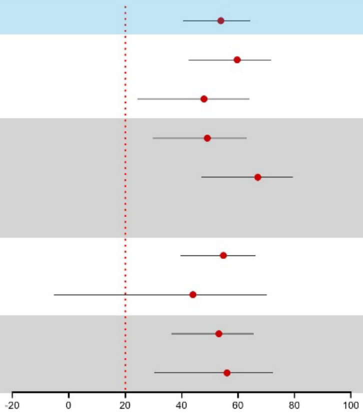

Fig. S1. Vaccine efficacy of mRNA-1345 against RSV-LRTD-2+, RSV-LRTD-3+, and RSV-ARD by RSV subtype and participant sex, age, comorbidity presence, and frailty status among adults ≥60 years (PPE set).

Stratification factors at randomization include age group (60-74 years or ≥75 years) and LRTD risk (present or absent). The red dotted reference line reflects prespecified success criteria in the primary efficacy analysis (November 30, 2022).

ARD, acute respiratory disease; CHD, congenital heart disease; CI, confidence interval; CLIA, Clinical Laboratory Improvement Amendments; COPD, chronic obstructive pulmonary disease; FDA, Food and Drug Administration; LRTD, lower respiratory tract disease; RSV, respiratory syncytial virus; RT-PCR, reverse transcriptase polymerase chain reaction; VE, vaccine efficacy.
[a]RSV-LRTD-2+ symptoms, RSV-LRTD-3+ symptoms, and RSV-ARD were based on eligible symptoms onset within a timeframe of ±14 days from positive RSV RT-PCR collection date. For cases definition, RT-PCR test results from the specialty laboratory were used; if not available, results from a certified laboratory (CLIA or CLIA equivalent) with a regulatory approved (FDA or other agency) RT-PCR test kit were used.
[b]The time to first episode of RSV-LRTD-2+ symptoms, RSV-LRTD-3+ symptoms, and RSV-ARD was calculated as date of case − date of randomization + 1. Participants without a case in the specified time period were censored at the earliest date of 12 months postinjection, date of early discontinuation, date of unrelated death, or data cutoff date. Participants who experienced an early RSV-ARD from Day 1 to Day 14 were censored at the date of the RSV-ARD.
[c]VE was defined as 100% × (1 − hazard ratio [mRNA-1345 vs placebo]). The CI for VE was based on a stratified Cox proportional hazard model, with Efron's method of tie handling and with the vaccination group as a fixed effect, adjusting for stratification factors at randomization.
[d]Comorbidities included in this analysis were chronic cardiopulmonary conditions, including CHF, COPD, asthma, and chronic respiratory conditions, as well as diabetes, advanced liver, and advanced kidney disease.

a

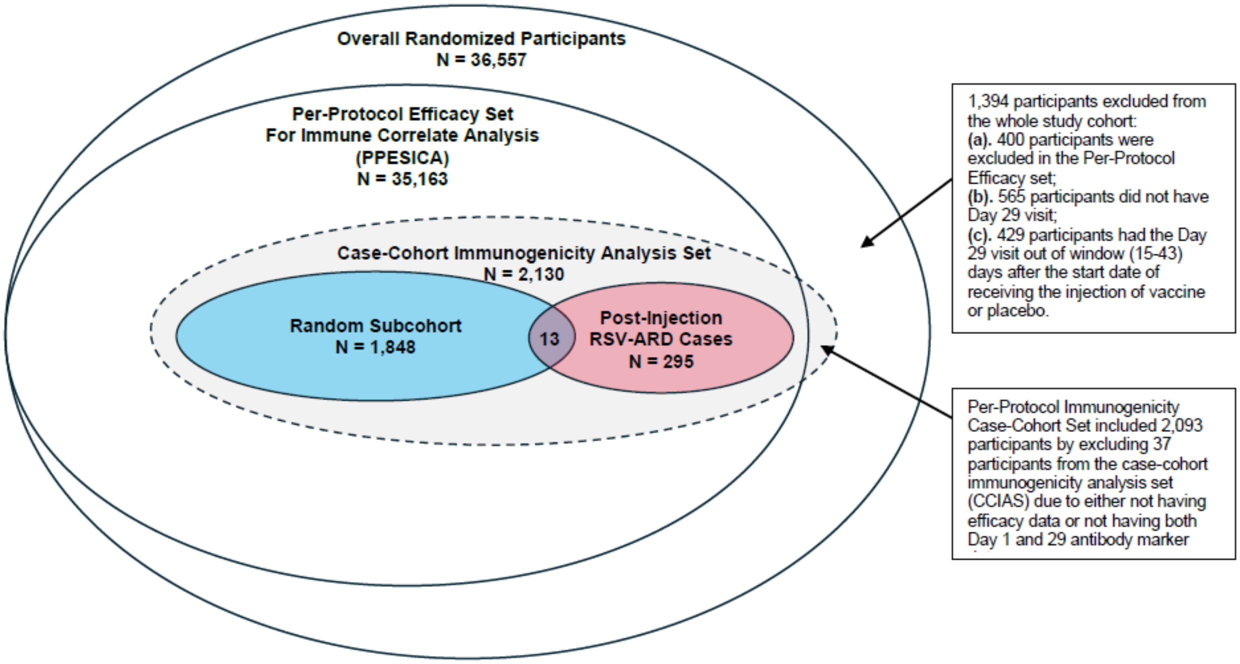
Overall Randomized Participants
N = 36,557
Per-Protocol Efficacy Set
For Immune Correlate Analysis
(PPESICA)
N = 35,163
Case-Cohort Immunogenicity Analysis Set
N = 2,130
Random Subcohort
N = 1,848
13
Post-Injection
RSV-ARD Cases
N = 295
1,394 participants excluded from the whole study cohort:
(a). 400 participants were excluded in the Per-Protocol Efficacy set;
(b). 565 participants did not have Day 29 visit;
(c). 429 participants had the Day 29 visit out of window (15-43) days after the start date of receiving the injection of vaccine or placebo.
Per-Protocol Immunogenicity Case-Cohort Set included 2,093 participants by excluding 37 participants from the case-cohort immunogenicity analysis set (CCIAS) due to either not having efficacy data or not having both Day 1 and 29 antibody marker

b

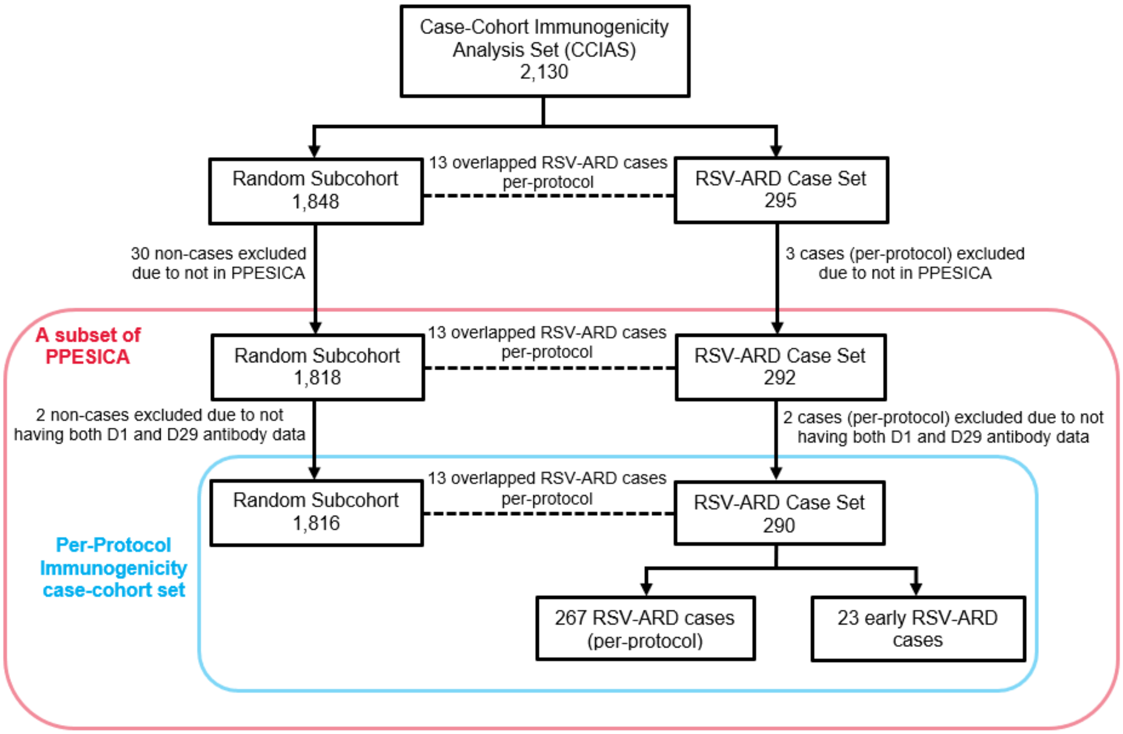
Case-Cohort Immunogenicity Analysis Set (CCIAS) 2,130
Random Subcohort 1,848
13 overlapped RSV-ARD cases per-protocol
RSV-ARD Case Set 295
30 non-cases excluded due to not in PPESICA
3 cases (per-protocol) excluded due to not in PPESICA
A subset of PPESICA
Random Subcohort 1,818
13 overlapped RSV-ARD cases per-protocol
RSV-ARD Case Set 292
2 non-cases excluded due to not having both D1 and D29 antibody data
2 cases (per-protocol) excluded due to not having both D1 and D29 antibody data
Random Subcohort 1,816
13 overlapped RSV-ARD cases per-protocol
RSV-ARD Case Set 290
Per-Protocol Immunogenicity case-cohort set
267 RSV-ARD cases (per-protocol)
23 early RSV-ARD cases

Fig. S2. Case-cohort immunogenicity analysis set Venn diagram.

(a) Most of the CCIAS is overlapped with the eligible study population of the PPESICA. (b) The derivation of the per-protocol immunogenicity case-cohort set is shown, which was used to further derive the Day 29 marker analysis cohort for each RSV endpoint.

ARD, acute respiratory disease; CCIAS, case-cohort immunogenicity analysis set; PPESICA, per-protocol efficacy set for immune correlate analysis; RSV, respiratory syncytial virus.

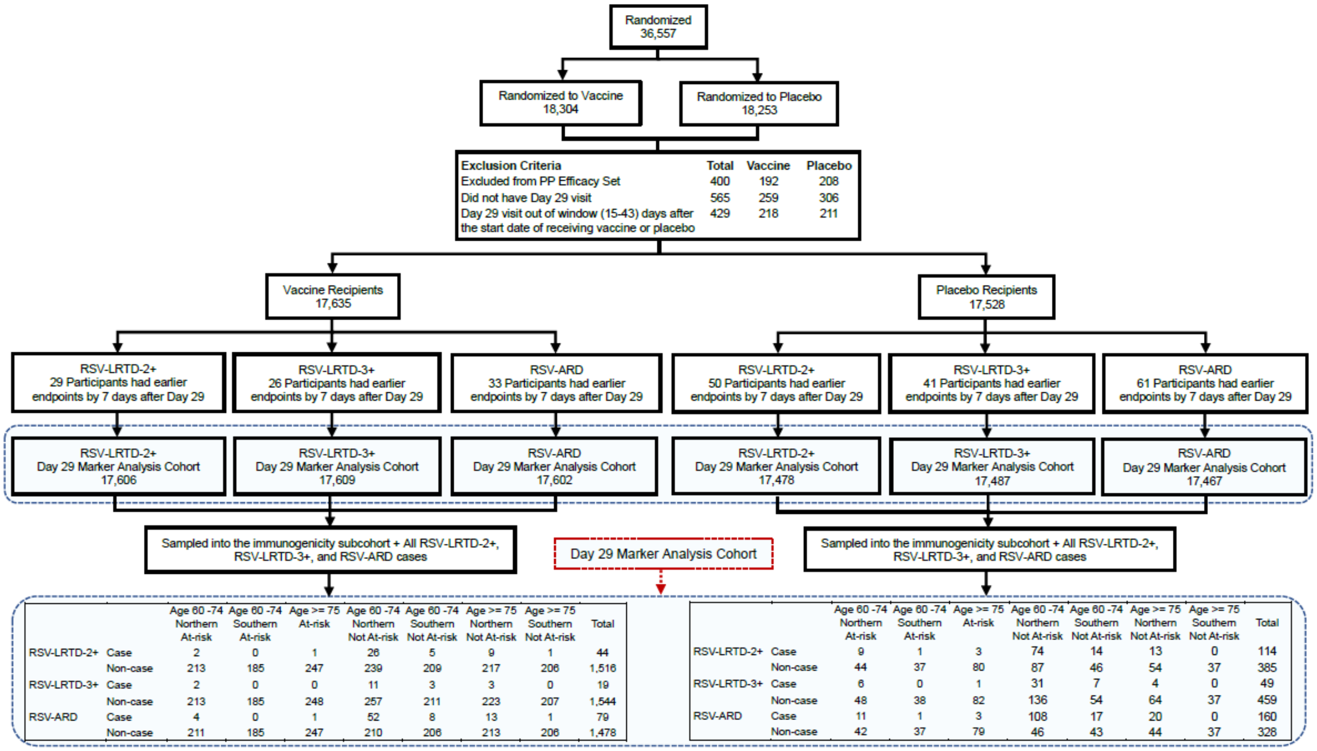

		Age 60 -74 Northern At-risk	Age 60 -74 Southern At-risk	Age >= 75 At-risk	Age 60 -74 Northern Not At-risk	Age 60 -74 Southern Not At-risk	Age >= 75 Northern Not At-risk	Age >= 75 Southern Not At-risk	Total
RSV-LRTD-2+	Case	2	0	1	26	5	9	1	44
	Non-case	213	185	247	239	209	217	206	1,516
RSV-LRTD-3+	Case	2	0	0	11	3	3	0	19
	Non-case	213	185	248	257	211	223	207	1,544
RSV-ARD	Case	4	0	1	52	8	13	1	79
	Non-case	211	185	247	210	206	213	206	1,478

		Age 60 -74 Northern At-risk	Age 60 -74 Southern At-risk	Age >= 75 At-risk	Age 60 -74 Northern Not At-risk	Age 60 -74 Southern Not At-risk	Age >= 75 Northern Not At-risk	Age >= 75 Southern Not At-risk	Total
RSV-LRTD-2+	Case	9	1	3	74	14	13	0	114
	Non-case	44	37	80	87	46	54	37	385
RSV-LRTD-3+	Case	6	0	1	31	7	4	0	49
	Non-case	48	38	82	136	54	64	37	459
RSV-ARD	Case	11	1	3	108	17	20	0	160
	Non-case	42	37	79	46	43	44	37	328

Fig. S3. Flow chart of deriving Day 29 case-cohort sets for each RSV endpoint (ie, RSV-LRTD-2+, RSV-LRTD-3+, and RSV-ARD).

ARD, acute respiratory disease; LRTD, lower respiratory tract disease; PP, per-protocol; RSV, respiratory syncytial virus.

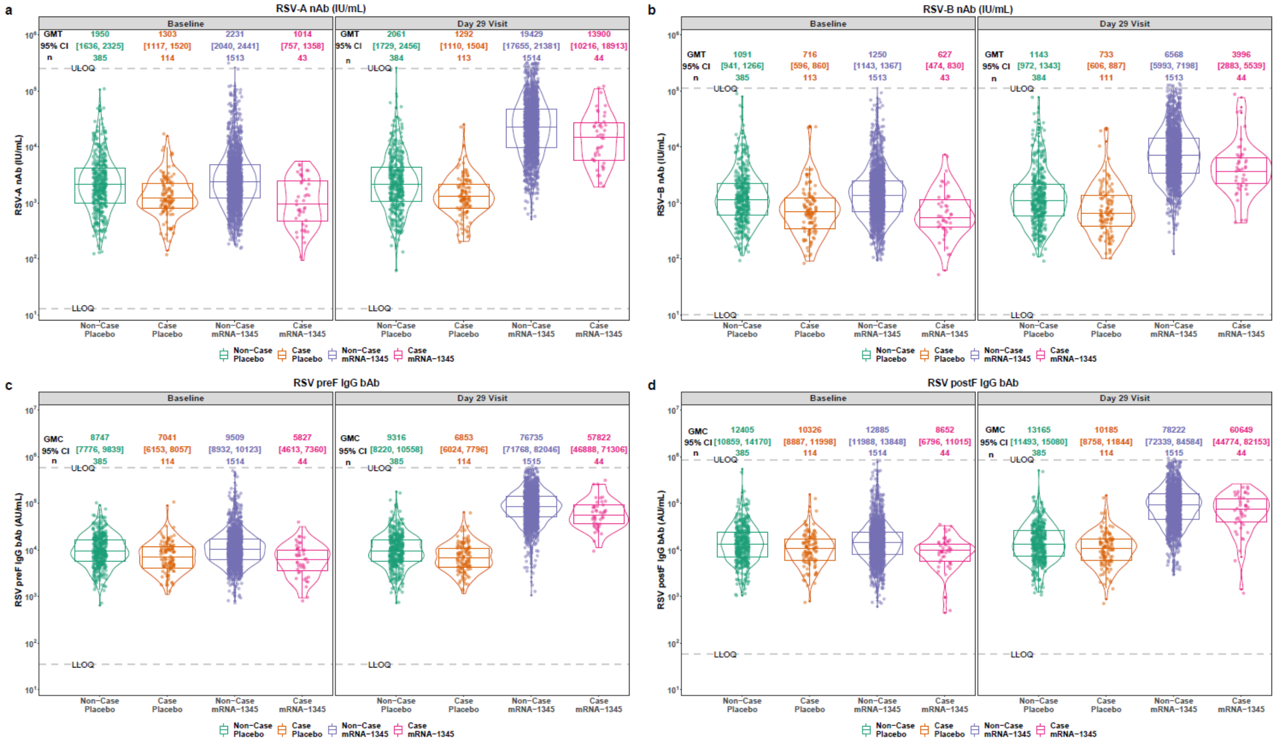

Fig. S4. Baseline and Day 29 RSV nAb (IU/mL) and IgG bAb (AU/mL) by RSV-LRTD-2+ case status and by vaccine and placebo in the Day 29 case-cohort set.

(a) RSV-A nAb. (b) RSV-B nAb. (c) RSV preF IgG bAb. (d) RSV postF IgG bAb. The violin-box plot is composed of an interior box plot and rotated probability density plots (estimated by a default Gaussian kernel density estimator) of the antibody marker data on each side. In the box plot, the middle line and the lower and upper horizonal edges represent the 50th, 25th, and 75th percentile of antibody titers or concentrations, and the vertical whiskers represent the distance from the 25th (or 75th) percentile of antibody titers or concentrations and the minimum (or maximum) antibody titers or concentrations within the 25th (or 75th) percentile of antibody level minus (or plus) 1.5 times the interquartile range. The GMT or GMC level and the corresponding 95% confidence interval were adjusted by the Inverse Probability of Sampling Weight (IPS-Weight).

ARD, acute respiratory disease; bAb, binding antibody; GMC, geometric mean concentration; GMT, geometric mean titer; IgG, immunoglobulin G; LRTD, lower respiratory tract disease; nAb, neutralizing antibody; postF, postfusion; preF, prefusion; RSV, respiratory syncytial virus.

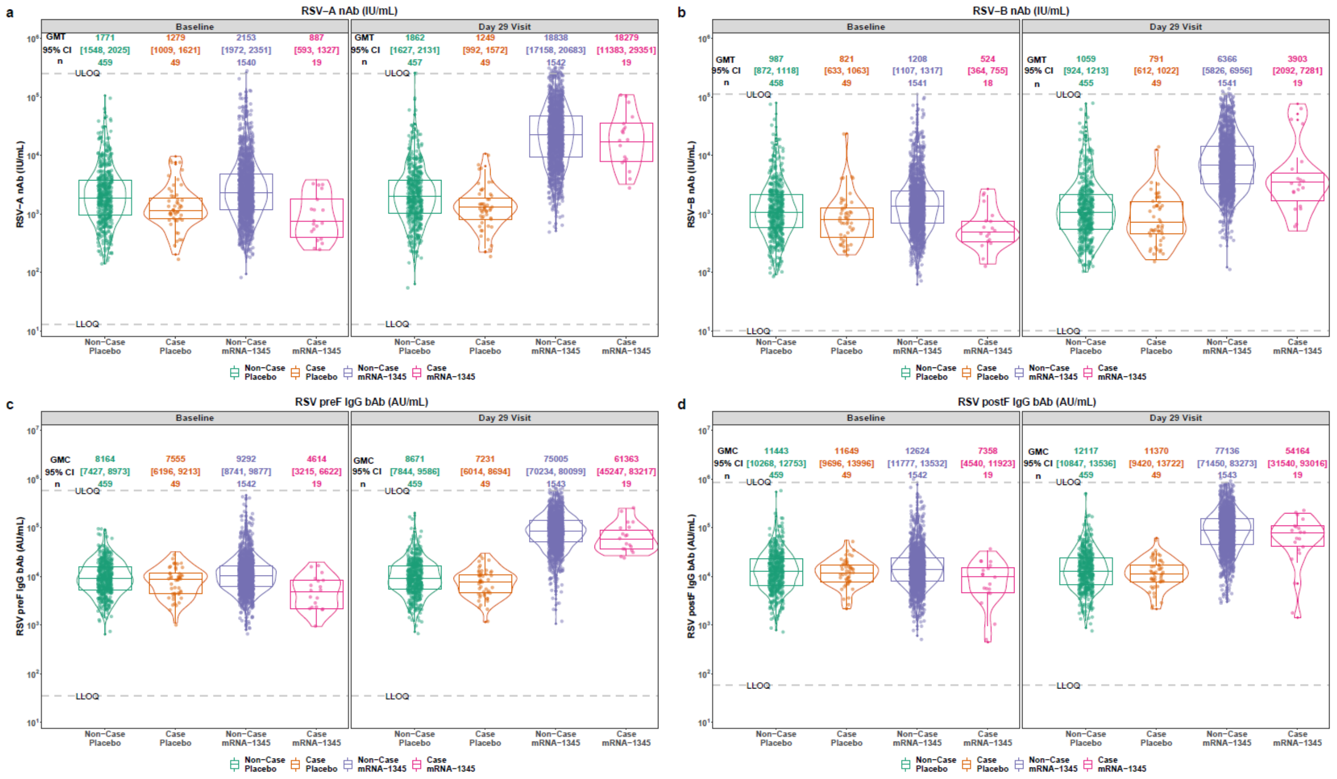

Fig. S5. Baseline and Day 29 RSV nAb (IU/mL) and IgG bAb (AU/mL) by RSV-LRTD-3+ case status and by vaccine and placebo in the Day 29 case-cohort set.

(a) RSV-A nAb. (b) RSV-B nAb. (c) RSV preF IgG bAb. (d) RSV postF IgG bAb. The violin-box plot is composed of an interior box plot and rotated probability density plots (estimated by a default Gaussian kernel density estimator) of the antibody marker data on each side. In the box plot, the middle line and the lower and upper horizonal edges represent the 50th, 25th, and 75th percentiles of antibody titers or concentrations, and the vertical whiskers represent the distance from the 25th (or 75th) percentiles of antibody titers or concentrations and the minimum (or maximum) antibody titers or concentrations within the 25th (or 75th) percentile of antibody level minus (or plus) 1.5 times the interquartile range. The GMT or GMC level and the corresponding 95% confidence interval were adjusted by the Inverse Probability of Sampling Weight (IPS-Weight).

ARD, acute respiratory disease; bAb, binding antibody; GMC, geometric mean concentration; GMT, geometric mean titer; IgG, immunoglobulin G; LRTD, lower respiratory tract disease; nAb, neutralizing antibody; postF, postfusion; preF, prefusion; RSV, respiratory syncytial virus.

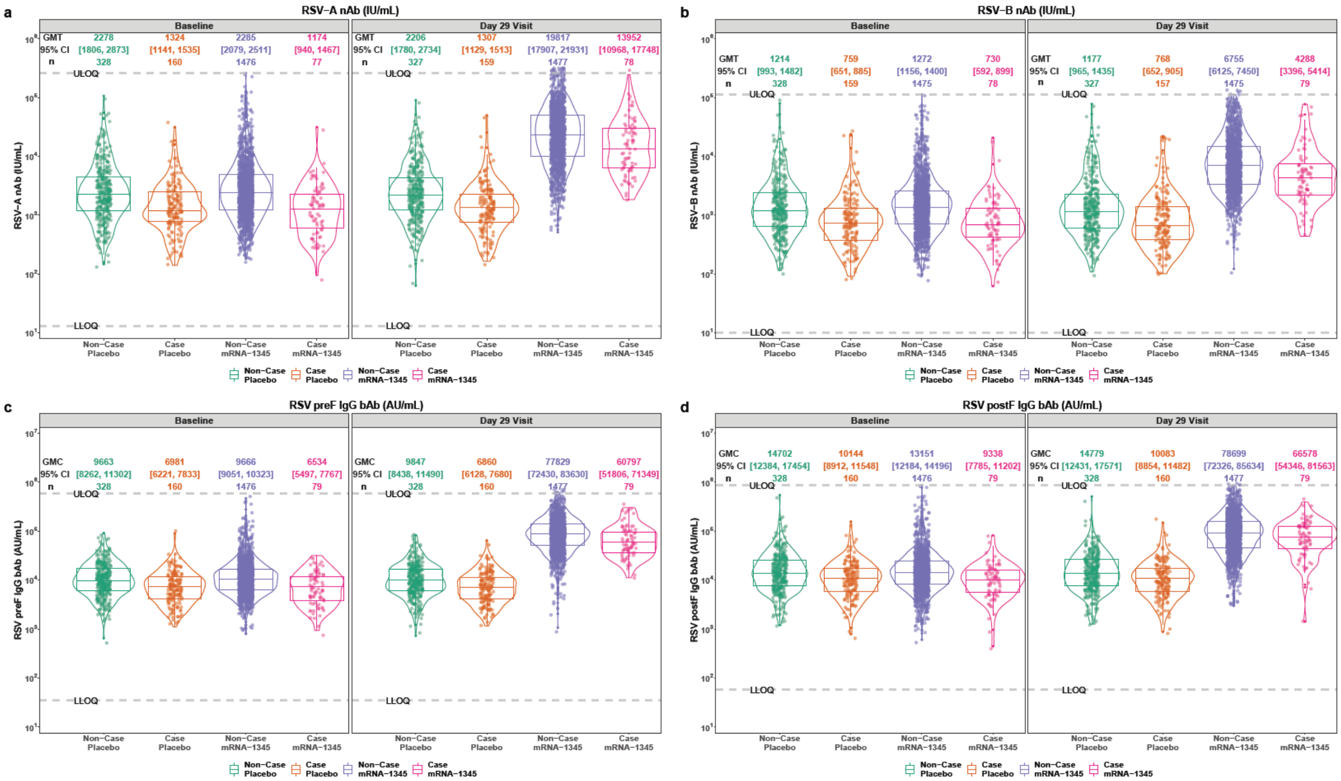

Fig. S6. Baseline and Day 29 RSV nAb (IU/mL) and IgG bAb (AU/mL) by RSV-ARD case status and by vaccine and placebo in the Day 29 case-cohort set.

(a) RSV-A nAb. (b) RSV-B nAb. (c) RSV preF IgG bAb. (d) RSV postF IgG bAb. The violin-box plot is composed of an interior box plot and rotated probability density plots (estimated by a default Gaussian kernel density estimator) of the antibody marker data on each side. In the box plot, the middle line and the lower and upper horizonal edges represent the 50th, 25th, and 75th percentile of antibody titers or concentrations, and the vertical whiskers represent the distance from the 25th (or 75th) percentile of antibody titers or concentrations and the minimum (or maximum) antibody titers or concentrations within the 25th (or 75th) percentile of antibody level minus (or plus) 1.5 times the interquartile range. The GMT or GMC level and the corresponding 95% confidence interval were adjusted by the Inverse Probability of Sampling Weight (IPS-Weight).

ARD, acute respiratory disease; bAb, binding antibody; GMC, geometric mean concentration; GMT, geometric mean titer; IgG, immunoglobulin G; LRTD, lower respiratory tract disease; nAb, neutralizing antibody; postF, postfusion; preF, prefusion; RSV, respiratory syncytial virus.

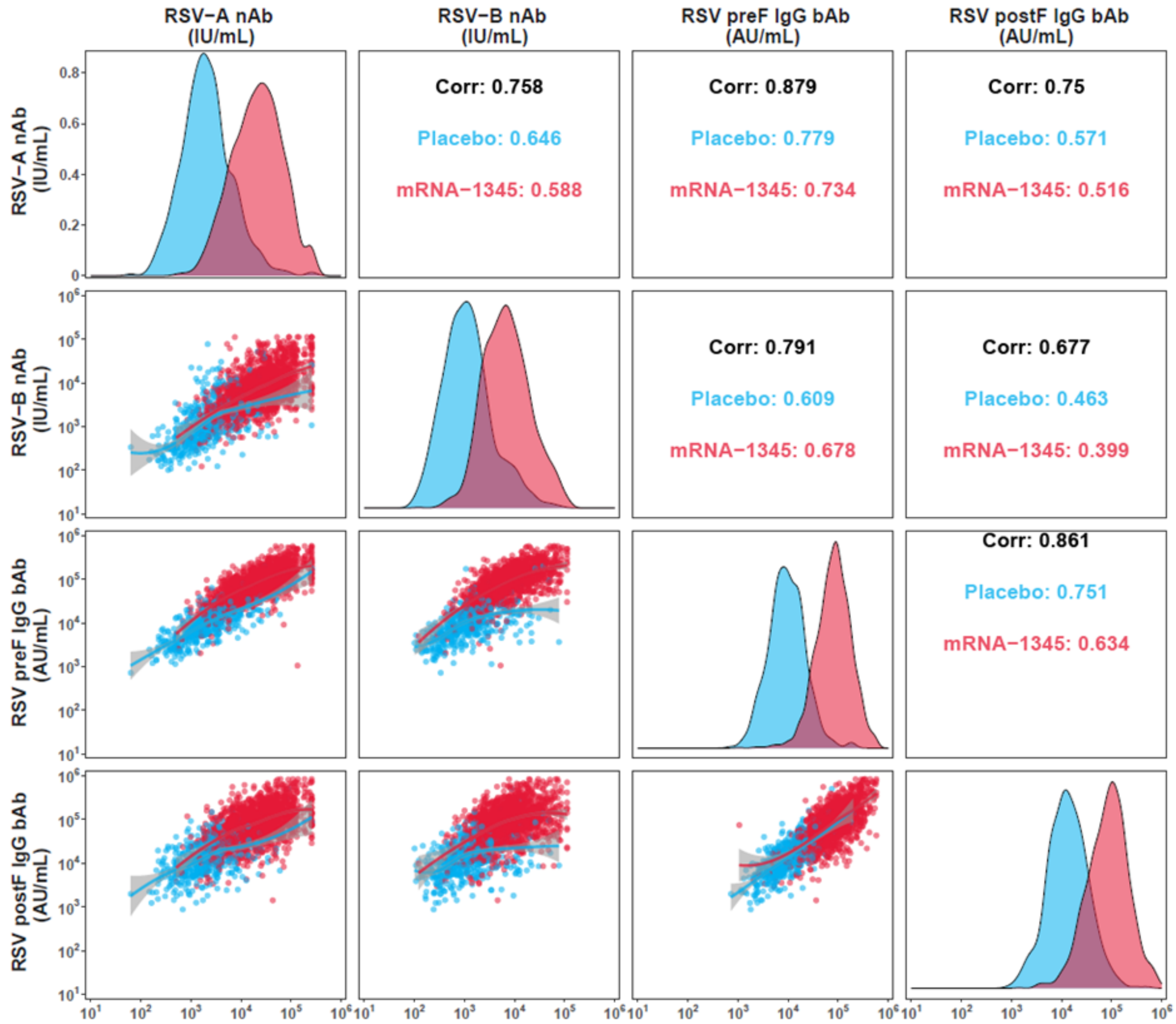

Fig. S7. Correlations of Day 29 antibody markers in both vaccine and placebo recipients in the Day 29 case-cohort set for RSV-LRTD-2+.

The inverse probability of sampling weight adjusted Spearman rank correlations between each pair of antibody markers in vaccine recipients (red), placebo recipients (blue), and the combined placebo and vaccine recipients (black), respectively.

ARD, acute respiratory disease; bAb, binding antibody; Corr, correlation; GMC, geometric mean concentration; GMT, geometric mean titer; IgG, immunoglobulin G; LRTD, lower respiratory tract disease; nAb, neutralizing antibody; postF, postfusion; preF, prefusion; RSV, respiratory syncytial virus.

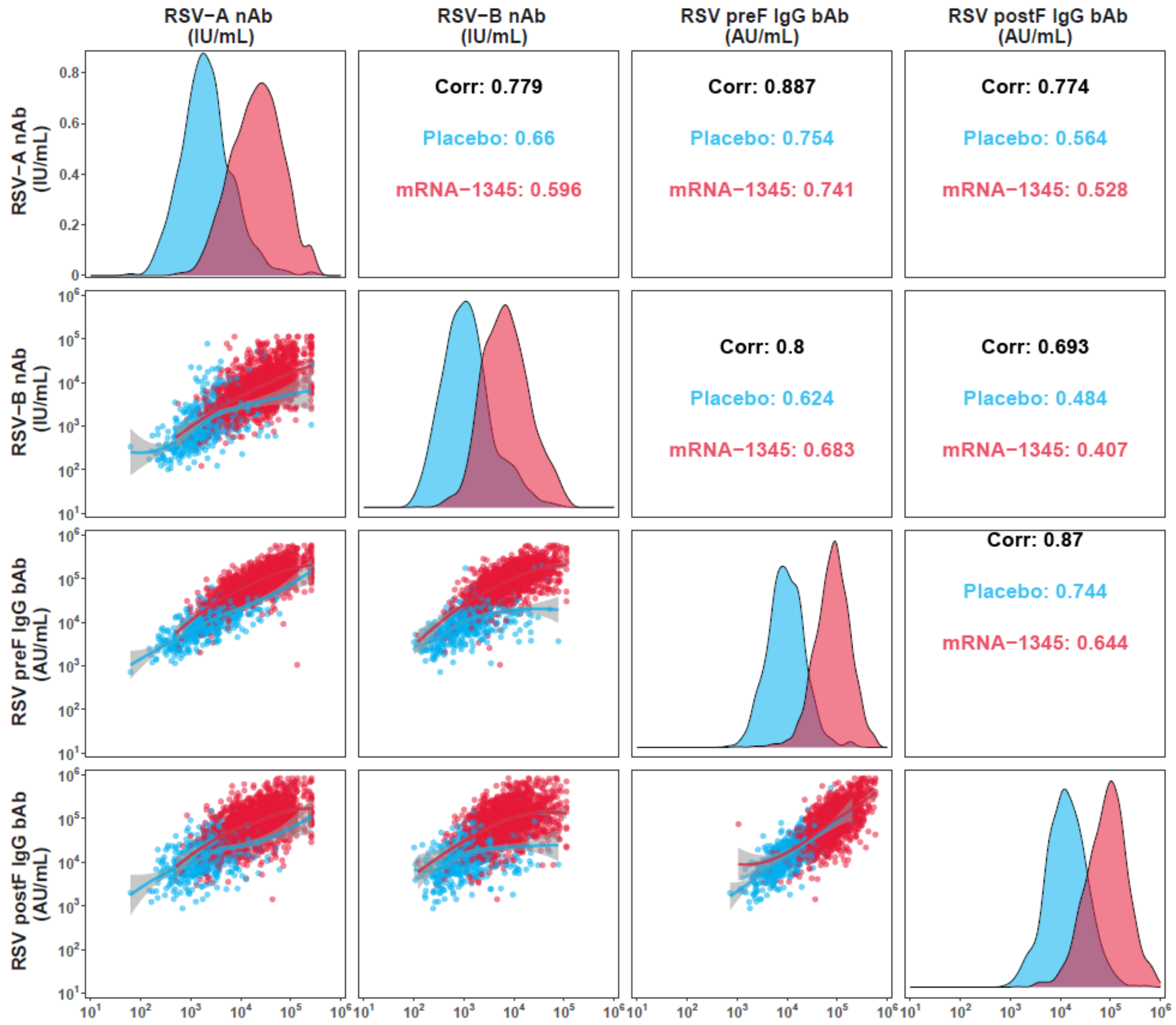

Fig. S8. Correlations of Day 29 antibody markers in both vaccine and placebo recipients in the Day 29 case-cohort set for RSV-LRTD-3+.

The inverse probability of sampling weight adjusted Spearman rank correlations between each pair of antibody markers in vaccine recipients (red), placebo recipients (blue), and the combined placebo and vaccine recipients (black), respectively.

ARD, acute respiratory disease; bAb, binding antibody; Corr, correlation; GMC, geometric mean concentration; GMT, geometric mean titer; IgG, immunoglobulin G; LRTD, lower respiratory tract disease; nAb, neutralizing antibody; postF, postfusion; preF, prefusion; RSV, respiratory syncytial virus.

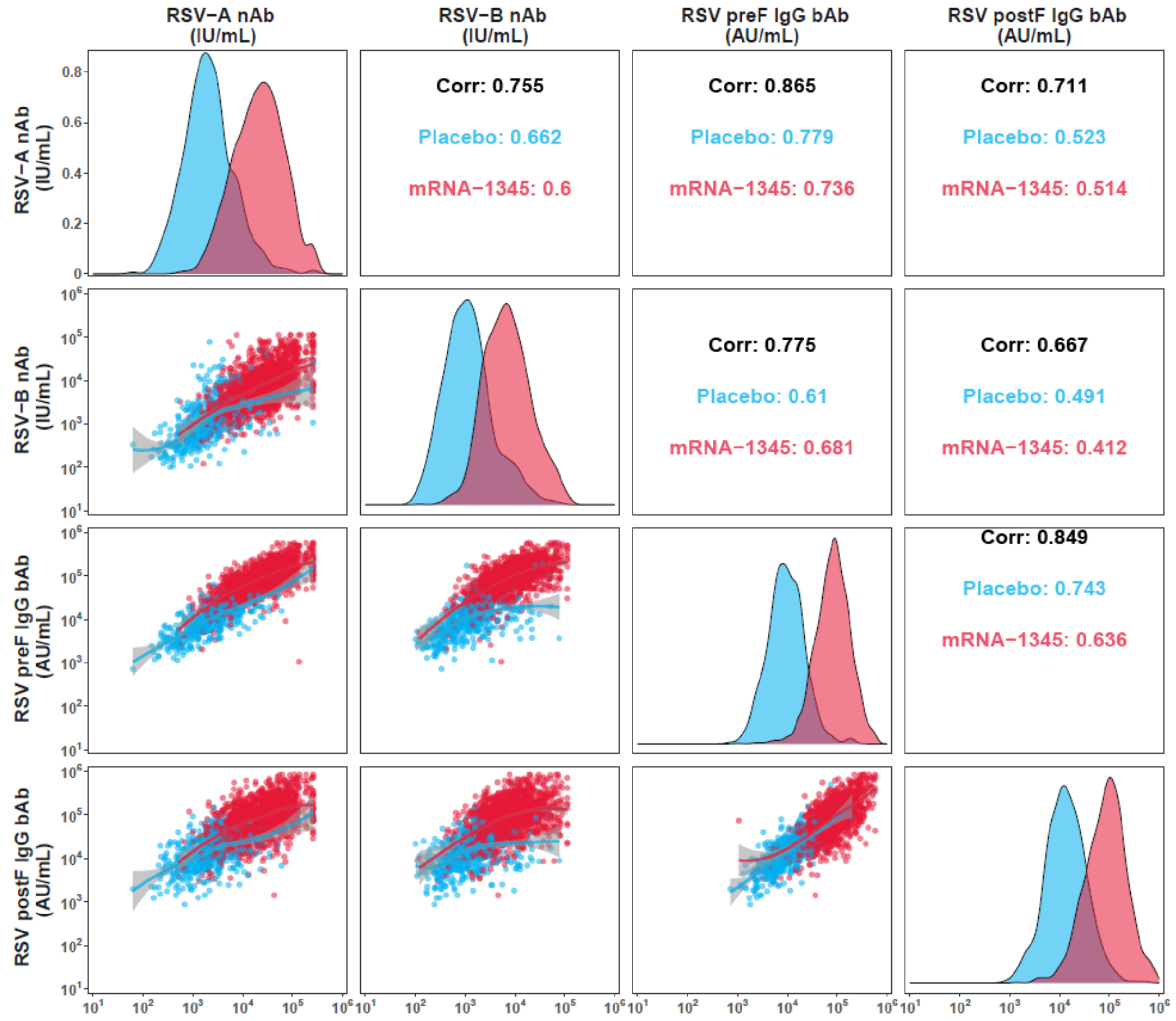

Fig. S9. Correlations of Day 29 antibody markers in both vaccine and placebo recipients in the Day 29 case-cohort set for RSV-ARD.

The inverse probability of sampling weight adjusted Spearman rank correlations between each pair of antibody markers in vaccine recipients (red), placebo recipients (blue), and the combined placebo and vaccine recipients (black), respectively.

ARD, acute respiratory disease; bAb, binding antibody; Corr, correlation; GMC, geometric mean concentration; GMT, geometric mean titer; IgG, immunoglobulin G; LRTD, lower respiratory tract disease; nAb, neutralizing antibody; postF, postfusion; preF, prefusion; RSV, respiratory syncytial virus.

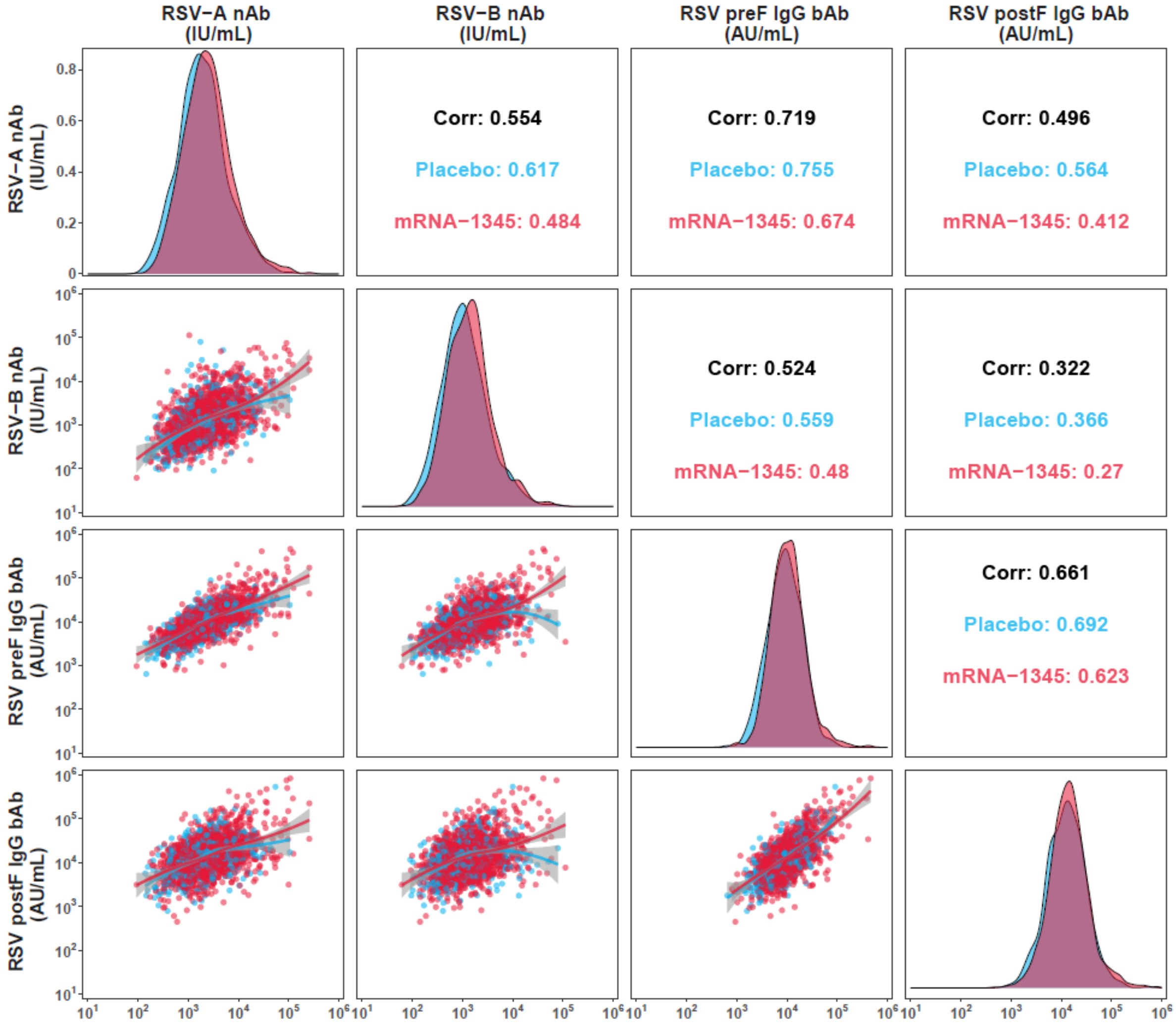

Fig. S10. Correlations of baseline antibody markers in both vaccine and placebo recipients in the Day 29 case-cohort set for RSV-LRTD-2+.

The inverse probability of sampling weight adjusted Spearman rank correlations between each pair of antibody markers in vaccine recipients (red), placebo recipients (blue), and the combined placebo and vaccine recipients (black), respectively.

ARD, acute respiratory disease; bAb, binding antibody; Corr, correlation; GMC, geometric mean concentration; GMT, geometric mean titer; IgG, immunoglobulin G; LRTD, lower respiratory tract disease; nAb, neutralizing antibody; postF, postfusion; preF, prefusion; RSV, respiratory syncytial virus.

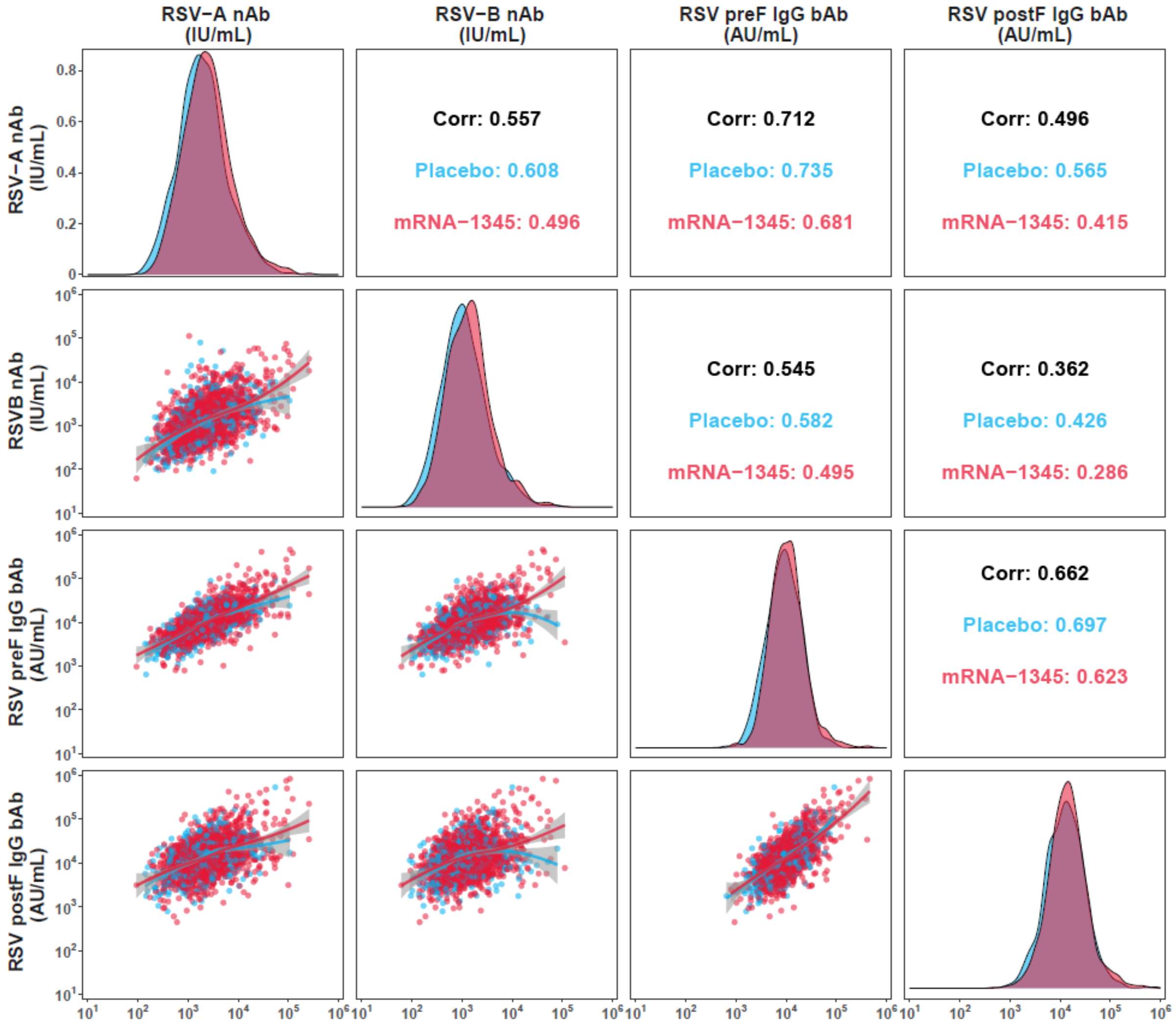

Fig. S11. Correlations of baseline antibody markers in both vaccine and placebo recipients in the Day 29 case-cohort set for RSV-LRTD-3+.

The inverse probability of sampling weight adjusted Spearman rank correlations between each pair of antibody markers in vaccine recipients (red), placebo recipients (blue), and the combined placebo and vaccine recipients (black), respectively.

ARD, acute respiratory disease; bAb, binding antibody; Corr, correlation; GMC, geometric mean concentration; GMT, geometric mean titer; IgG, immunoglobulin G; LRTD, lower respiratory tract disease; nAb, neutralizing antibody; postF, postfusion; preF, prefusion; RSV, respiratory syncytial virus.

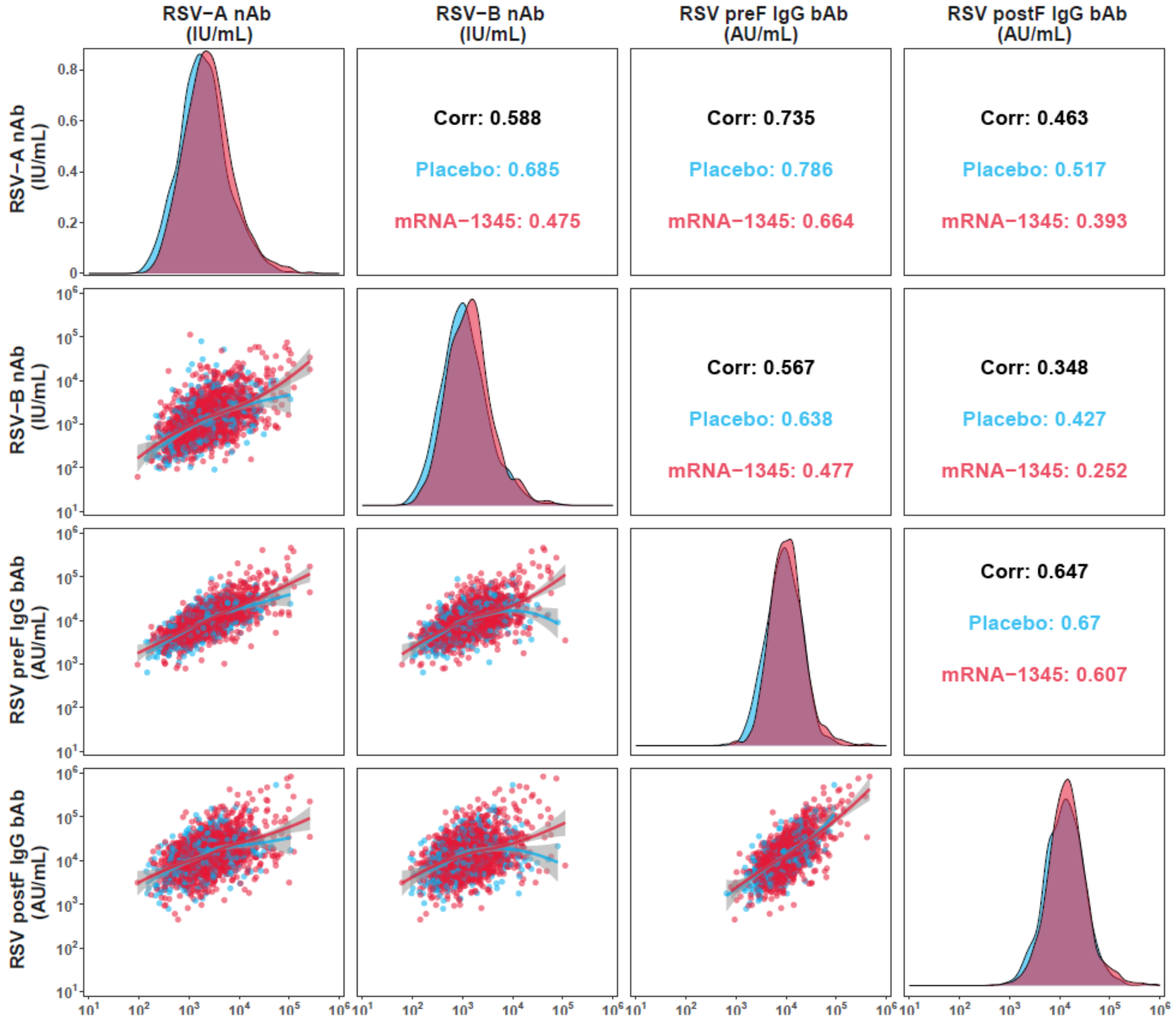

Fig. S12. Correlations of baseline antibody markers in both vaccine and placebo recipients in the Day 29 case-cohort set for RSV-ARD.

The inverse probability of sampling weight adjusted Spearman rank correlations between each pair of antibody markers in vaccine recipients (red), placebo recipients (blue), and the combined placebo and vaccine recipients (black), respectively.

ARD, acute respiratory disease; bAb, binding antibody; Corr, correlation; GMC, geometric mean concentration; GMT, geometric mean titer; IgG, immunoglobulin G; LRTD, lower respiratory tract disease; nAb, neutralizing antibody; postF, postfusion; preF, prefusion; RSV, respiratory syncytial virus.

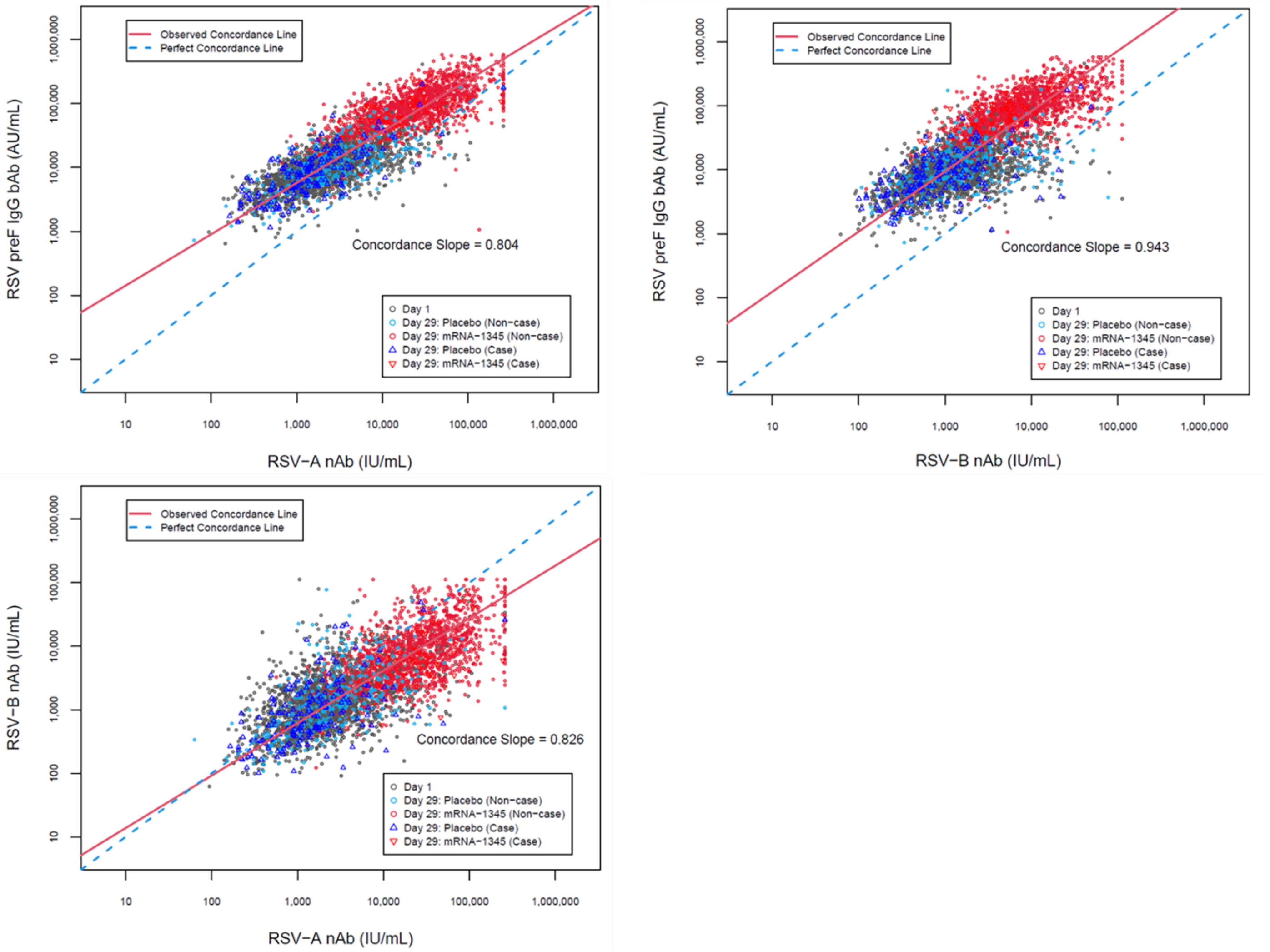

Fig. S13. Concordance analysis between RSV-A nAb, RSV-B nAb, and RSV preF IgG bAb assessed by the slope and PCC.

The slope and PCC in concordance of RSV-A nAb and RSV preF IgG bAb are 0.80 (90% CI, 0.79-0.82) and 0.8650. The slope and PCC in concordance of RSV-B nAb and RSV preF IgG bAb are 0.94 (90% CI, 0.92-0.96) and 0.7790. The slope and PCC in concordance of RSV-A nAb and RSV-B nAb are 0.83 (90% CI, 0.81-0.85) and 0.7496.

bAb, binding antibody; IgG, immunoglobulin G; nAb, neutralizing antibody; PCC, Pearson correlation coefficient; preF, prefusion; RSV, respiratory syncytial virus.

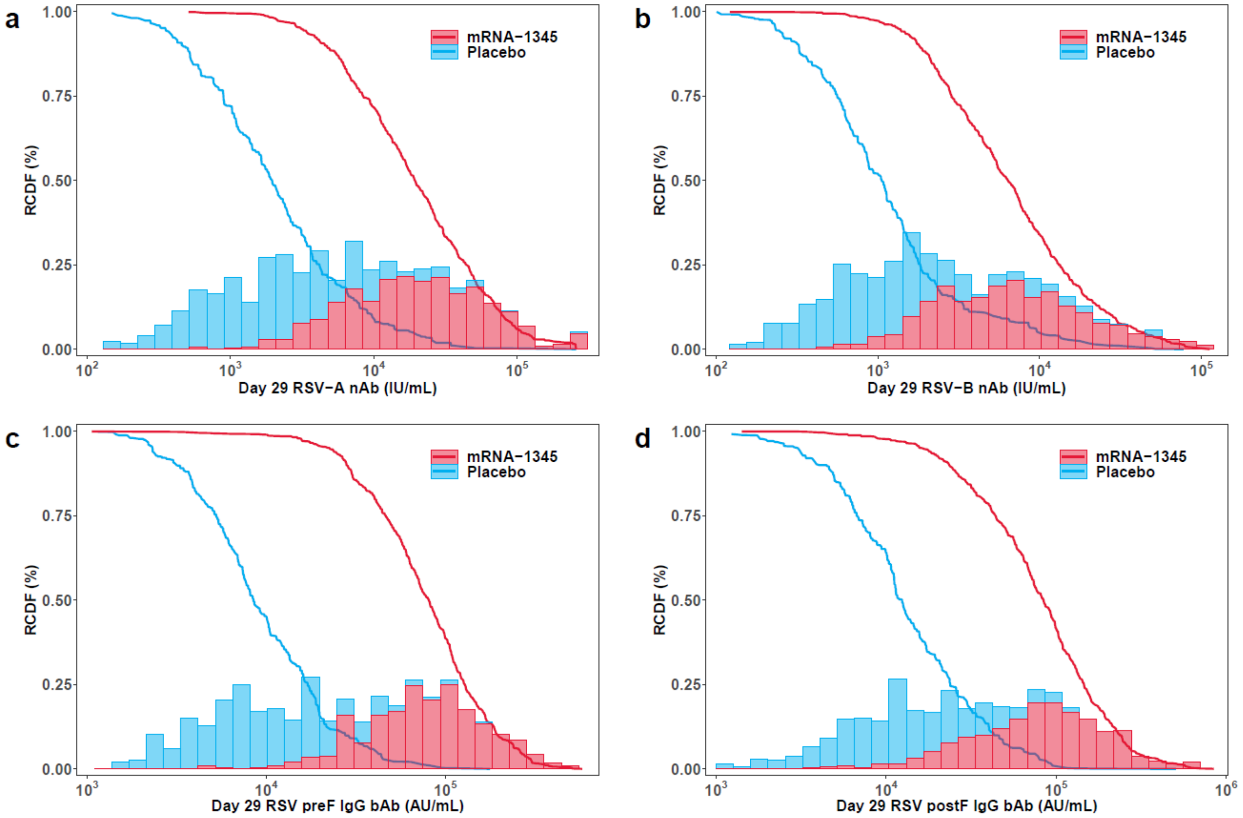

Fig. S14. Inverse probability of sampling weight adjusted reverse cumulative distribution function curves for each Day 29 antibody marker (RSV-A nAb [a], RSV-B nAb [b], RSV preF IgG bAb [c], and RSV postF IgG bAb [d]) in vaccine and placebo recipients in the Day 29 case-cohort set for RSV-LRTD-2+.

Inverse probability of sampling weight was calculated with respect to RSV-LRTD-2+. The stacked histograms display the inverse probability of sampling weight adjusted distribution of Day 29 antibody markers of vaccine (red) and placebo (blue).

bAb, binding antibody; IgG, immunoglobulin G; LRTD, lower respiratory tract disease; nAb, neutralizing antibody; postF, postfusion; preF, prefusion; RCDF, reverse cumulative distribution function; RSV, respiratory syncytial virus.

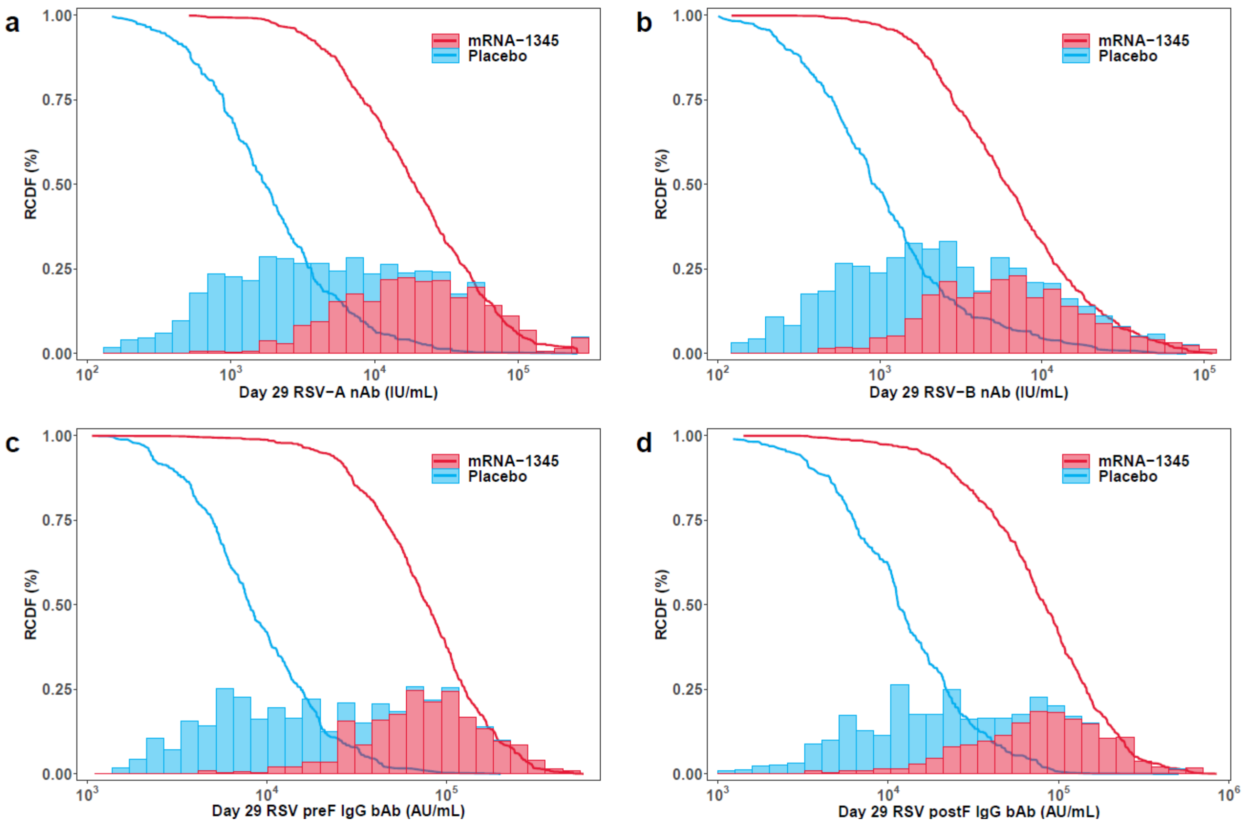

Fig. S15. Inverse probability of sampling weight adjusted reverse cumulative distribution function curves for each Day 29 antibody marker (RSV-A nAb [a], RSV-B nAb [b], RSV preF IgG bAb [c], and RSV postF IgG bAb [d]) in vaccine and placebo recipients in the Day 29 case-cohort set for RSV-LRTD-3+.

The inverse probability of sampling weight was calculated with respect to RSV-LRTD-3+. The stacked histograms display the inverse probability of sampling weight adjusted distribution of Day 29 antibody markers of vaccine (red) and placebo (blue).

bAb, binding antibody; IgG, immunoglobulin G; LRTD, lower respiratory tract disease; nAb, neutralizing antibody; postF, postfusion; preF, prefusion; RCDF, reverse cumulative distribution function; RSV, respiratory syncytial virus.

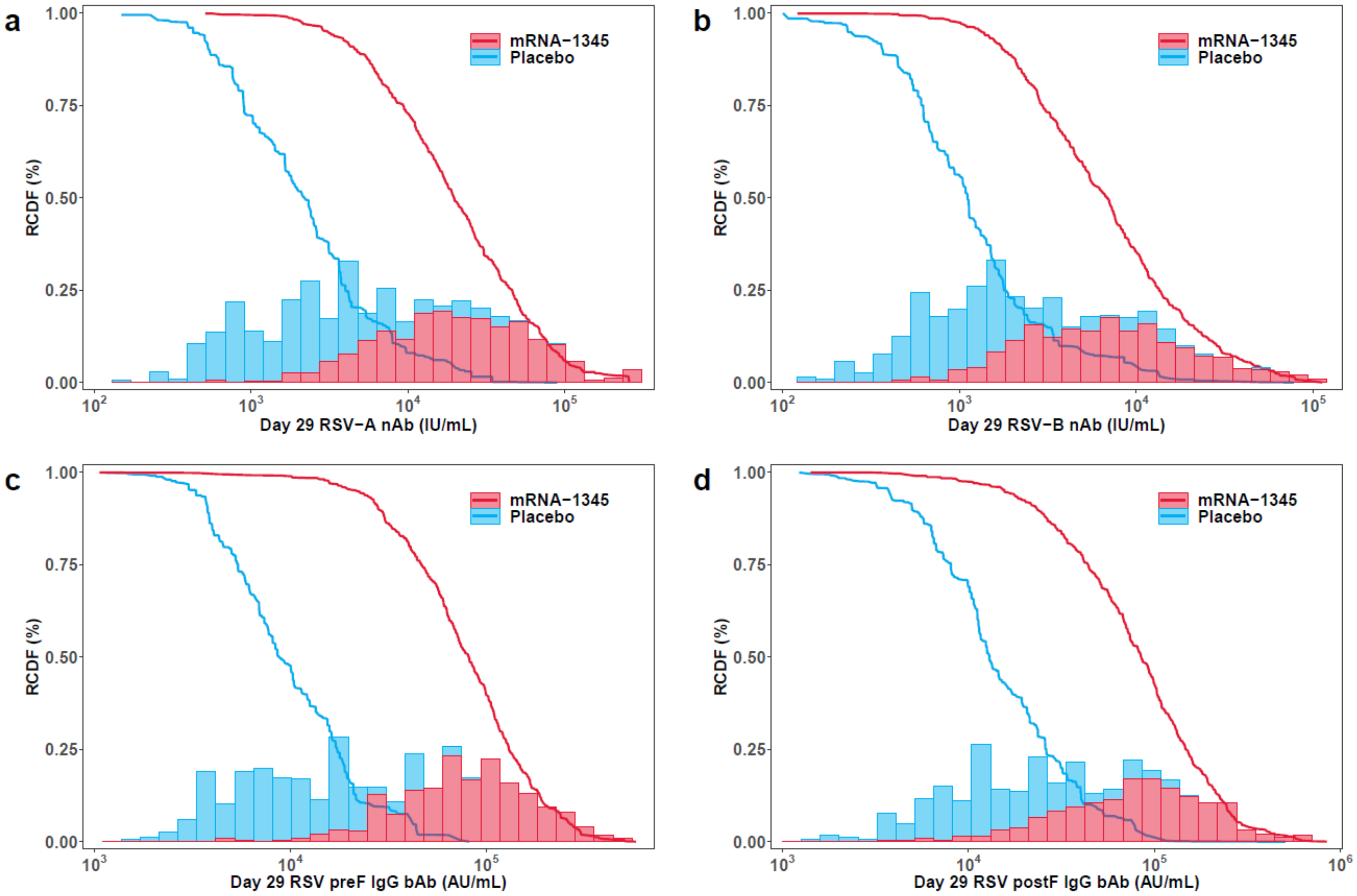

Fig. S16. Inverse probability of sampling weight adjusted reverse cumulative distribution function curves for each Day 29 antibody marker (RSV-A nAb [a], RSV-B nAb [b], RSV preF IgG bAb [c], and RSV postF IgG bAb [d]) in vaccine and placebo recipients in Day 29 case-cohort set for RSV-ARD.

The inverse probability of sampling weight was calculated with respect to RSV-ARD. The stacked histograms display the inverse probability of sampling weight adjusted distribution of Day 29 antibody markers of vaccine (red) and placebo (blue).

ARD, acute respiratory disease; bAb, binding antibody; IgG, immunoglobulin G; LRTD, lower respiratory tract disease; nAb, neutralizing antibody; postF, postfusion; preF, prefusion; RCDF, reverse cumulative distribution function; RSV, respiratory syncytial virus.

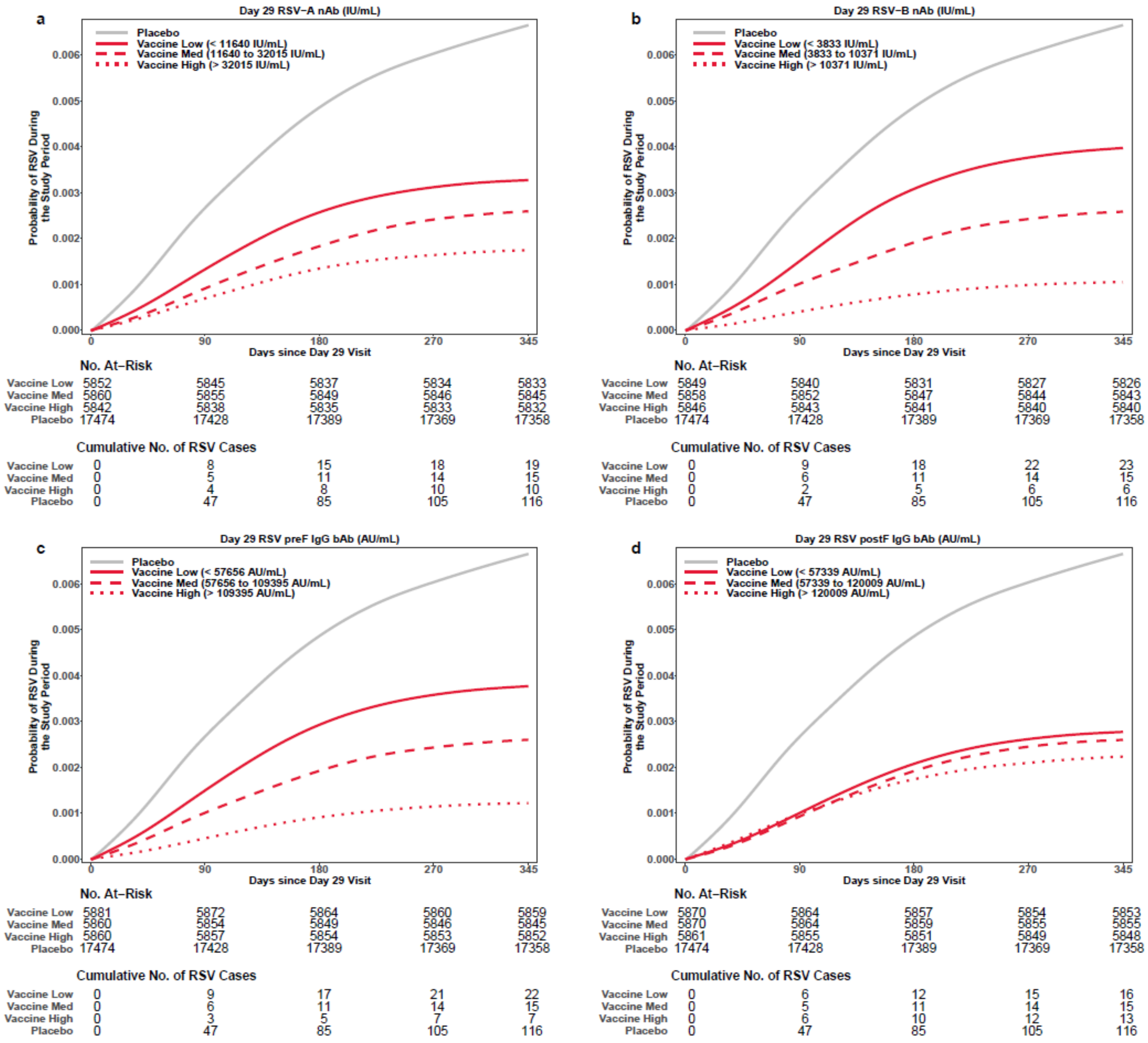

Fig. S17. Covariate-adjusted cumulative risk of RSV-LRTD-2+ in placebo recipients and in the low, medium, and high tertile of vaccine recipients by each Day 29 antibody marker (RSV-A nAb [a], RSV-B nAb [b], RSV preF IgG bAb [c], and RSV postF IgG bAb [d]) in the Day 29 case-cohort set for RSV-LRTD-2+.

Baseline risk factors were adjusted in the univariable (qualitative) inverse probability of sampling weighted Cox regression model, including the actual stratification factors age and LRTD at-risk, and baseline risk score.

bAb, binding antibody; IgG, immunoglobulin G; LRTD, lower respiratory tract disease; nAb, neutralizing antibody; postF, postfusion; preF, prefusion; RSV, respiratory syncytial virus.

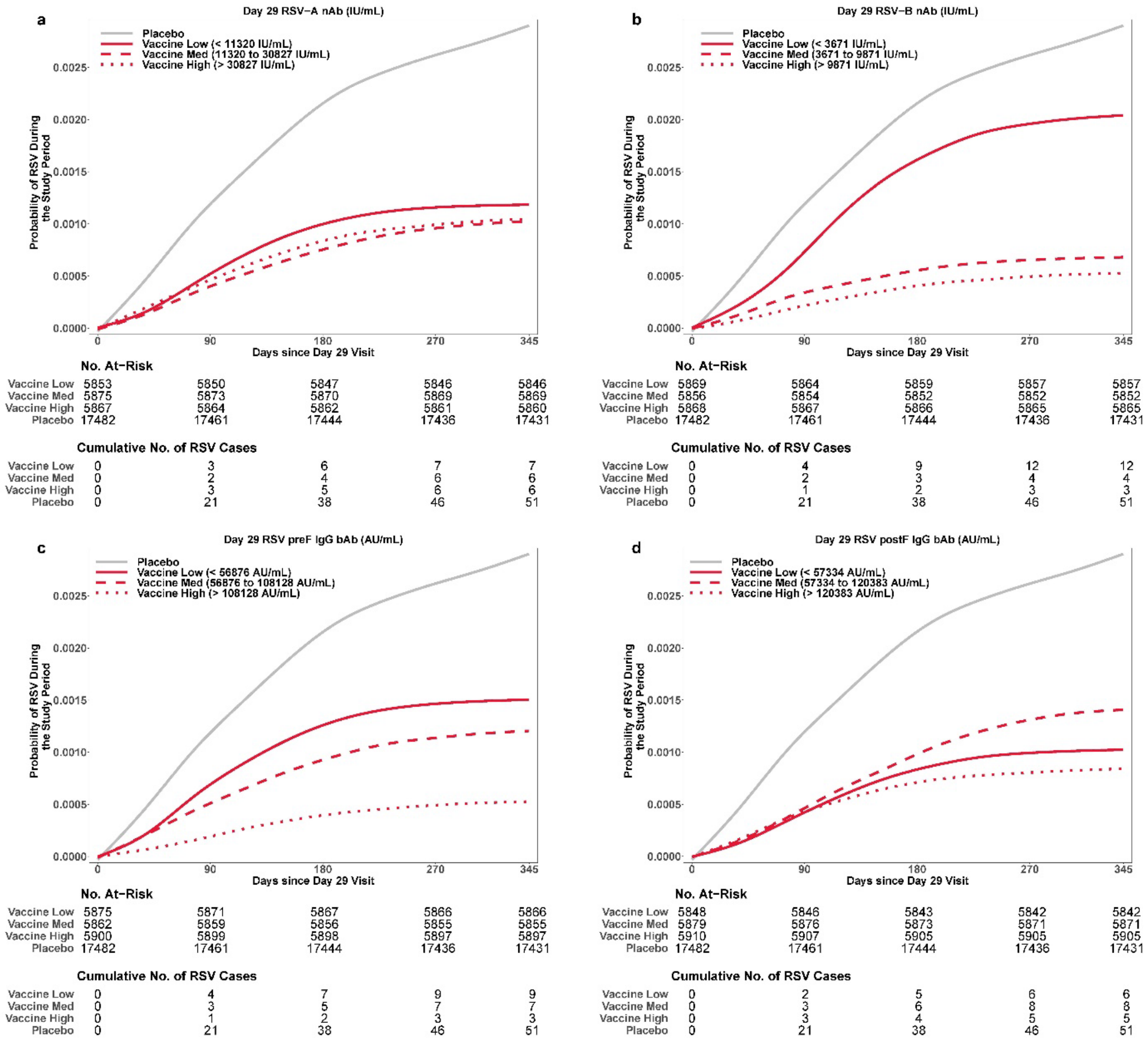

Fig. S18. Covariate-adjusted cumulative risk of RSV-LRTD-3+ in placebo recipients and in the low, medium, and high tertile of vaccine recipients by each Day 29 antibody marker (RSV-A nAb [a], RSV-B nAb [b], RSV preF IgG bAb [c], and RSV postF IgG bAb [d]) in the Day 29 case-cohort set for RSV-LRTD-3+.

Baseline risk factors were adjusted in the univariable (qualitative) inverse probability of sampling weighted Cox regression model, including the actual stratification factors age and LRTD at-risk, and baseline risk score.

bAb, binding antibody; IgG, immunoglobulin G; LRTD, lower respiratory tract disease; nAb, neutralizing antibody; postF, postfusion; preF, prefusion; RSV, respiratory syncytial virus.

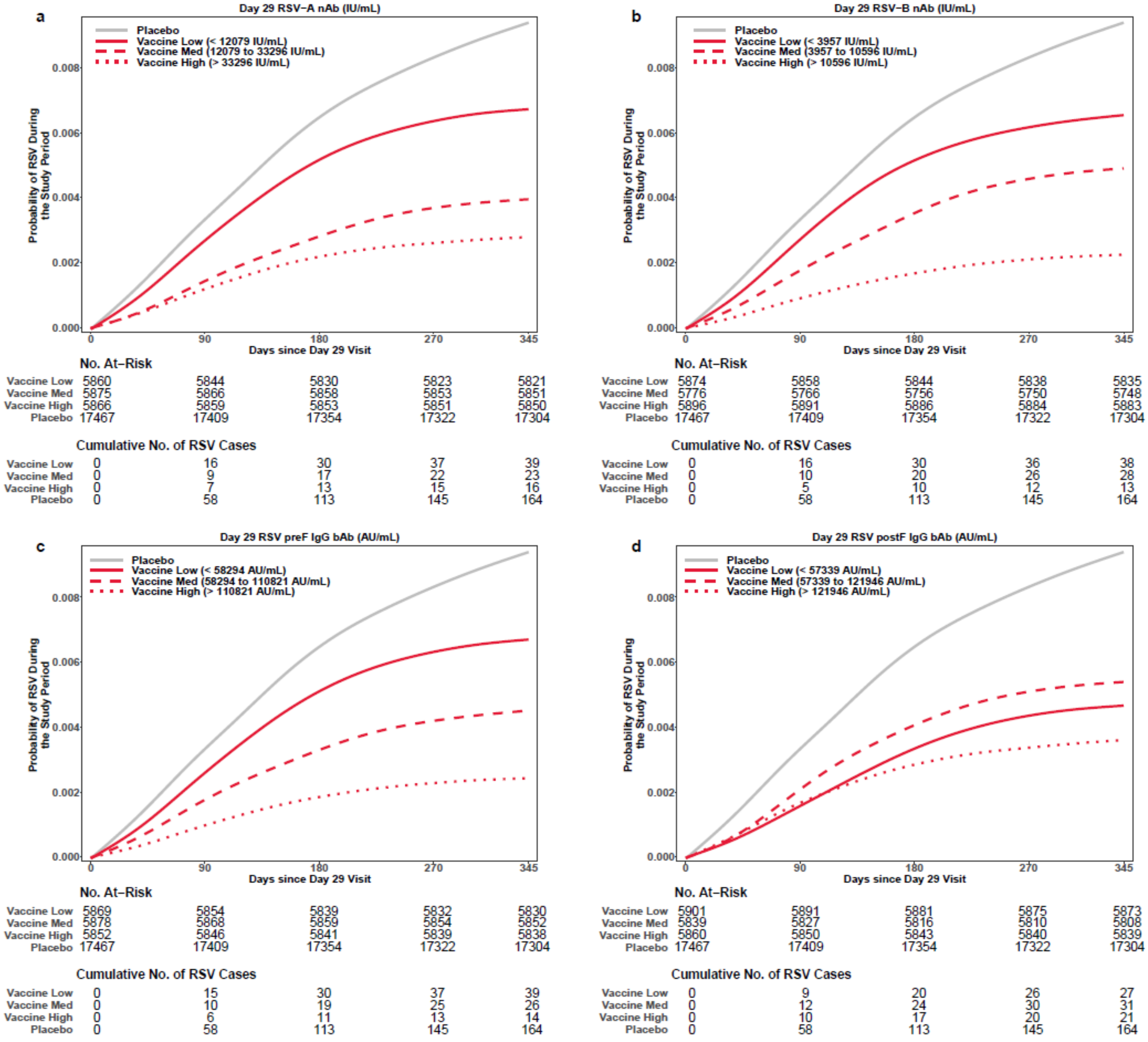

Fig. S19. Covariate-adjusted cumulative risk of RSV-ARD in placebo recipients and in the low, medium, and high tertile of vaccine recipients by each Day 29 antibody marker (RSV-A nAb [a], RSV-B nAb [b], RSV preF IgG bAb [c], and RSV postF IgG bAb [d]) in the Day 29 case-cohort set for RSV-ARD.

Baseline risk factors were adjusted in the univariable (qualitative) inverse probability of sampling weighted Cox regression model, including the actual stratification factors age and LRTD at-risk, and baseline risk score.

ARD, acute respiratory disease; bAb, binding antibody; IgG, immunoglobulin G; LRTD, lower respiratory tract disease; nAb, neutralizing antibody; postF, postfusion; preF, prefusion; RSV, respiratory syncytial virus.

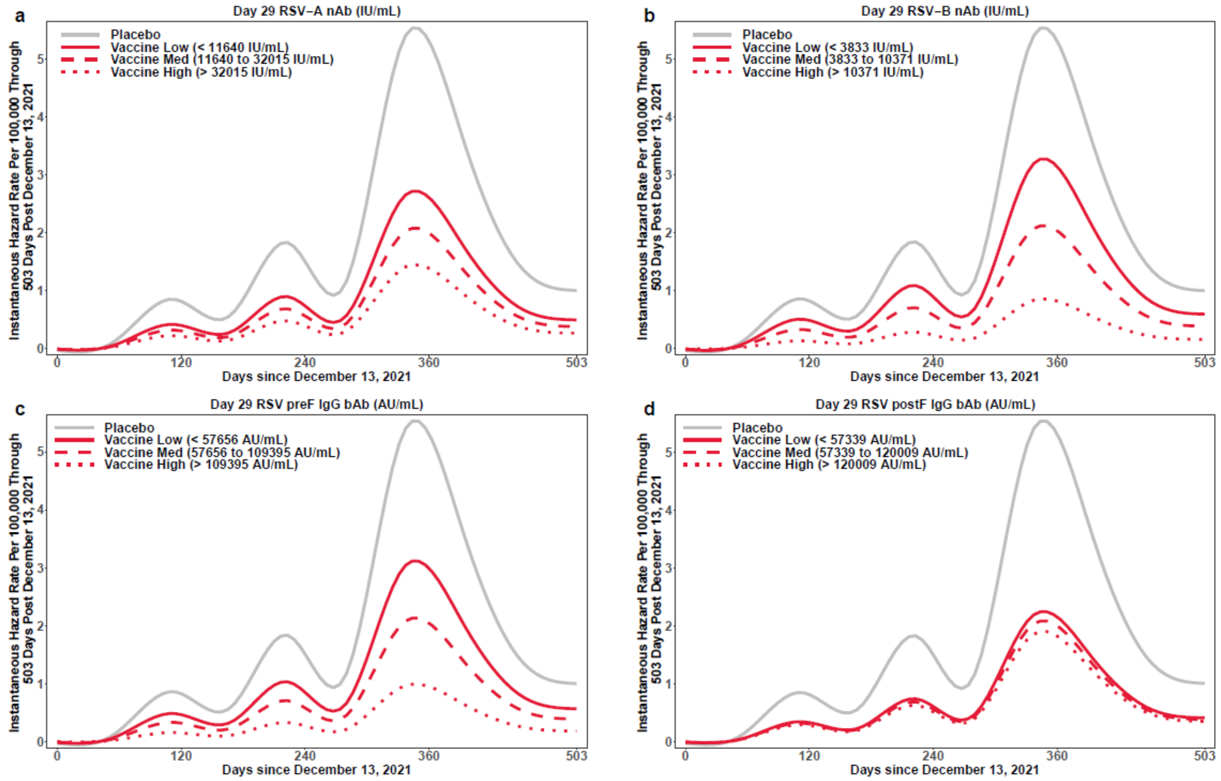

Fig. S20. Covariate-adjusted instantaneous hazard rate of RSV-LRTD-2+ in placebo recipients and in the low, medium, and high tertile of vaccine recipients by each Day 29 antibody marker (RSV-A nAb [a], RSV-B nAb [b], RSV preF IgG bAb [c], and RSV postF IgG bAb [d]) in the Day 29 case-cohort set for RSV-LRTD-2+ from December 13, 2021, through April 30, 2023.

Baseline risk factors were adjusted in the univariable (qualitative) inverse probability of sampling weighted Cox regression model, including the actual stratification factors age and LRTD at-risk, and baseline risk score.

bAb, binding antibody; IgG, immunoglobulin G; LRTD, lower respiratory tract disease; nAb, neutralizing antibody; postF, postfusion; preF, prefusion; RSV, respiratory syncytial virus.

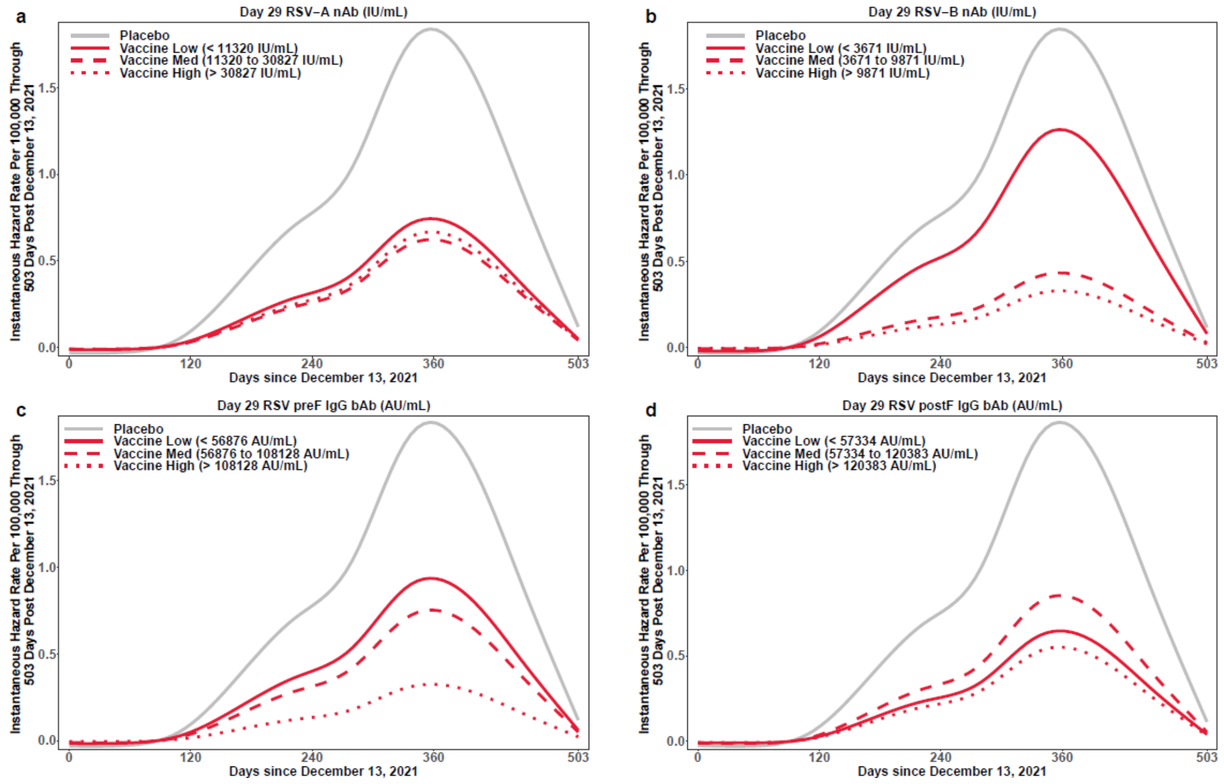

Fig. S21. Covariate-adjusted instantaneous hazard rate of RSV-LRTD-3+ in placebo recipients and in the low, medium, and high tertile of vaccine recipients by each Day 29 antibody marker (RSV-A nAb [a], RSV-B nAb [b], RSV preF IgG bAb [c], and RSV postF IgG bAb [d]) in the Day 29 case-cohort set for RSV-LRTD-3+ from December 13, 2021, through April 30, 2023.

Baseline risk factors were adjusted in the univariable (qualitative) inverse probability of sampling weighted Cox regression model, including the actual stratification factors age and LRTD at-risk, and baseline risk score.

bAb, binding antibody; IgG, immunoglobulin G; LRTD, lower respiratory tract disease; nAb, neutralizing antibody; postF, postfusion; preF, prefusion; RSV, respiratory syncytial virus.

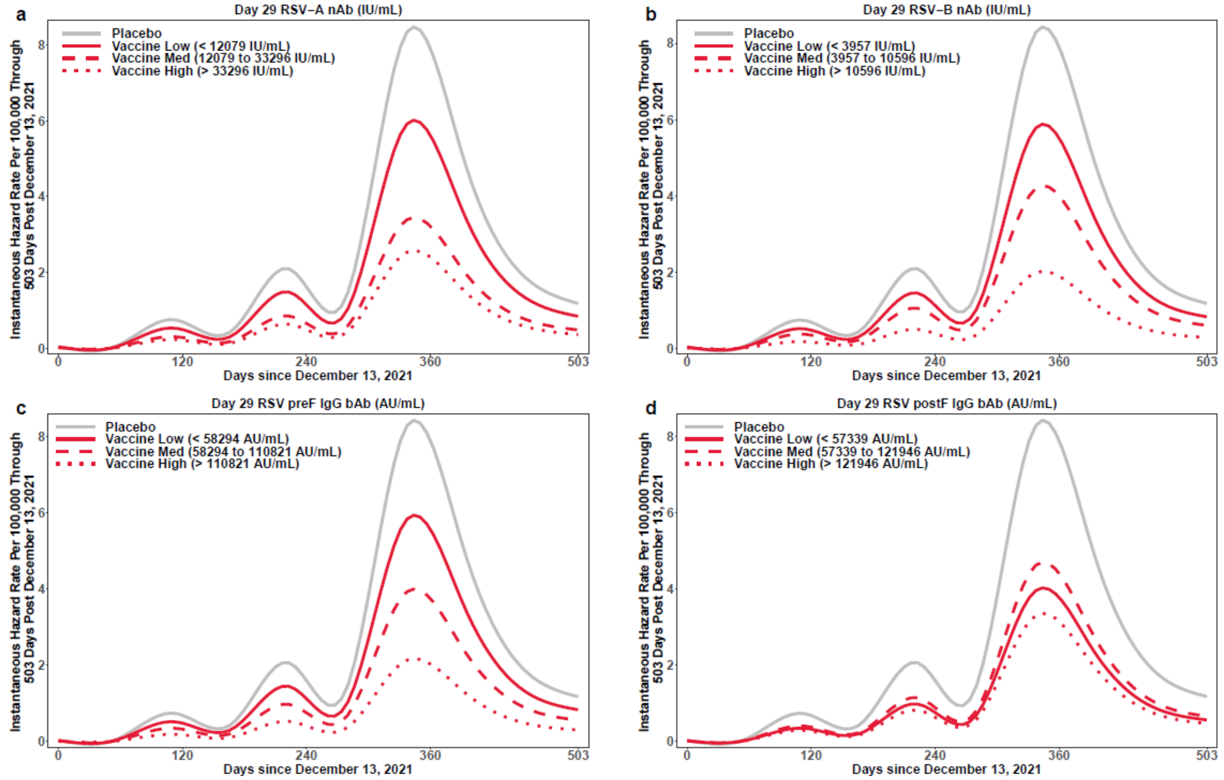

Fig. S22. Covariate-adjusted instantaneous hazard rate of RSV-ARD in placebo recipients and in the low, medium, and high tertiles of vaccine recipients by each Day 29 antibody marker (RSV-A nAb [a], RSV-B nAb [b], RSV preF IgG bAb [c], and RSV postF IgG bAb [d]) in the Day 29 case-cohort set for RSV-ARD from December 13, 2021, through April 30, 2023.

Baseline risk factors were adjusted in the univariable (qualitative) inverse probability of sampling weighted Cox regression model, including the actual stratification factors age and LRTD at-risk, and baseline risk score.

ARD, acute respiratory disease; bAb, binding antibody; IgG, immunoglobulin G; LRTD, lower respiratory tract disease; nAb, neutralizing antibody; postF, postfusion; preF, prefusion; RSV, respiratory syncytial virus.

a

Endpoint	Variable Set*	Treatment: HR (95% CI)	Treatment: P-value (2-Sided)	Treatment: FWER-Adjusted P-value	Day 29 Marker: HR (95% CI)	Day 29 Marker: P-value (2-Sided)	Day 29 Marker: FWER-Adjusted P-value
RSV-LRTD-2+	Treatment	0.36 (0.24,0.55)	<0.001	<0.001			
RSV-LRTD-2+	Treatment + RSV-A nAb	0.77 (0.46,1.30)	0.326	0.521	0.44 (0.30,0.65)	<0.001	<0.001
RSV-LRTD-2+	Treatment + RSV-B nAb	0.69 (0.39,1.22)	0.199	0.489	0.42 (0.25,0.72)	0.001	0.002
RSV-LRTD-2+	Treatment + RSV preF IgG bAb	0.82 (0.44,1.51)	0.521	0.521	0.40 (0.23,0.68)	0.001	0.002
RSV-LRTD-2+	Treatment + RSV postF IgG bAb	0.56 (0.33,0.96)	0.035	0.140	0.55 (0.33,0.89)	0.016	0.016

*Age, LRTD At-Risk, and baseline risk score are adjusted.

Hazard Ratio

b

Endpoint	Variable Set*	Treatment: HR (95% CI)	Treatment: P-value (2-Sided)	Treatment: FWER-Adjusted P-value	Day 29 Marker: HR (95% CI)	Day 29 Marker: P-value (2-Sided)	Day 29 Marker: FWER-Adjusted P-value
RSV-LRTD-3+	Treatment	0.27 (0.12,0.61)	0.001	0.005			
RSV-LRTD-3+	Treatment + RSV-A nAb	0.59 (0.23,1.51)	0.272	0.272	0.41 (0.20,0.84)	0.014	0.056
RSV-LRTD-3+	Treatment + RSV-B nAb	0.43 (0.15,1.21)	0.110	0.272	0.55 (0.25,1.24)	0.151	0.183
RSV-LRTD-3+	Treatment + RSV preF IgG bAb	0.51 (0.17,1.51)	0.226	0.272	0.47 (0.21,1.04)	0.063	0.183
RSV-LRTD-3+	Treatment + RSV postF IgG bAb	0.41 (0.15,1.11)	0.078	0.234	0.60 (0.28,1.28)	0.183	0.183

*Age, LRTD At-Risk, and baseline risk score are adjusted.

Hazard Ratio

c

Endpoint	Variable Set*	Treatment: HR (95% CI)	Treatment: P-value (2-Sided)	Treatment: FWER-Adjusted P-value	Day 29 Marker: HR (95% CI)	Day 29 Marker: P-value (2-Sided)	Day 29 Marker: FWER-Adjusted P-value
RSV-ARD	Treatment	0.52 (0.36,0.76)	0.001	0.005			
RSV-ARD	Treatment + RSV-A nAb	1.10 (0.65,1.88)	0.713	0.951	0.45 (0.28,0.71)	0.001	0.003
RSV-ARD	Treatment + RSV-B nAb	1.02 (0.60,1.72)	0.951	0.951	0.42 (0.25,0.73)	0.002	0.003
RSV-ARD	Treatment + RSV preF IgG bAb	1.29 (0.72,2.33)	0.389	0.951	0.36 (0.21,0.63)	<0.001	<0.001
RSV-ARD	Treatment + RSV postF IgG bAb	0.88 (0.54,1.42)	0.598	0.951	0.46 (0.28,0.77)	0.003	0.003

*Age, LRTD At-Risk, and baseline risk score are adjusted.

Hazard Ratio

Fig. S23. Forest plot of univariable inverse probability of sampling weighted Cox PH regression model summary for evaluating individual Day 29 antibody marker as a correlate of risk and protection against each RSV endpoint (RSV-LRTD-2+ [a], RSV-LRTD-3+ [b], and RSV-ARD [c]) in vaccine and placebo recipients in the corresponding Day 29 case-cohort set.

In each panel, the rows highlighted in yellow represent the marginal treatment (vaccine) effect, with the below rows representing the conditional treatment effect with a specific Day 29 marker. Rows highlighted in blue represent the top-performance CoRs/CoPs. Hazard ratios for the treatment (vaccine) effect of each model are shown on the left, while hazard ratios for the Day 29 marker effect are shown on the right. Baseline risk factors were adjusted in the univariable inverse probability of sampling weighted Cox regression model, including the actual stratification factors age and LRTD at-risk, and baseline risk score.

ARD, acute respiratory disease; bAb, binding antibody; CI, confidence interval; CoP, correlate of protection; CoR, correlate of risk; FWER, family-wise error rate; HR, hazard ratio; IgG, immunoglobulin G; LRTD, lower respiratory tract disease; nAb, neutralizing antibody; PH, proportional hazards; postF, postfusion; preF, prefusion; RSV, respiratory syncytial virus.

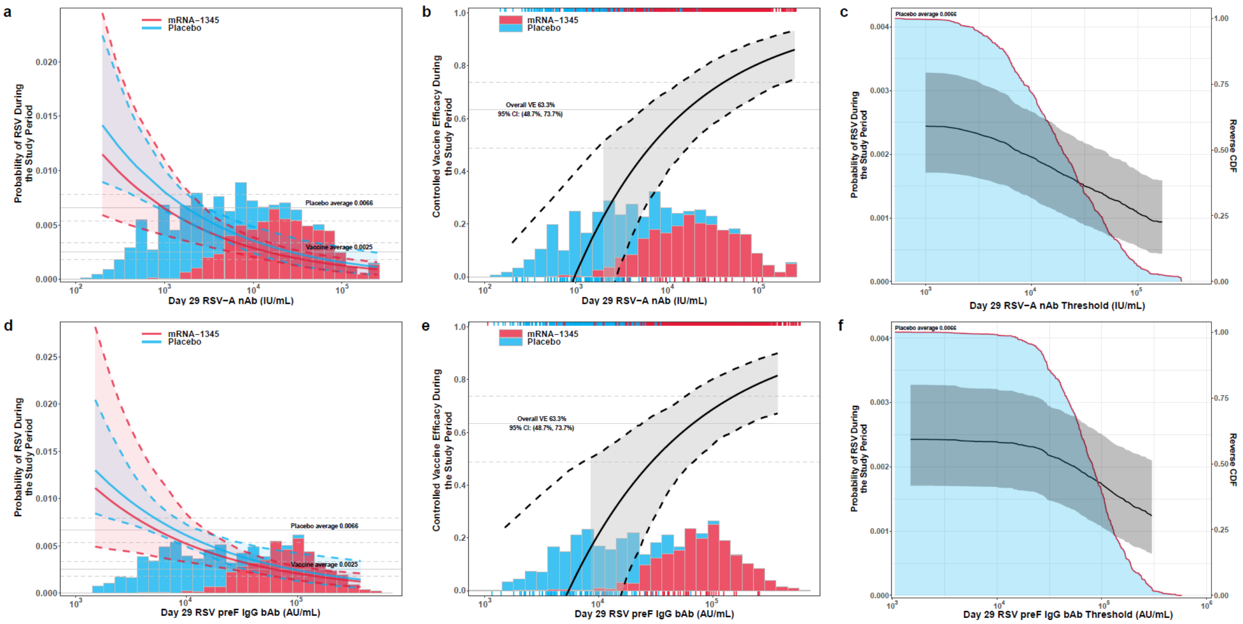

Fig. S24. (a-c) and (d-f) demonstrate further CoR/CoP analysis for RSV-LRTD-2+ by Day 29 RSV-A nAb and Day 29 RSV preF IgG bAb, respectively.

(a) and (d): The solid red and blue curves demonstrate the point estimates of the cumulative incidence of RSV-LRTD-2+ during the study period for vaccine and placebo recipients across a range of assigned antibody titers or concentration levels (within 0.5^{th} to 99.5^{th} percentiles of observed antibody values in both vaccine and placebo groups). The dashed red and blue curves along with the shading represent the bootstrap pointwise 95% CIs. The solid and dashed horizontal gray lines represent the point estimates and 95% CIs of the average covariate-adjusted cumulative incidence of RSV-LRTD-2+ in vaccine and placebo recipients. (b) and (e): The solid black curve shows the point estimate of controlled vaccine efficacy across a range of assigned antibody titers or concentration levels (within 0.5^{th} to 99.5^{th} percentiles of observed antibody values in both vaccine and placebo groups), and the dashed black curves demonstrate the bootstrap pointwise 95% CIs. The rug lines at the bottom and top represent the RSV-LRTD-2+ cases and non-cases by vaccination status, respectively. The shaded gray area between the dashed curves highlights the VE above the median of antibody level in placebo recipients. The solid and dashed horizontal gray lines are the point estimates and 95% CIs of clinical vaccine efficacy in the additional analysis. (c) and (f): The red curve and the blue area below it represent the reverse cumulative density function values for the observed antibody marker values in vaccine recipients. The black curve is the covariate-adjusted cumulative incidence of RSV-LRTD-2+ during the study period across a range of antibody marker levels (below the 97.5^{th} percentile of observed antibody values in vaccine group). The shadowed gray area is the bootstrap pointwise 95% CI. The stacked histogram of the observed antibody marker titers or concentration levels by vaccination status is overlayed on the bottom of the cumulative incidence plots (a, d) and VE plots (b, e). Baseline covariates age, LRTD at-risk, and baseline risk score were adjusted in the inverse probability of sampling weighted Cox regression model.

bAb, binding antibody; CDF, cumulative distribution function; CI, confidence interval; CoP, correlate of protection; CoR, correlate of risk; IgG, immunoglobulin G; LRTD, lower respiratory

tract disease; nAb, neutralizing antibody; preF, prefusion; RSV, respiratory syncytial virus; VE, vaccine efficacy.

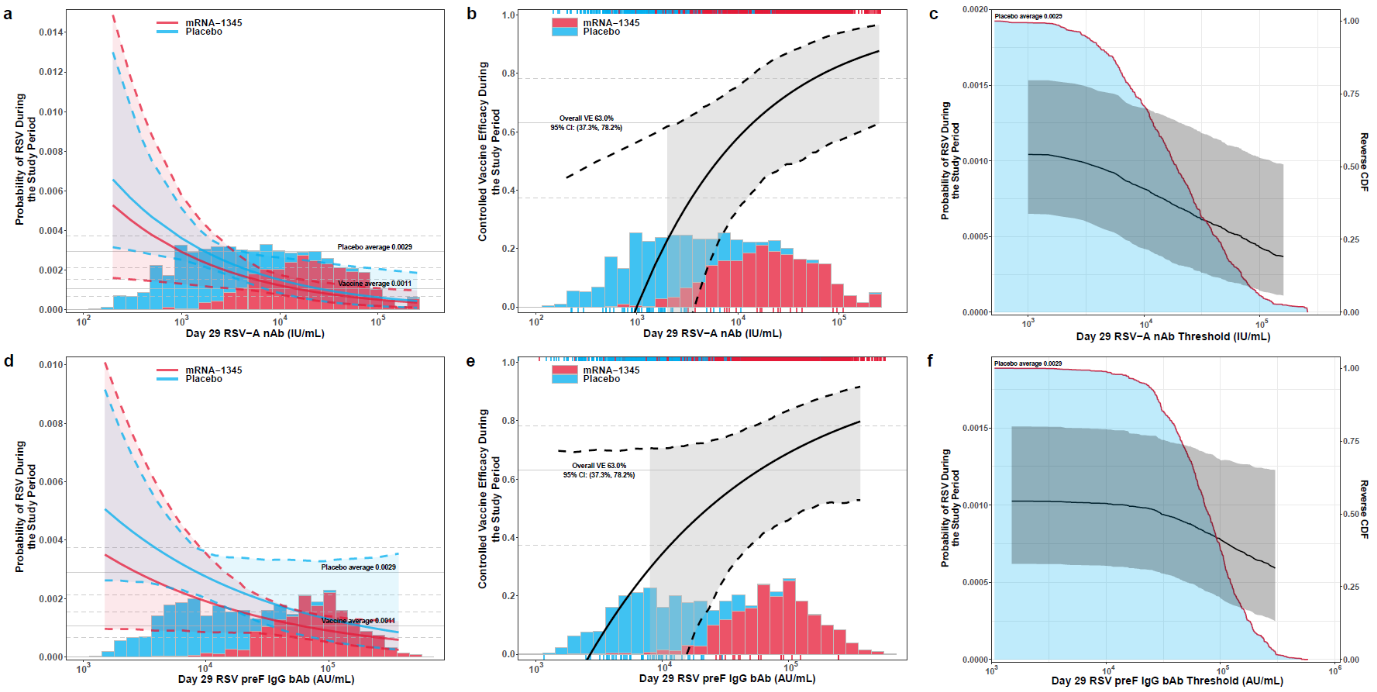

Fig. S25. (a-c) and (d-f) demonstrate further CoR/CoP analysis for RSV-LRTD-3+ by Day 29 RSV-A nAb and Day 29 RSV preF IgG bAb, respectively.

(a) and (d): The solid red and blue curves demonstrate the point estimates of the cumulative incidence of RSV-LRTD-3+ during the study period for vaccine and placebo recipients across a range of assigned antibody titers or concentration levels (within 0.5th to 99.5th percentiles of observed antibody values in both vaccine and placebo groups). The dashed red and blue curves along with shading represent the bootstrap pointwise 95% CIs. The solid and dashed horizontal gray lines represent the point estimates and 95% CIs of the average covariate-adjusted cumulative incidence of RSV-LRTD-3+ in vaccine and placebo recipients. (b) and (e): The solid black curve shows the point estimate of controlled vaccine efficacy across a range of assigned antibody titers or concentration levels (within 0.5th to 99.5th percentiles of observed antibody values in both vaccine and placebo groups), and the dashed black curves demonstrate the bootstrap pointwise 95% CIs. The rug lines at the bottom and top represent the RSV-LRTD-3+ cases and non-cases by vaccination status, respectively. The shaded gray area between the dashed curves highlights the VE above the median of antibody level in placebo recipients. The solid and dashed horizontal gray lines are the point estimates and 95% CIs of clinical vaccine efficacy in the additional analysis. (c) and (f): The red curve and the blue area below it represent the reverse cumulative density function values for the observed antibody marker values in vaccine recipients. The black curve is the covariate-adjusted cumulative incidence of RSV-LRTD-3+ during the study period t across a range of antibody marker levels (below 97.5th percentile of observed antibody values in vaccine group). The shadowed gray area is the bootstrap pointwise 95% CIs. The stacked histogram of the observed antibody marker titers or concentration levels by vaccination status is overlayed on the bottom of cumulative incidence plots (a, d) and VE plots (b, e). Baseline covariates age, LRTD at-risk, and baseline risk score were adjusted in the inverse probability of sampling weighted Cox regression model.

bAb, binding antibody; CDF, cumulative distribution function; CI, confidence interval; CoP, correlate of protection; CoR, correlate of risk; IgG, immunoglobulin G; LRTD, lower respiratory tract disease; nAb, neutralizing antibody; preF, prefusion; RSV, respiratory syncytial virus; VE, vaccine efficacy.

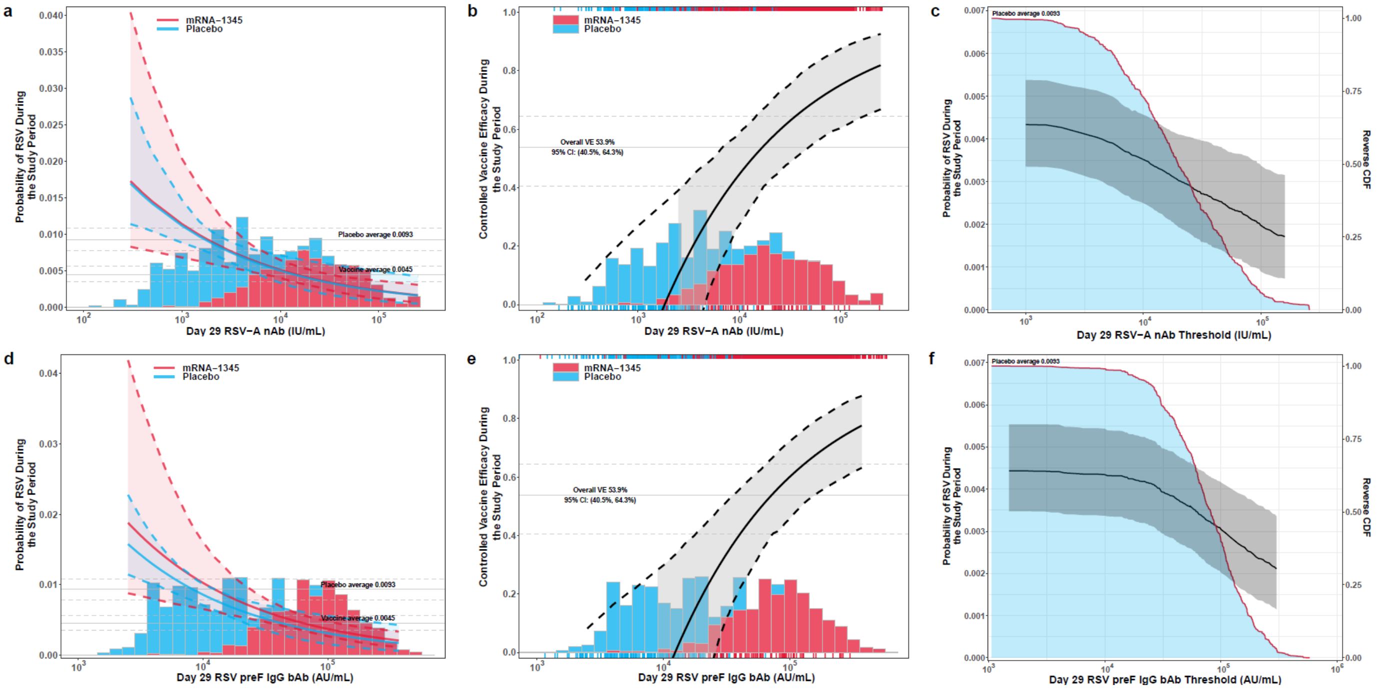

Fig. S26. (a-c) and (d-f) demonstrate further CoR/CoP analysis for RSV-ARD by Day 29 RSV-A nAb and Day 29 RSV preF IgG bAb, respectively.

(a) and (d): The solid red and blue curves demonstrate the point estimates of the cumulative incidence of RSV-ARD during the study period for vaccine and placebo recipients across a range of assigned antibody titers or concentration levels (within 0.5th to 99.5th percentiles of observed antibody values in both vaccine and placebo groups). The dashed red and blue curves along with the shading represent the bootstrap pointwise 95% CIs. The solid and dashed horizontal gray lines represent the point estimates and 95% CIs of the average covariate-adjusted cumulative incidence of RSV-ARD in vaccine and placebo recipients. (b) and (e): The solid black curve shows the point estimate of controlled vaccine efficacy across a range of assigned antibody titers or concentration levels (within 0.5th to 99.5th percentiles of observed antibody values in both vaccine and placebo groups), and the dashed black curves demonstrate the bootstrap pointwise 95% CIs. The rug lines at the bottom and top represent the RSV-ARD cases and non-cases by vaccination status, respectively. The shaded gray area between the dashed curves highlights the VE above the median of antibody level in placebo recipients. The solid and dashed horizontal gray lines are the point estimates and 95% CIs of clinical vaccine efficacy in the additional analysis. (c) and (f): The red curve and the blue area below it represent the reverse cumulative density function values for the observed antibody marker values in vaccine recipients. The black curve is the covariate-adjusted cumulative incidence of RSV-ARD during the study period across a range of antibody marker levels (below 97.5th percentile of observed antibody values in vaccine group). The shadowed gray area is the bootstrap pointwise 95% CIs. The stacked histogram of the observed antibody marker titers or concentration levels by vaccination status is overlayed on the bottom of the cumulative incidence plots (a, d) and VE plots (b, e). Baseline covariates age, LRTD at-risk, and baseline risk score were adjusted in the inverse probability of sampling weighted Cox regression model.

ARD, acute respiratory disease; bAb, binding antibody; CDF, cumulative distribution function; CI, confidence interval; CoP, correlate of protection; CoR, correlate of risk; IgG, immunoglobulin G; LRTD, lower respiratory tract disease; nAb, neutralizing antibody; preF, prefusion; RSV, respiratory syncytial virus; VE, vaccine efficacy.

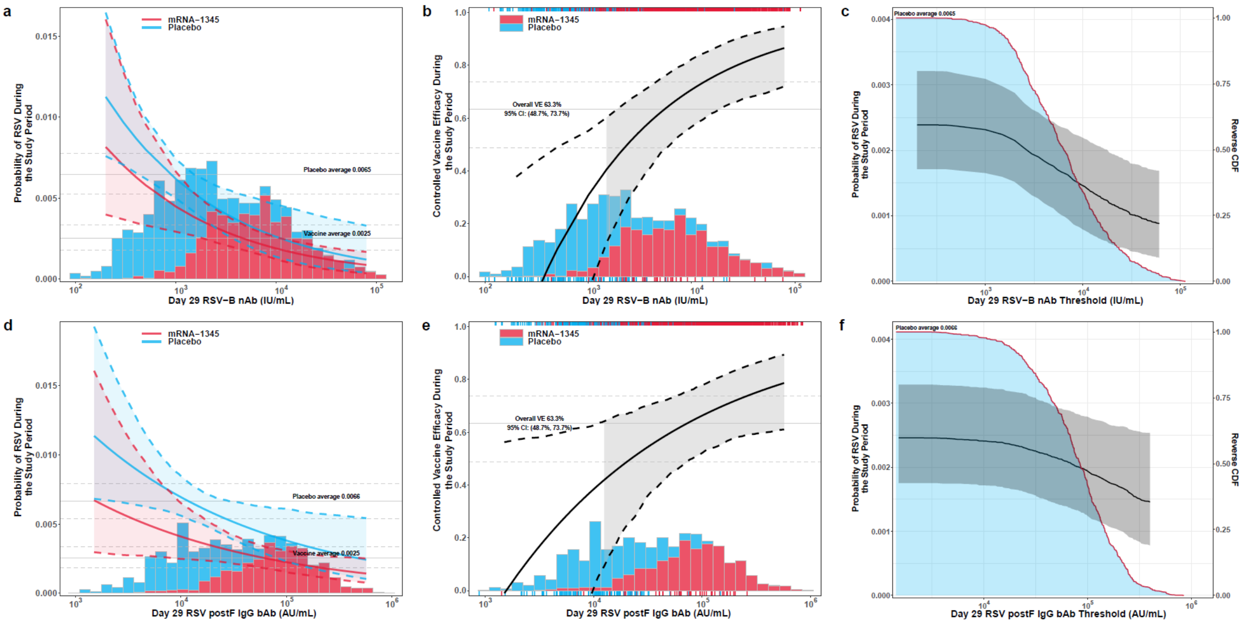

Fig. S27. (a-c) and (d-f) demonstrate further CoR/CoP analysis for RSV-LRTD-2+ by Day 29 RSV-B nAb and Day 29 RSV postF IgG bAb, respectively.

(a) and (d): The solid red and blue curves demonstrate the point estimates of the cumulative incidence of RSV-LRTD-2+ during the study period for vaccine and placebo recipients across a range of assigned antibody titers or concentration levels (within 0.5^{th} to 99.5^{th} percentiles of observed antibody values in both vaccine and placebo groups). The dashed red and blue curves along with the shading represent the bootstrap pointwise 95% CIs. The solid and dashed horizontal gray lines represent the point estimates and 95% CIs of the average covariate-adjusted cumulative incidence of RSV-LRTD-2+ in vaccine and placebo recipients. (b) and (e): The solid black curve shows the point estimate of controlled vaccine efficacy across a range of assigned antibody titers or concentration levels (within 0.5^{th} to 99.5^{th} percentiles of observed antibody values in both vaccine and placebo groups), and the dashed black curves demonstrate the bootstrap pointwise 95% CIs. The rug lines at the bottom and top represent the RSV-LRTD-2+ cases and non-cases by vaccination status, respectively. The shaded gray area between the dashed curves highlights the VE above the median of antibody level in placebo recipients. The solid and dashed horizontal gray lines are the point estimates and 95% CIs of clinical vaccine efficacy in the additional analysis. (c) and (f): The red curve and the blue area below it represents the reverse cumulative density function values for the observed antibody marker values in vaccine recipients. The black curve is the covariate-adjusted cumulative incidence of RSV-LRTD-2+ during the study period across a range of antibody marker levels (below 97.5^{th} percentile of observed antibody values in vaccine group). The shadowed gray area is the bootstrap pointwise 95% CIs. The stacked histogram of the observed antibody marker titers or concentration levels by vaccination status is overlayed on the bottom of cumulative incidence plots (a, d) and VE plots (b, e). Baseline covariates age, LRTD at-risk, and baseline risk score were adjusted in the inverse probability of sampling weighted Cox regression model.

bAb, binding antibody; CDF, cumulative distribution function; CI, confidence interval; CoP, correlate of protection; CoR, correlate of risk; IgG, immunoglobulin G; LRTD, lower respiratory

tract disease; nAb, neutralizing antibody; postF, postfusion; RSV, respiratory syncytial virus; VE, vaccine efficacy.

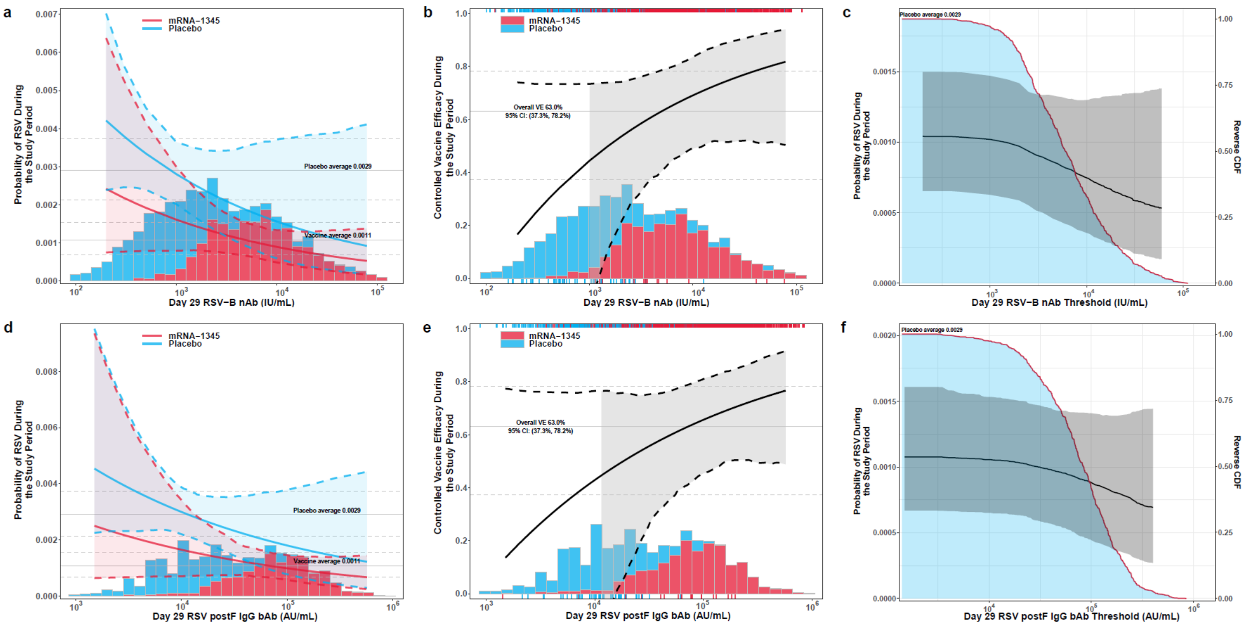

Fig. S28. (a-c) and (d-f) demonstrate further CoR/CoP analysis for RSV-LRTD-3+ by Day 29 RSV-B nAb and Day 29 RSV postF IgG bAb, respectively.

(a) and (d): The solid red and blue curves demonstrate the point estimates of the cumulative incidence of RSV-LRTD-3+ during the study period for vaccine and placebo recipients across a range of assigned antibody titers or concentration levels (within 0.5th to 99.5th percentiles of observed antibody values in both vaccine and placebo groups). The dashed red and blue curves along with the shading represent the bootstrap pointwise 95% CIs. The solid and dashed horizontal gray lines represent the point estimates and 95% CIs of the average covariate-adjusted cumulative incidence of RSV-LRTD-3+ in vaccine and placebo recipients. (b) and (e): The solid black curve shows the point estimate of controlled vaccine efficacy across a range of assigned antibody titers or concentration levels (within 0.5th to 99.5th percentiles of observed antibody values in both vaccine and placebo groups), and the dashed black curves demonstrate the bootstrap pointwise 95% CIs. The rug lines at the bottom and top represent the RSV-LRTD-3+ cases and non-cases by vaccination status, respectively. The shaded gray area between the dashed curves highlights the VE above the median of antibody level in placebo recipients. The solid and dashed horizontal gray lines are the point estimates and 95% CIs of clinical vaccine efficacy in the additional analysis. (c) and (f): The red curve and the blue area below it represents the reverse cumulative density function values for the observed antibody marker values in vaccine recipients. The black curve is the covariate-adjusted cumulative incidence of RSV-LRTD-3+ during the study period across a range of antibody marker levels (below 97.5th percentile of observed antibody values in vaccine group). The shadowed gray area is the bootstrap pointwise 95% CIs. The stacked histogram of the observed antibody marker titers or concentration levels by vaccination status is overlayed on the bottom of cumulative incidence plots (a, d) and VE plots (b, e). Baseline covariates age, LRTD at-risk, and baseline risk score were adjusted in the inverse probability of sampling weighted Cox regression model.

bAb, binding antibody; CDF, cumulative distribution function; CI, confidence interval; CoP, correlate of protection; CoR, correlate of risk; IgG, immunoglobulin G; LRTD, lower respiratory

tract disease; nAb, neutralizing antibody; postF, postfusion; RSV, respiratory syncytial virus; VE, vaccine efficacy.

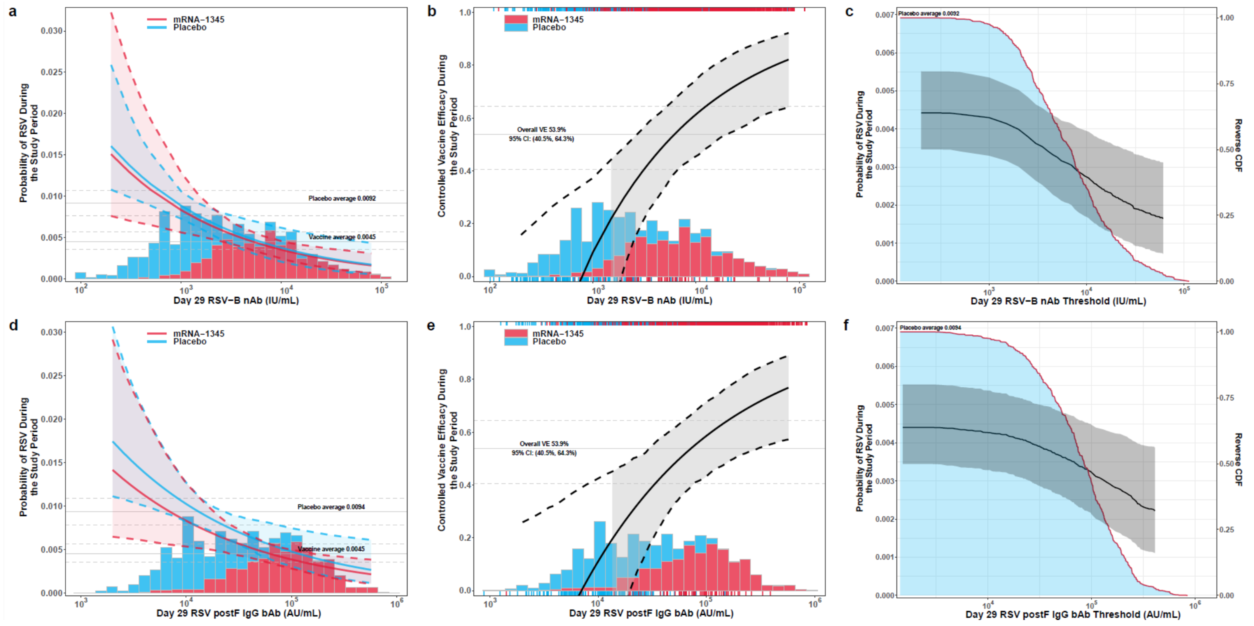

Fig. S29. (a-c) and (d-f) demonstrates further CoR/CoP analysis for RSV-ARD by Day 29 RSV-B nAb and Day 29 RSV postF IgG bAb, respectively.

(a) and (d): The solid red and blue curves demonstrate the point estimates of the cumulative incidence of RSV-ARD during the study period for vaccine and placebo recipients across a range of assigned antibody titers or concentration levels (within 0.5th to 99.5th percentiles of observed antibody values in both vaccine and placebo groups). The dashed red and blue curves along with the shading represent the bootstrap pointwise 95% CIs. The solid and dashed horizontal gray lines represent the point estimates and 95% CIs of the average covariate-adjusted cumulative incidence of RSV-ARD in vaccine and placebo recipients. (b) and (e): The solid black curve shows the point estimate of controlled vaccine efficacy across a range of assigned antibody titers or concentration levels (within 0.5th to 99.5th percentiles of observed antibody values in both vaccine and placebo groups), and the dashed black curves demonstrate the bootstrap pointwise 95% CIs. The rug lines at the bottom and top represent the RSV-ARD cases and non-cases by vaccination status, respectively. The shaded gray area between dashed curves highlights the VE above the median of antibody level in placebo recipients. The solid and the dashed horizontal gray lines are the point estimates and 95% CIs of clinical vaccine efficacy in the additional analysis. (c) and (f): The red curve and the blue area below it represent the reverse cumulative density function values for the observed antibody marker values in vaccine recipients. The black curve is the covariate-adjusted cumulative incidence of RSV-ARD during the study period across a range of antibody marker levels (below 97.5th percentile of observed antibody values in vaccine group). The shadowed gray area is the bootstrap pointwise 95% confidence intervals. The stacked histogram of the observed antibody marker titers or concentration levels by vaccination status is overlayed on the bottom of cumulative incidence plots (a, d) and VE plots (b, e). Baseline covariates age, LRTD at-risk, and baseline risk score were adjusted in the inverse probability of sampling weighted Cox regression model.

ARD, acute respiratory disease; bAb, binding antibody; CDF, cumulative distribution function; CI, confidence interval; CoP, correlate of protection; CoR, correlate of risk; IgG,

immunoglobulin G; LRTD, lower respiratory tract disease; nAb, neutralizing antibody; postF, postfusion; RSV, respiratory syncytial virus; VE, vaccine efficacy.

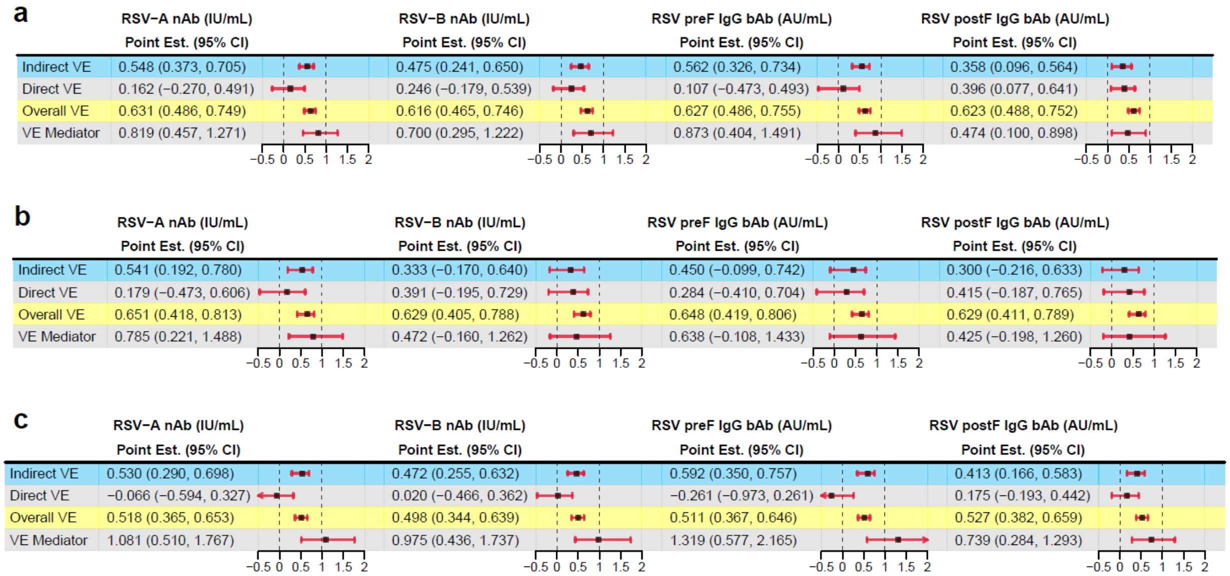

Fig. S30. (a-c) demonstrate forest plots of the mediation analysis summary for each Day 29 antibody marker and each RSV endpoint (RSV-LRTD-2+ [a], RSV-LRTD-3+ [b], and RSV-ARD [c]) in the Day 29 case-cohort set.

Each forest plot shows the point estimate and bootstrap 95% CI of the indirect vaccine effect (blue), direct vaccine effect, overall vaccine effect (yellow), and vaccine efficacy mediator (percent of vaccine efficacy explained by the antibody marker) for each individual Day 29 antibody marker and each RSV endpoint, respectively.

ARD, acute respiratory disease; bAb, binding antibody; CI, confidence interval; Est, estimate; IgG, immunoglobulin G; LRTD, lower respiratory tract disease; nAb, neutralizing antibody; preF, prefusion; RSV, respiratory syncytial virus; VE, vaccine efficacy.

a

RSV-LRTD-2+	Variable Set*	Treatment HR (95% CI)	Treatment P-value (2-Sided)	Treatment FWER-Adjusted P-value	Day 29 Marker HR (95% CI)	Day 29 Marker P-value (2-Sided)	Day 29 Marker FWER-Adjusted P-value
RSV Subtype A	Treatment	0.31 (0.18,0.53)	<0.001	<0.001			
RSV Subtype A	Treatment + RSV-A nAb	0.71 (0.38,1.35)	0.301	0.480	0.39 (0.24,0.65)	<0.001	<0.001
RSV Subtype A	Treatment + RSV-B nAb	0.46 (0.23,0.95)	0.035	0.140	0.56 (0.29,1.11)	0.096	0.096
RSV Subtype A	Treatment + RSV preF IgG bAb	0.76 (0.36,1.63)	0.480	0.480	0.36 (0.18,0.69)	0.002	0.006
RSV Subtype A	Treatment + RSV postF IgG bAb	0.55 (0.28,1.07)	0.080	0.240	0.44 (0.25,0.80)	0.007	0.014
RSV Subtype B	Treatment	0.42 (0.24,0.73)	0.003	0.015			
RSV Subtype B	Treatment + RSV-A nAb	0.84 (0.42,1.68)	0.628	0.814	0.48 (0.30,0.75)	0.001	0.003
RSV Subtype B	Treatment + RSV-B nAb	1.10 (0.51,2.33)	0.813	0.814	0.28 (0.14,0.57)	<0.001	<0.001
RSV Subtype B	Treatment + RSV preF IgG bAb	0.91 (0.41,2.03)	0.814	0.814	0.42 (0.22,0.79)	0.007	0.014
RSV Subtype B	Treatment + RSV postF IgG bAb	0.53 (0.26,1.10)	0.089	0.356	0.71 (0.38,1.34)	0.292	0.292

*Age, LRTD At-Risk, and baseline risk score are adjusted.

Hazard Ratio (Treatment axis: 0, 0.5, 1, 1.5, 2); Hazard Ratio (Day 29 Marker axis: 0, 0.2, 0.4, 0.6, 0.8, 1)

b

RSV-LRTD-3+	Variable Set*	Treatment HR (95% CI)	Treatment P-value (2-Sided)	Treatment FWER-Adjusted P-value	Day 29 Marker HR (95% CI)	Day 29 Marker P-value (2-Sided)	Day 29 Marker FWER-Adjusted P-value
RSV Subtype A	Treatment	0.24 (0.09,0.65)	0.005	0.025			
RSV Subtype A	Treatment + RSV-A nAb	0.73 (0.24,2.25)	0.587	0.587	0.26 (0.09,0.71)	0.009	0.036
RSV Subtype A	Treatment + RSV-B nAb	0.32 (0.08,1.26)	0.102	0.306	0.68 (0.22,2.14)	0.510	0.510
RSV Subtype A	Treatment + RSV preF IgG bAb	0.51 (0.15,1.78)	0.293	0.586	0.38 (0.14,1.06)	0.065	0.195
RSV Subtype A	Treatment + RSV postF IgG bAb	0.34 (0.10,1.13)	0.078	0.234	0.64 (0.27,1.53)	0.314	0.510
RSV Subtype B	Treatment	0.32 (0.12,0.86)	0.024	0.120			
RSV Subtype B	Treatment + RSV-A nAb	0.49 (0.16,1.46)	0.201	0.433	0.64 (0.30,1.37)	0.250	0.333
RSV Subtype B	Treatment + RSV-B nAb	0.60 (0.17,2.15)	0.433	0.433	0.44 (0.17,1.16)	0.099	0.333
RSV Subtype B	Treatment + RSV preF IgG bAb	0.55 (0.14,2.23)	0.404	0.433	0.53 (0.20,1.45)	0.217	0.333
RSV Subtype B	Treatment + RSV postF IgG bAb	0.48 (0.13,1.83)	0.283	0.433	0.60 (0.21,1.70)	0.333	0.333

*Age, LRTD At-Risk, and baseline risk score are adjusted.

Hazard Ratio (Treatment axis: 0, 0.5, 1, 1.5, 2); Hazard Ratio (Day 29 Marker axis: 0, 0.2, 0.4, 0.6, 0.8, 1)

c

RSV-ARD	Variable Set*	Treatment HR (95% CI)	Treatment P-value (2-Sided)	Treatment FWER-Adjusted P-value	Day 29 Marker HR (95% CI)	Day 29 Marker P-value (2-Sided)	Day 29 Marker FWER-Adjusted P-value
RSV Subtype A	Treatment	0.48 (0.30,0.76)	0.002	0.010			
RSV Subtype A	Treatment + RSV-A nAb	1.09 (0.58,2.05)	0.790	0.790	0.42 (0.24,0.72)	0.002	0.004
RSV Subtype A	Treatment + RSV-B nAb	0.81 (0.43,1.53)	0.519	0.790	0.50 (0.26,0.94)	0.031	0.031
RSV Subtype A	Treatment + RSV preF IgG bAb	1.26 (0.63,2.52)	0.517	0.790	0.34 (0.18,0.65)	0.001	0.004
RSV Subtype A	Treatment + RSV postF IgG bAb	0.86 (0.49,1.51)	0.601	0.790	0.42 (0.23,0.74)	0.003	0.006
RSV Subtype B	Treatment	0.56 (0.35,0.89)	0.015	0.075			
RSV Subtype B	Treatment + RSV-A nAb	1.13 (0.60,2.15)	0.702	0.702	0.46 (0.27,0.79)	0.004	0.008
RSV Subtype B	Treatment + RSV-B nAb	1.28 (0.66,2.47)	0.469	0.702	0.35 (0.18,0.68)	0.002	0.006
RSV Subtype B	Treatment + RSV preF IgG bAb	1.37 (0.67,2.80)	0.395	0.702	0.36 (0.18,0.72)	0.003	0.006
RSV Subtype B	Treatment + RSV postF IgG bAb	0.87 (0.49,1.54)	0.628	0.702	0.52 (0.29,0.95)	0.034	0.034

*Age, LRTD At-Risk, and baseline risk score are adjusted.

Hazard Ratio (Treatment axis: 0, 0.5, 1, 1.5, 2); Hazard Ratio (Day 29 Marker axis: 0, 0.2, 0.4, 0.6, 0.8, 1)

Fig. S31. Forest plot of the univariable inverse probability of sampling weighted Cox PH regression model summary for evaluating individual Day 29 antibody markers as correlates of risk and protection against each RSV endpoint (RSV-LRTD-2+ [a], RSV-LRTD-3+ [b], and RSV-ARD [c]) caused by RSV subtype A and subtype B, respectively, in the corresponding Day 29 case-cohort set.

In each panel, the rows highlighted in yellow represent the marginal treatment (vaccine) effect, with the below rows representing the conditional treatment effect with a specific Day 29 marker. Hazard ratios for the treatment (vaccine) effect of each model are shown on the left, while hazard ratios for the Day 29 marker effect are shown on the right. Baseline risk factors were adjusted in the univariable inverse probability of sampling weighted Cox regression model, including the actual stratification factors age and LRTD at-risk, and baseline risk score.

ARD, acute respiratory disease; bAb, binding antibody; CI confidence interval; FWER, family-wise error rate; HR, hazard ratio; IgG, immunoglobulin G; LRTD, lower respiratory tract disease; nAb, neutralizing antibody; PH, proportional hazards; postF, postfusion; preF, prefusion; RSV, respiratory syncytial virus; VE, vaccine efficacy.

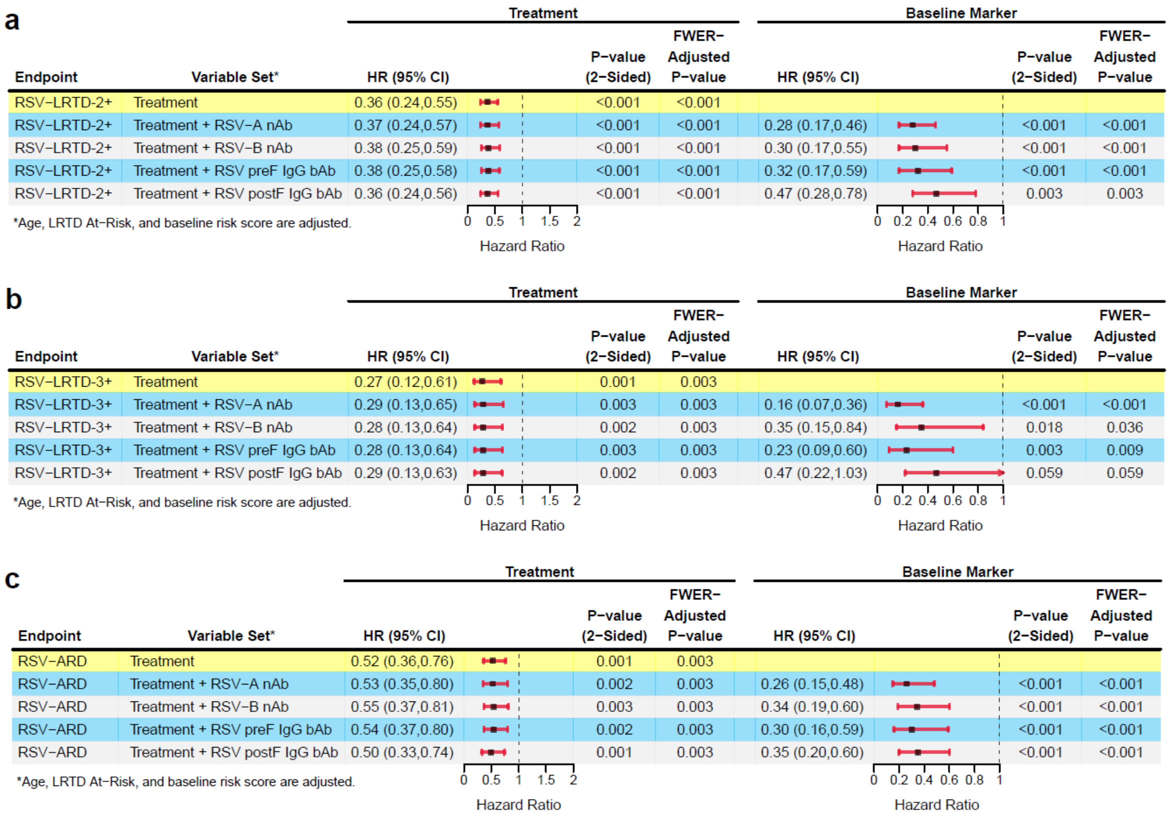

a

Endpoint	Variable Set*	Treatment: HR (95% CI)	Treatment: P-value (2-Sided)	Treatment: FWER-Adjusted P-value	Baseline Marker: HR (95% CI)	Baseline Marker: P-value (2-Sided)	Baseline Marker: FWER-Adjusted P-value
RSV-LRTD-2+	Treatment	0.36 (0.24,0.55)	<0.001	<0.001			
RSV-LRTD-2+	Treatment + RSV-A nAb	0.37 (0.24,0.57)	<0.001	<0.001	0.28 (0.17,0.46)	<0.001	<0.001
RSV-LRTD-2+	Treatment + RSV-B nAb	0.38 (0.25,0.59)	<0.001	<0.001	0.30 (0.17,0.55)	<0.001	<0.001
RSV-LRTD-2+	Treatment + RSV preF IgG bAb	0.38 (0.25,0.58)	<0.001	<0.001	0.32 (0.17,0.59)	<0.001	<0.001
RSV-LRTD-2+	Treatment + RSV postF IgG bAb	0.36 (0.24,0.56)	<0.001	<0.001	0.47 (0.28,0.78)	0.003	0.003

*Age, LRTD At-Risk, and baseline risk score are adjusted.

b

Endpoint	Variable Set*	Treatment: HR (95% CI)	Treatment: P-value (2-Sided)	Treatment: FWER-Adjusted P-value	Baseline Marker: HR (95% CI)	Baseline Marker: P-value (2-Sided)	Baseline Marker: FWER-Adjusted P-value
RSV-LRTD-3+	Treatment	0.27 (0.12,0.61)	0.001	0.003			
RSV-LRTD-3+	Treatment + RSV-A nAb	0.29 (0.13,0.65)	0.003	0.003	0.16 (0.07,0.36)	<0.001	<0.001
RSV-LRTD-3+	Treatment + RSV-B nAb	0.28 (0.13,0.64)	0.002	0.003	0.35 (0.15,0.84)	0.018	0.036
RSV-LRTD-3+	Treatment + RSV preF IgG bAb	0.28 (0.13,0.64)	0.003	0.003	0.23 (0.09,0.60)	0.003	0.009
RSV-LRTD-3+	Treatment + RSV postF IgG bAb	0.29 (0.13,0.63)	0.002	0.003	0.47 (0.22,1.03)	0.059	0.059

*Age, LRTD At-Risk, and baseline risk score are adjusted.

c

Endpoint	Variable Set*	Treatment: HR (95% CI)	Treatment: P-value (2-Sided)	Treatment: FWER-Adjusted P-value	Baseline Marker: HR (95% CI)	Baseline Marker: P-value (2-Sided)	Baseline Marker: FWER-Adjusted P-value
RSV-ARD	Treatment	0.52 (0.36,0.76)	0.001	0.003			
RSV-ARD	Treatment + RSV-A nAb	0.53 (0.35,0.80)	0.002	0.003	0.26 (0.15,0.48)	<0.001	<0.001
RSV-ARD	Treatment + RSV-B nAb	0.55 (0.37,0.81)	0.003	0.003	0.34 (0.19,0.60)	<0.001	<0.001
RSV-ARD	Treatment + RSV preF IgG bAb	0.54 (0.37,0.80)	0.002	0.003	0.30 (0.16,0.59)	<0.001	<0.001
RSV-ARD	Treatment + RSV postF IgG bAb	0.50 (0.33,0.74)	0.001	0.003	0.35 (0.20,0.60)	<0.001	<0.001

*Age, LRTD At-Risk, and baseline risk score are adjusted.

Fig. S32. Forest plot of univariable inverse probability of sampling weighted Cox PH regression model summary for evaluating individual baseline antibody markers as correlates of risk and protection against each RSV endpoint (RSV-LRTD-2+ [a], RSV-LRTD-3+ [b], and RSV-ARD [c]) in the corresponding Day 29 case-cohort set, respectively.

In each panel, the rows highlighted in yellow represent the marginal treatment (vaccine) effect, with the below rows representing the conditional treatment effect with specific Day 29 marker. Rows highlighted in blue are for the top-performance CoRs/CoPs. Hazard ratios for the treatment (vaccine) effect of each model are shown on the left, while hazard ratios for the baseline marker effect are shown on the right. Baseline risk factors were adjusted in the univariable inverse probability of sampling weighted Cox regression model, including the actual stratification factors age and LRTD at-risk, and baseline risk score.

ARD, acute respiratory disease; bAb, binding antibody; CI confidence interval; CoP, correlate of protection; CoR, correlate of risk; FWER, family-wise error rate; HR, hazard ratio; IgG, immunoglobulin G; LRTD, lower respiratory tract disease; nAb, neutralizing antibody; PH, proportional hazards; postF, postfusion; preF, prefusion; RSV, respiratory syncytial virus; VE, vaccine efficacy.

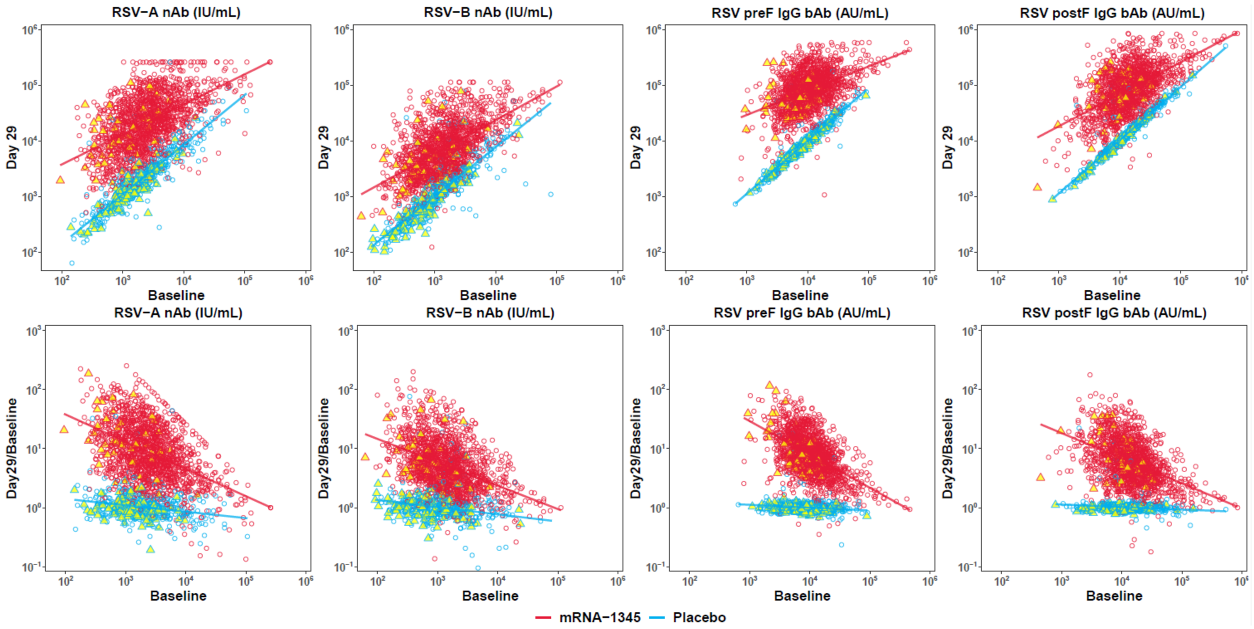

Fig. S33. Scatter plots of baseline vs. Day 29 antibody markers and baseline vs. fold-rise antibody markers in placebo and vaccine recipients in the Day 29 case-cohort set for RSV-LRTD-2+.

Yellow-filled red and blue triangles represent the RSV-LRTD-2+ breakthrough cases, and blue and red circles represent non-cases in vaccine and placebo recipients. For each RSV nAb and IgG bAb marker, the Spearman rank correlation between the fold-rise and baseline marker levels in vaccine recipients was -0.866.

bAb, binding antibody; IgG, immunoglobulin G; LRTD, lower respiratory tract disease; nAb, neutralizing antibody; postF, postfusion; preF, prefusion; RSV, respiratory syncytial virus.

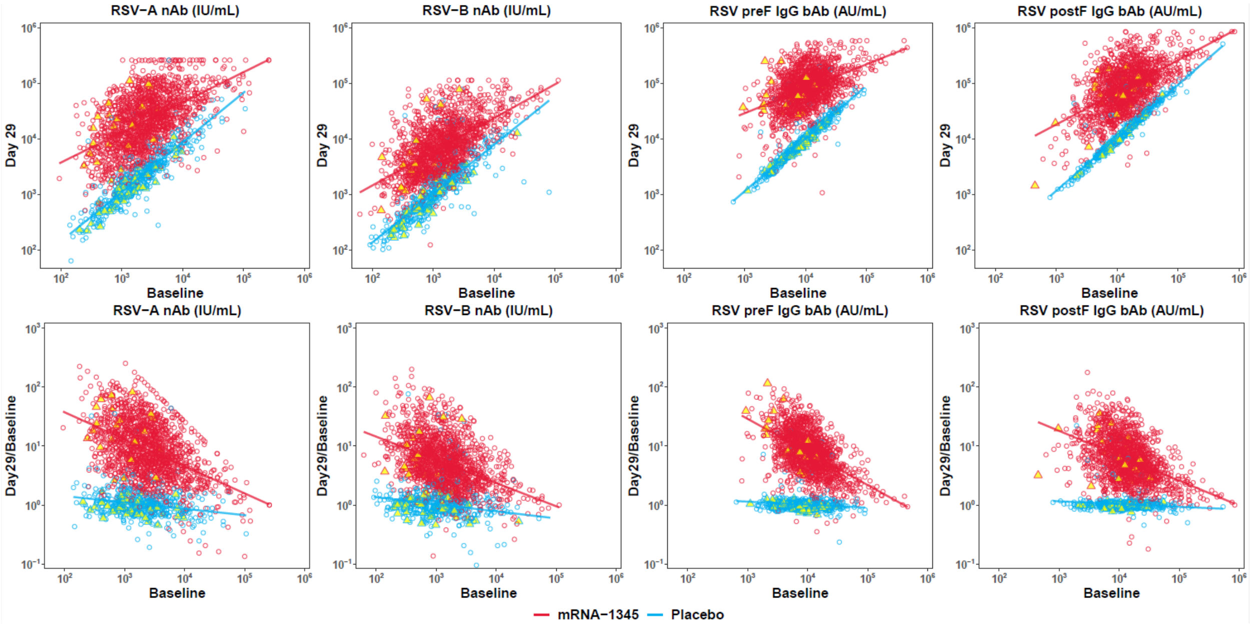

Fig. S34. Scatter plots of baseline vs. Day 29 antibody markers and baseline vs. fold-rise antibody markers in placebo and vaccine recipients in the Day 29 case-cohort set for RSV-LRTD-3+.

Yellow-filled red and blue triangles represent the RSV-LRTD-3+ breakthrough cases, and blue and red circles represent non-cases in vaccine and placebo recipients. For each RSV nAb and IgG bAb marker, the Spearman rank correlation between the fold-rise and baseline marker levels in vaccine recipients was -0.866.

bAb, binding antibody; IgG, immunoglobulin G; LRTD, lower respiratory tract disease; nAb, neutralizing antibody; postF, postfusion; preF, prefusion; RSV, respiratory syncytial virus.

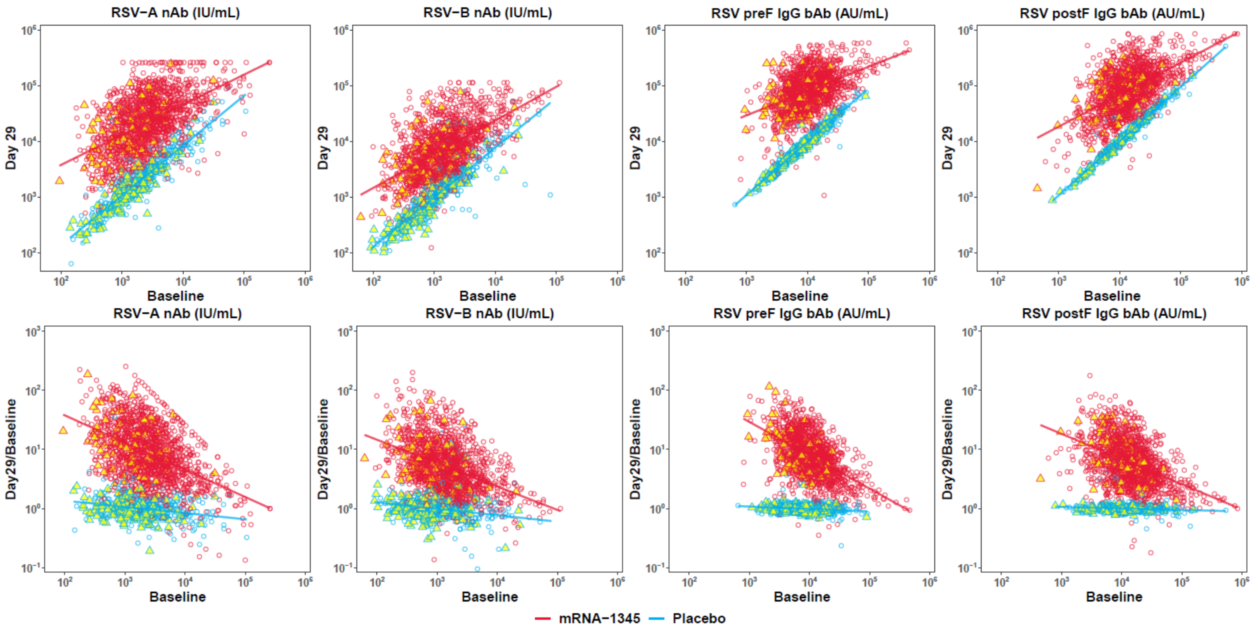

Fig. S35. Scatter plots of baseline vs. Day 29 antibody markers and baseline vs. fold-rise antibody markers in placebo and vaccine recipients in the Day 29 case-cohort set for RSV-ARD.

Yellow-filled red and blue triangles represent the RSV-ARD breakthrough cases, and blue and red circles represent non-cases in vaccine and placebo recipients. For each RSV nAb and IgG bAb marker, the Spearman rank correlation between the fold-rise and baseline marker levels in vaccine recipients was -0.866.

ARD, acute respiratory disease; bAb, binding antibody; IgG, immunoglobulin G; LRTD, lower respiratory tract disease; nAb, neutralizing antibody; postF, postfusion; preF, prefusion; RSV, respiratory syncytial virus.

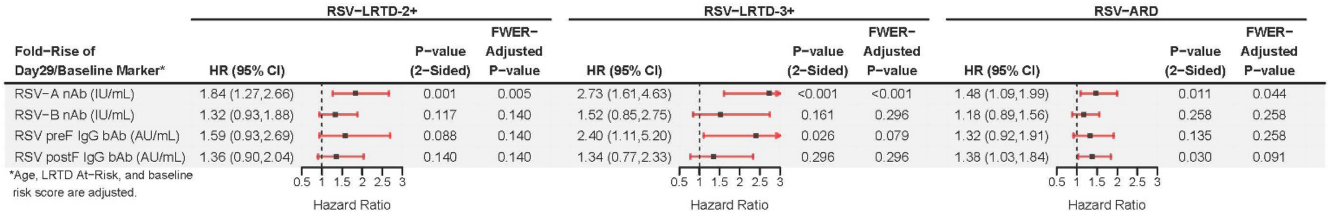

Fold−Rise of Day29/Baseline Marker*	RSV−LRTD-2+ HR (95% CI)	RSV−LRTD-2+ P−value (2−Sided)	RSV−LRTD-2+ FWER−Adjusted P−value	RSV−LRTD-3+ HR (95% CI)	RSV−LRTD-3+ P−value (2−Sided)	RSV−LRTD-3+ FWER−Adjusted P−value	RSV−ARD HR (95% CI)	RSV−ARD P−value (2−Sided)	RSV−ARD FWER−Adjusted P−value
RSV−A nAb (IU/mL)	1.84 (1.27,2.66)	0.001	0.005	2.73 (1.61,4.63)	<0.001	<0.001	1.48 (1.09,1.99)	0.011	0.044
RSV−B nAb (IU/mL)	1.32 (0.93,1.88)	0.117	0.140	1.52 (0.85,2.75)	0.161	0.296	1.18 (0.89,1.56)	0.258	0.258
RSV preF IgG bAb (AU/mL)	1.59 (0.93,2.69)	0.088	0.140	2.40 (1.11,5.20)	0.026	0.079	1.32 (0.92,1.91)	0.135	0.258
RSV postF IgG bAb (AU/mL)	1.36 (0.90,2.04)	0.140	0.140	1.34 (0.77,2.33)	0.296	0.296	1.38 (1.03,1.84)	0.030	0.091

*Age, LRTD At−Risk, and baseline risk score are adjusted.

Fig. S36. Forest plot of univariable inverse probability of sampling weighted Cox PH regression model summary for evaluating individual fold-rise antibody markers as correlates of risk and protection against each RSV endpoint (RSV-LRTD-2+, RSV-LRTD-3+, and RSV-ARD) in vaccine recipients in the corresponding Day 29 case-cohort set.

Baseline risk factors were adjusted in the univariable inverse probability of sampling weighted Cox regression model, including the actual stratification factors age and LRTD at-risk, and baseline risk score.

ARD, acute respiratory disease; bAb, binding antibody; CI, confidence interval; FWER, family-wise error rate; HR, hazard ratio; IgG, immunoglobulin G; LRTD, lower respiratory tract disease; nAb, neutralizing antibody; PH, proportional hazards; postF, postfusion; preF, prefusion; RSV, respiratory syncytial virus.

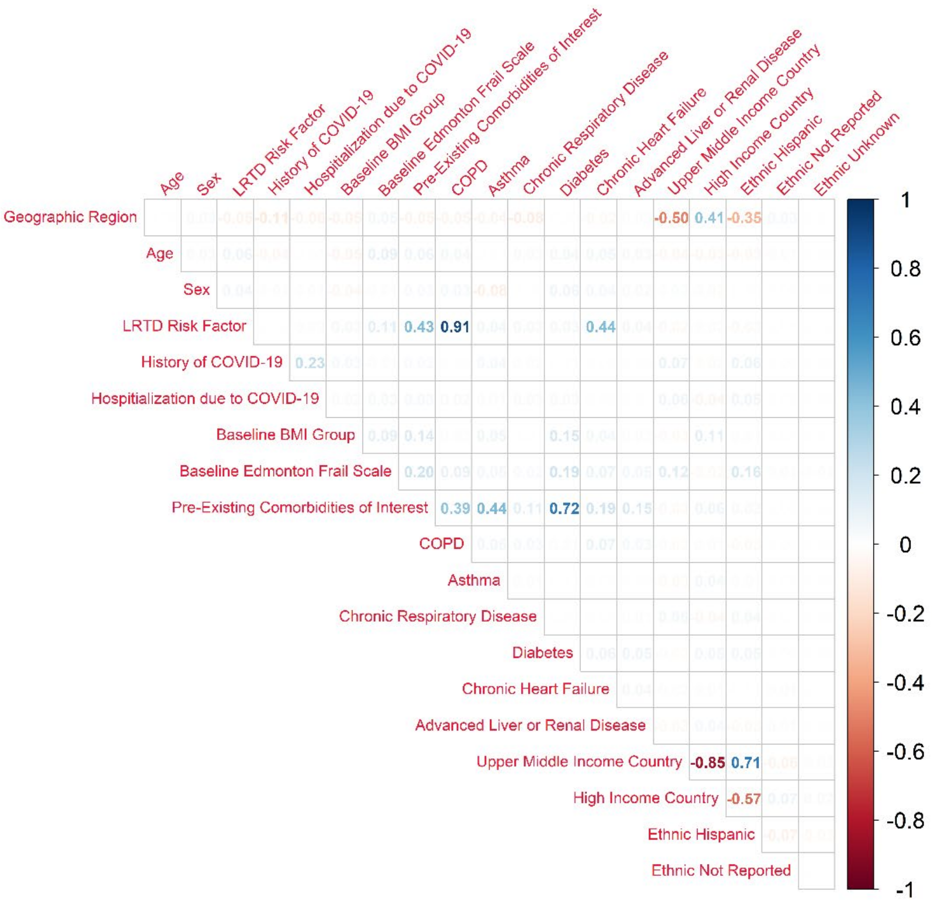

Fig. S37. Correlation of pre-specified baseline covariates in the per-protocol efficacy set for immune correlate analysis (PPESICA) in placebo recipients.

The upper triangle shows the correlations among the baseline covariates using the Spearman method.

BMI, body mass index; COPD, chronic obstructive pulmonary disease; COVID-19, coronavirus disease 2019; LRTD, lower respiratory tract disease.

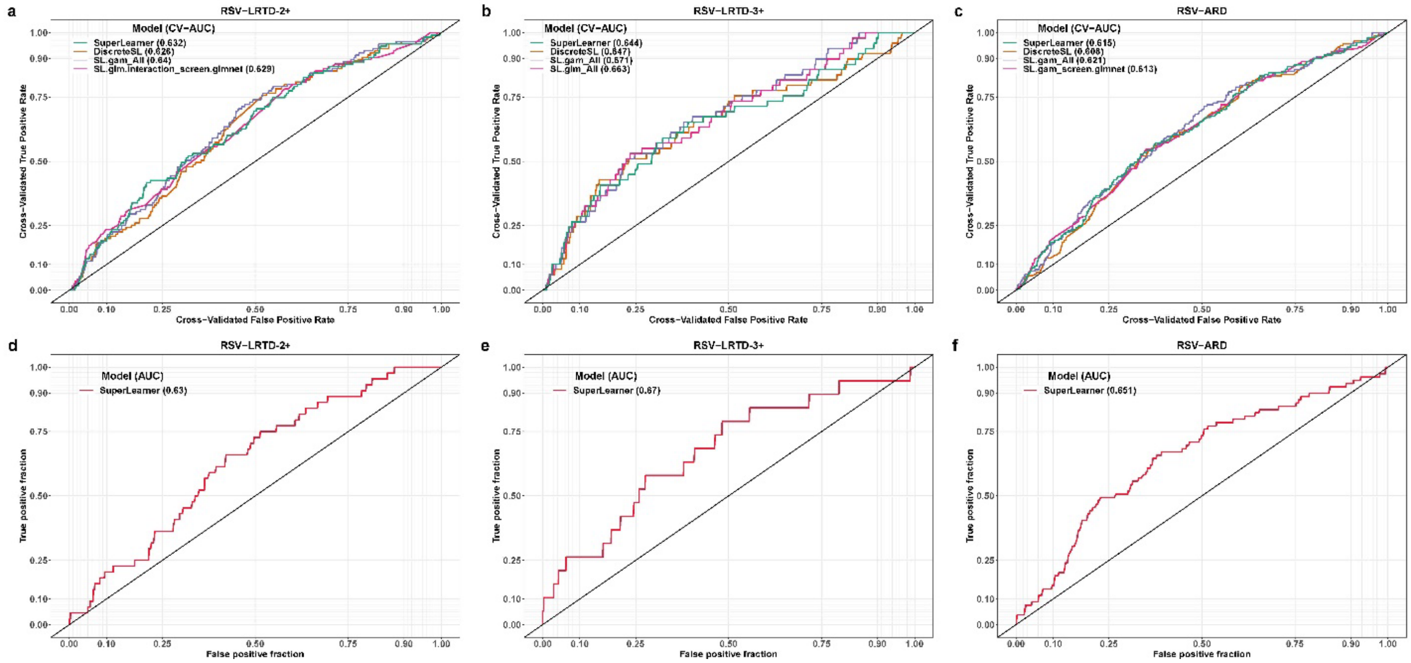

Fig. S38. Receiver operating characteristic curves for placebo and vaccine recipients. (a)-(c) demonstrate the cross-validated receiver operating characteristic curves with the corresponding CV-AUC by Super Learner, Discrete Super Learner, and the two top-performing learners for predicting the occurrence of RSV endpoints (RSV-LRTD-2+, RSV-LRTD-3+, RSV-ARD) using the Super Learner algorithm in placebo recipients in PPESICA. (d)-(f) show the receiver operating characteristic curve with the corresponding AUC by applying the fitted Super Learner model from the placebo recipients to predict the occurrence of each RSV endpoint in vaccine recipients in the PPESICA. CV-AUC and AUC represent the prediction performance, and higher CV-AUC or AUC values mean better predictive power. The range of CV-AUC or AUC is between 0 and 1.

ARD, acute respiratory disease; AUC, area under the curve; CV-AUC, cross-validated area under the curve; LRTD, lower respiratory tract disease; PPESICA, per-protocol efficacy set for immune correlate analysis; RSV, respiratory syncytial virus.

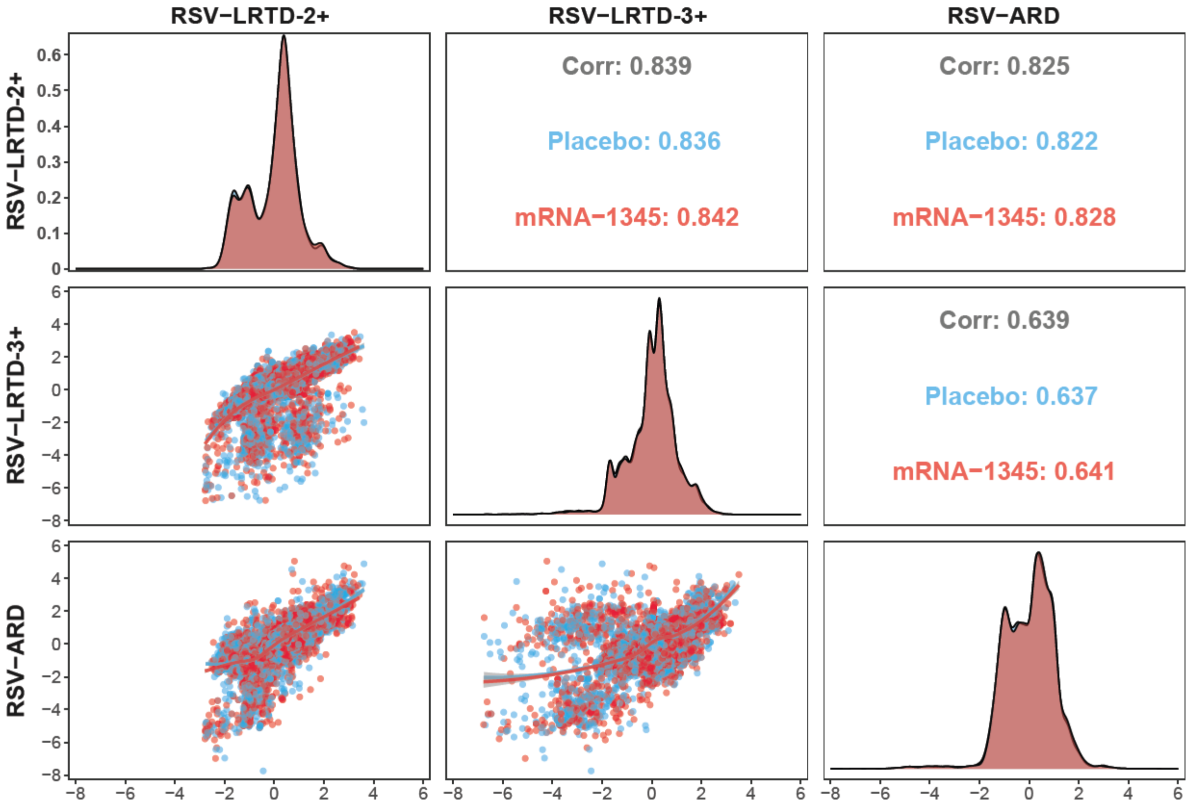

Fig. S39. Correlation of baseline risk scores for RSV endpoints (RSV-LRTD-2+, RSV-LRTD-3+, and RSV-ARD).

Spearman correlations among the three baseline risk scores in placebo recipients (blue), vaccine recipients (red), and combined placebo and vaccine recipients (gray) are calculated and displayed in the upper triangle.

ARD, acute respiratory disease; Corr, correlation; LRTD, lower respiratory tract disease; RSV, respiratory syncytial virus.

Supplementary Tables

Table S1. Sample size of Day 29 case-cohort sets for each RSV endpoint (RSV-LRTD-2+, RSV-LRTD-3+, and RSV-ARD) by the demographic strata and case/non-case strata.

		mRNA-1345								Placebo							
**Endpoint**	**Status**	**S1**	**S2**	**S3**	**S4**	**S5**	**S6**	**S7**	**Total**	**S1**	**S2**	**S3**	**S4**	**S5**	**S6**	**S7**	**Total**
RSV-LRTD-2+	Case	2	0	1	26	5	9	1	44	9	1	3	74	14	13	0	114
	Non-case	213	185	247	239	209	217	206	1,516	44	37	80	87	46	54	37	385
RSV-LRTD-3+	Case	2	0	0	11	3	3	0	19	6	0	1	31	7	4	0	49
	Non-case	213	185	248	257	211	223	207	1,544	48	38	82	136	54	64	37	459
RSV-ARD	Case	4	0	1	52	8	13	1	79	11	1	3	108	17	20	0	160
	Non-case	211	185	247	210	206	213	206	1,478	42	37	79	46	43	44	37	328

Demographic strata: S1. Age 60-74 years, LRTD risk present, Northern Hemisphere; S2. Age 60-74 years, LRTD risk present, Southern Hemisphere; S3. Age ≥75 years, LRTD risk present (merged with regions); S4. Age 60-74 years, LRTD risk absent, Northern Hemisphere; S5. Age 60-74 years, LRTD risk absent, Southern Hemisphere; S6. Age ≥75 years, LRTD risk absent, Northern Hemisphere; S7. Age ≥75 years, LRTD risk absent, Southern Hemisphere. The stratum S3 for the participants with LRTD risk present and age ≥75 years was merged by the geographic region due to the imbalanced sample size in the Northern and Southern Hemisphere regions.

The CCIAS consists of 2,130 participants, and within the CCIAS, the PPICC consists of 2,093 participants by excluding 37 participants either without both Day 1 and Day 29 immunogenicity data or without clinical efficacy data. Day 29 case-cohort set is further derived based on the PPICC by excluding the participants who had the corresponding RSV endpoint by 7 days after the Day 29 visit. Specifically, of the total of 2,093 participants in the PPICC, to conduct correlate analysis for RSV-LRTD-2+, the Day 29 case-cohort set included 2,059 participants by excluding 34 participants who had RSV-LRTD-2+ by 7 days after the Day 29 visit; to conduct correlate analysis for RSV-LRTD-3+, the Day 29 case-cohort set included 2,071 participants by excluding 22 participants who had RSV-LRTD-3+ by 7 days after the Day 29 visit; to conduct correlate analysis for RSV-ARD, the Day 29 case-cohort set included 2,045 participants by excluding 48 participants who had RSV-ARD by 7 days after the Day 29 visit. For each RSV endpoint, case is defined by the first occurrence of the corresponding RSV endpoint more than 7 days after the Day 29 visit to 12 months post-injection, and the non-case refers to no occurrence of such RSV endpoint more than 7 days after Day 29 visit to 12 months post-injection.

ARD, acute respiratory disease; CCIAS, case-cohort immunogenicity analysis set; LRTD, lower respiratory tract disease; PPICC, per-protocol immunogenicity case-cohort set; RSV, respiratory syncytial virus.

Table S2. Number of cases and non-cases specified by each RSV endpoint (RSV-LRTD-2+, RSV-LRTD-3+, and RSV-ARD) in vaccine and placebo recipients in the per-protocol efficacy set for immune correlate analysis (PPESICA) by excluding those who had the corresponding RSV endpoint onset or censored by 7 days after the Day 29 visit.

	No. Cases/No. At-Risk	
Endpoint	mRNA-1345	Placebo*
RSV-LRTD-2+	44/17606	115/17478
RSV-LRTD-3+	19/17609	49/17487
RSV-ARD	79/17602	161/17467

*One placebo recipient had the RSV case onset but did not have the immunologic marker data.

ARD, acute respiratory disease; LRTD, lower respiratory tract disease; RSV, respiratory syncytial virus.

Table S3. Clinical and demographics characteristics of the per-protocol efficacy set for immune correlate analysis (PPESICA) and the per-protocol immunogenicity subcohort set (PPISC).

	Per-Protocol Efficacy Set for Immune Correlate Analysis (PPESICA)*			Per-Protocol Immunogenicity Subcohort Set (PPISC)†		
	**Placebo (N=17528)**	**mRNA-1345 (N=17635)**	**Overall (N=35163)**	**Placebo (N=327)**	**mRNA-1345 (N=1489)**	**Overall (N=1816)**
World Bank Country Income Level 2022						
Lower-middle-income	1206 (6.9%)	1210 (6.9%)	2416 (6.9%)	6 (1.8%)	34 (2.3%)	40 (2.2%)
Upper-middle-income	4633 (26.4%)	4662 (26.4%)	9295 (26.4%)	122 (37.3%)	633 (42.5%)	755 (41.6%)
High-income	11689 (66.7%)	11763 (66.7%)	23452 (66.7%)	199 (60.9%)	822 (55.2%)	1021 (56.2%)
Geographic Region						
Southern Hemisphere	2709 (15.5%)	2724 (15.4%)	5433 (15.5%)	135 (41.3%)	655 (44.0%)	790 (43.5%)
Northern Hemisphere	14819 (84.5%)	14911 (84.6%)	29730 (84.5%)	192 (58.7%)	834 (56.0%)	1026 (56.5%)
Age						
Mean (SD)	68.5 (6.62)	68.5 (6.60)	68.5 (6.61)	72.9 (7.38)	71.9 (7.32)	72.1 (7.34)
Median [Min, Max]	67.0 [60.0, 105]	67.0 [60.0, 108]	67.0 [60.0, 108]	74.0 [60.0, 94.0]	72.0 [60.0, 94.0]	73.0 [60.0, 94.0]
Age Group						
60 TO 74 YEARS	14336 (81.8%)	14440 (81.9%)	28776 (81.8%)	165 (50.5%)	824 (55.3%)	989 (54.5%)
≥75 YEARS	3192 (18.2%)	3195 (18.1%)	6387 (18.2%)	162 (49.5%)	665 (44.7%)	827 (45.5%)
Sex						
Female	8646 (49.3%)	8641 (49.0%)	17287 (49.2%)	147 (45.0%)	670 (45.0%)	817 (45.0%)
Male	8882 (50.7%)	8994 (51.0%)	17876 (50.8%)	180 (55.0%)	819 (55.0%)	999 (55.0%)
Ethnicity						
NOT HISPANIC OR LATINO	11445 (65.3%)	11623 (65.9%)	23068 (65.6%)	182 (55.7%)	777 (52.2%)	959 (52.8%)
HISPANIC OR LATINO	5878 (33.5%)	5832 (33.1%)	11710 (33.3%)	143 (43.7%)	691 (46.4%)	834 (45.9%)
NOT REPORTED	184 (1.0%)	155 (0.9%)	339 (1.0%)	2 (0.6%)	18 (1.2%)	20 (1.1%)
UNKNOWN	21 (0.1%)	25 (0.1%)	46 (0.1%)	0 (0%)	3 (0.2%)	3 (0.2%)
LRTD Risk Factor						
ABSENT	16282 (92.9%)	16380 (92.9%)	32662 (92.9%)	182 (55.7%)	913 (61.3%)	1095 (60.3%)
PRESENT	1246 (7.1%)	1255 (7.1%)	2501 (7.1%)	145 (44.3%)	576 (38.7%)	721 (39.7%)
History of COVID-19						
NO	15770 (90.0%)	15758 (89.4%)	31528 (89.7%)	283 (86.5%)	1289 (86.6%)	1572 (86.6%)
YES	1758 (10.0%)	1877 (10.6%)	3635 (10.3%)	44 (13.5%)	200 (13.4%)	244 (13.4%)
Hospitalization due to COVID-19						
NO	17423 (99.4%)	17525 (99.4%)	34948 (99.4%)	321 (98.2%)	1471 (98.8%)	1792 (98.7%)
YES	105 (0.6%)	110 (0.6%)	215 (0.6%)	6 (1.8%)	18 (1.2%)	24 (1.3%)
Baseline BMI Group						
<30 kg/m^2	12687 (72.4%)	12754 (72.3%)	25441 (72.4%)	231 (70.6%)	1026 (68.9%)	1257 (69.2%)
≥30 kg/m^2	4822 (27.5%)	4868 (27.6%)	9690 (27.6%)	96 (29.4%)	460 (30.9%)	556 (30.6%)
Missing	19 (0.1%)	13 (0.1%)	32 (0.1%)	0 (0%)	3 (0.2%)	3 (0.2%)
Baseline Edmonton Frail Scale						
Mean (SD)	2.29 (1.82)	2.28 (1.81)	2.28 (1.82)	2.69 (2.07)	2.74 (2.02)	2.73 (2.03)
Median [Min, Max]	2.00 [0, 14.0]	2.00 [0, 14.0]	2.00 [0, 14.0]	2.00 [0, 12.0]	2.00 [0, 11.0]	2.00 [0, 12.0]

	Per-Protocol Efficacy Set for Immune Correlate Analysis (PPESICA)*			Per-Protocol Immunogenicity Subcohort Set (PPISC)†		
	**Placebo (N=17528)**	**mRNA-1345 (N=17635)**	**Overall (N=35163)**	**Placebo (N=327)**	**mRNA-1345 (N=1489)**	**Overall (N=1816)**
Missing	886 (5.1%)	877 (5.0%)	1763 (5.0%)	4 (1.2%)	22 (1.5%)	26 (1.4%)
Pre-Existing Comorbidities of Interest						
0	12468 (71.1%)	12425 (70.5%)	24893 (70.8%)	137 (41.9%)	636 (42.7%)	773 (42.6%)
≥1	5060 (28.9%)	5210 (29.5%)	10270 (29.2%)	190 (58.1%)	853 (57.3%)	1043 (57.4%)
COPD						
NO	16479 (94.0%)	16589 (94.1%)	33068 (94.0%)	216 (66.1%)	1020 (68.5%)	1236 (68.1%)
YES	1049 (6.0%)	1046 (5.9%)	2095 (6.0%)	111 (33.9%)	469 (31.5%)	580 (31.9%)
Asthma						
NO	16244 (92.7%)	16279 (92.3%)	32523 (92.5%)	295 (90.2%)	1338 (89.9%)	1633 (89.9%)
YES	1284 (7.3%)	1356 (7.7%)	2640 (7.5%)	32 (9.8%)	151 (10.1%)	183 (10.1%)
Chronic Respiratory Disease						
NO	17448 (99.5%)	17553 (99.5%)	35001 (99.5%)	323 (98.8%)	1469 (98.7%)	1792 (98.7%)
YES	80 (0.5%)	82 (0.5%)	162 (0.5%)	4 (1.2%)	20 (1.3%)	24 (1.3%)
Diabetes						
NO	14492 (82.7%)	14502 (82.2%)	28994 (82.5%)	265 (81.0%)	1162 (78.0%)	1427 (78.6%)
YES	3036 (17.3%)	3133 (17.8%)	6169 (17.5%)	62 (19.0%)	327 (22.0%)	389 (21.4%)
Chronic Heart Failure						
NO	17280 (98.6%)	17376 (98.5%)	34656 (98.6%)	286 (87.5%)	1352 (90.8%)	1638 (90.2%)
YES	248 (1.4%)	259 (1.5%)	507 (1.4%)	41 (12.5%)	137 (9.2%)	178 (9.8%)
Advanced Liver or Renal Disease						
NO	17366 (99.1%)	17489 (99.2%)	34855 (99.1%)	321 (98.2%)	1469 (98.7%)	1790 (98.6%)
YES	162 (0.9%)	146 (0.8%)	308 (0.9%)	6 (1.8%)	20 (1.3%)	26 (1.4%)

*For each RSV endpoint, the placebo recipients in PPESICA who had the RSV endpoint onset or censored more than 7 days after the Day 29 visit were used to build the baseline risk model for predicting the occurrence of the corresponding RSV endpoint using all the listed baseline covariates excluding the age group and COPD. COPD was removed in building the baseline risk model due to the high correlation (Spearman's correlation coefficient >0.9) with the LRTD risk factor. The age group and RSV-LRTD at-risk status were adjusted along with the derived baseline risk score in the correlate analysis.

†There were 1,848 participants selected in the immunogenicity subcohort, of whom 32 participants did not have the efficacy data or did not have the baseline or Day 29 antibody data. Hence, only 1,816 participants were qualified for the per-protocol immunogenicity subcohort set.

BMI, body mass index; COPD, chronic obstructive pulmonary disease; COVID-19, coronavirus disease 2019; LRTD, lower respiratory tract disease; Max, maximum; Min, minimum; RSV, respiratory syncytial virus; SD, standard deviation.

Table S4. Neutralization and IgG binding assay limits of four antibody markers assessed in the immune correlate analysis.

Immunologic Marker	LOD	LLOQ	ULOQ
RSV-A nAb (IU/mL)	NA	13.0	259,061.0
RSV-B nAb (IU/mL)	NA	10.0	112,476.0
RSV preF IgG bAb (AU/mL)	9.8	35.0	580,553.0
RSV postF IgG bAb (AU/mL)	19.5	57.0	847,551.0

bAb, binding antibody; IgG, immunoglobulin G; NA, not applicable; nAb, neutralizing antibody; RSV, respiratory syncytial virus; LLOQ, lower limit of quantification; LOD, limit of detection; postF, postfusion; preF, prefusion; ULOQ, upper limit of quantification.

Table S5. Baseline and Day 29 antibody marker geometric mean titer (GMT) or geometric mean concentration (GMC) by case/non-case strata and by mRNA-1345 50 µg and Placebo in the Day 29 case-cohort set for RSV-LRTD-2+.

Immunologic Marker	Treatment Group or Ratio of GM	Cases GMC or GMT (95% CI)			Non-Cases GMC or GMT (95% CI)			Ratio of GM (Cases/Non-Cases) (95% CI)	
		n	Baseline	Day 29	n	Baseline	Day 29	Baseline	Day 29
RSV-A nAb (IU/mL)	mRNA-1345	44	1014 (757, 1358)	13900 (10216, 18913)	1516	2231 (2040, 2441)	19429 (17655, 21381)	0.5 (0.3, 0.6)	0.7 (0.5, 1.0)
	Placebo	114	1303 (1117, 1520)	1292 (1110, 1504)	385	1950 (1636, 2325)	2061 (1729, 2456)	0.7 (0.5, 0.8)	0.6 (0.5, 0.8)
	Ratio of GM (mRNA-1345/Placebo) (95% CI)		0.8 (0.6, 1.1)	10.8 (7.6, 15.2)		1.1 (0.9, 1.4)	9.4 (7.7, 11.5)		
RSV-B nAb (IU/mL)	mRNA-1345	44	627 (474, 830)	3996 (2883, 5539)	1516	1250 (1143, 1367)	6568 (5993, 7198)	0.5 (0.4, 0.7)	0.6 (0.4, 0.9)
	Placebo	114	716 (596, 860)	733 (606, 887)	385	1091 (941, 1266)	1143 (972, 1343)	0.7 (0.5, 0.8)	0.6 (0.5, 0.8)
	Ratio of GM (mRNA-1345/Placebo) (95% CI)		0.9 (0.6, 1.2)	5.5 (3.7, 8.0)		1.1 (1.0, 1.4)	5.7 (4.8, 6.9)		
RSV preF IgG bAb (AU/mL)	mRNA-1345	44	5827 (4613, 7360)	57822 (46888, 71306)	1516	9509 (8932, 10123)	76735 (71768, 82046)	0.6 (0.5, 0.8)	0.8 (0.6, 0.9)
	Placebo	114	7041 (6153, 8057)	6853 (6024, 7796)	385	8747 (7776, 9839)	9316 (8220, 10558)	0.8 (0.7, 1.0)	0.7 (0.6, 0.9)
	Ratio of GM (mRNA-1345/Placebo) (95% CI)		0.8 (0.6, 1.1)	8.4 (6.6, 10.8)		1.1 (1.0, 1.2)	8.2 (7.1, 9.5)		
RSV postF IgG bAb (AU/mL)	mRNA-1345	44	8652 (6796, 11015)	60649 (44774, 82153)	1516	12885 (11988, 13848)	78222 (72339, 84584)	0.7 (0.5, 0.9)	0.8 (0.6, 1.1)
	Placebo	114	10326 (8887, 11998)	10185 (8758, 11844)	385	12405 (10859, 14170)	13165 (11493, 15080)	0.8 (0.7, 1.0)	0.8 (0.6, 0.9)
	Ratio of GM (mRNA-1345/Placebo) (95% CI)		0.8 (0.6, 1.1)	6.0 (4.2, 8.4)		1.0 (0.9, 1.2)	5.9 (5.1, 6.9)		

bAb, binding antibody; CI, confidence interval; IgG, immunoglobulin G; GM geometric mean; LRTD, lower respiratory tract disease; nAb, neutralizing antibody; postF, postfusion; preF, prefusion; RSV, respiratory syncytial virus.

Table S6. Baseline and Day 29 antibody marker geometric mean titer (GMT) or geometric mean concentration (GMC) by case/non-case strata and by mRNA-1345 50 µg and Placebo in the Day 29 case-cohort set for RSV-LRTD-3+.

Immunologic Marker	Treatment Group or Ratio of GM	Cases GMC or GMT (95% CI)			Non-Cases GMC or GMT (95% CI)			Ratio of GM (Cases/Non-Cases) (95% CI)	
		n	Baseline	Day 29	n	Baseline	Day 29	Baseline	Day 29
RSV-A nAb (IU/mL)	mRNA-1345	19	887 (593, 1327)	18279 (11383, 29351)	1544	2153 (1972, 2351)	18838 (17158, 20683)	0.4 (0.3, 0.6)	1.0 (0.6, 1.6)
	Placebo	49	1279 (1009, 1621)	1249 (992, 1572)	459	1771 (1548, 2025)	1862 (1627, 2131)	0.7 (0.5, 0.9)	0.7 (0.5, 0.9)
	Ratio of GM (mRNA-1345/Placebo) (95% CI)		0.7 (0.4, 1.1)	14.6 (8.6, 24.8)		1.2 (1.0, 1.4)	10.1 (8.6, 11.9)		
RSV-B nAb (IU/mL)	mRNA-1345	19	524 (364, 755)	3903 (2092, 7281)	1544	1208 (1107, 1317)	6366 (5826, 6956)	0.4 (0.3, 0.6)	0.6 (0.3, 1.2)
	Placebo	49	821 (633, 1063)	791 (612, 1022)	459	987 (872, 1118)	1059 (924, 1213)	0.8 (0.6, 1.1)	0.7 (0.6, 1.0)
	Ratio of GM (mRNA-1345/Placebo) (95% CI)		0.6 (0.4, 1.0)	4.9 (2.5, 9.7)		1.2 (1.1, 1.4)	6.0 (5.1, 7.1)		
RSV preF IgG bAb (AU/mL)	mRNA-1345	19	4614 (3215, 6622)	61363 (45247, 83217)	1544	9292 (8741, 9877)	75005 (70234, 80099)	0.5 (0.3, 0.7)	0.8 (0.6, 1.1)
	Placebo	49	7555 (6196, 9213)	7231 (6014, 8694)	459	8164 (7427, 8973)	8671 (7844, 9586)	0.9 (0.7, 1.2)	0.8 (0.7, 1.0)
	Ratio of GM (mRNA-1345/Placebo) (95% CI)		0.6 (0.4, 0.9)	8.5 (5.9, 12.1)		1.1 (1.0, 1.3)	8.6 (7.7, 9.8)		
RSV postF IgG bAb (AU/mL)	mRNA-1345	19	7358 (4540, 11923)	54164 (31540, 93016)	1544	12624 (11777, 13532)	77136 (71450, 83273)	0.6 (0.4, 0.9)	0.7 (0.4, 1.2)
	Placebo	49	11649 (9696, 13996)	11370 (9420, 13722)	459	11443 (10268, 12753)	12117 (10847, 13536)	1.0 (0.8, 1.3)	0.9 (0.8, 1.2)
	Ratio of GM (mRNA-1345/Placebo) (95% CI)		0.6 (0.4, 1.1)	4.8 (2.7, 8.4)		1.1 (1.0, 1.3)	6.4 (5.6, 7.3)		

bAb, binding antibody; CI, confidence interval; IgG, immunoglobulin G; GM geometric mean; LRTD, lower respiratory tract disease; nAb, neutralizing antibody; postF, postfusion; preF, prefusion; RSV, respiratory syncytial virus.

Table S7. Baseline and Day 29 antibody marker geometric mean titer (GMT) or geometric mean concentration (GMC) by case/non-case strata and by mRNA-1345 50 µg and Placebo in the Day 29 case-cohort set for RSV-ARD.

Immunologic Marker	Treatment Group or Ratio of GM	Cases GMC or GMT (95% CI)			Non-Cases GMC or GMT (95% CI)			Ratio of GM (Cases/Non-Cases) (95% CI)	
		n	Baseline	Day 29	n	Baseline	Day 29	Baseline	Day 29
RSV-A nAb (IU/mL)	mRNA-1345	79	1174 (940, 1467)	13952 (10968, 17748)	1478	2285 (2079, 2511)	19817 (17907, 21931)	0.5 (0.4, 0.7)	0.7 (0.5, 0.9)
	Placebo	160	1324 (1141, 1535)	1307 (1129, 1513)	328	2278 (1806, 2873)	2206 (1780, 2734)	0.6 (0.4, 0.8)	0.6 (0.5, 0.8)
	Ratio of GM (mRNA-1345/Placebo) (95% CI)		0.9 (0.7, 1.2)	10.7 (8.1, 14.1)		1.0 (0.8, 1.3)	9.0 (7.1, 11.4)		
RSV-B nAb (IU/mL)	mRNA-1345	79	730 (592, 899)	4288 (3396, 5414)	1478	1272 (1156, 1400)	6755 (6125, 7450)	0.6 (0.5, 0.7)	0.6 (0.5, 0.8)
	Placebo	160	759 (651, 885)	768 (652, 905)	328	1214 (993, 1482)	1177 (965, 1435)	0.6 (0.5, 0.8)	0.7 (0.5, 0.8)
	Ratio of GM (mRNA-1345/Placebo) (95% CI)		1.0 (0.7, 1.2)	5.6 (4.2, 7.4)		1.0 (0.8, 1.3)	5.7 (4.6, 7.2)		
RSV preF IgG bAb (AU/mL)	mRNA-1345	79	6534 (5497, 7767)	60797 (51806, 71349)	1478	9666 (9051, 10323)	77829 (72430, 83630)	0.7 (0.6, 0.8)	0.8 (0.7, 0.9)
	Placebo	160	6981 (6221, 7833)	6860 (6128, 7680)	328	9663 (8262, 11302)	9847 (8438, 11490)	0.7 (0.6, 0.9)	0.7 (0.6, 0.8)
	Ratio of GM (mRNA-1345/Placebo) (95% CI)		0.9 (0.8, 1.2)	8.9 (7.3, 10.8)		1.0 (0.8, 1.2)	7.9 (6.7, 9.4)		
RSV postF IgG bAb (AU/mL)	mRNA-1345	79	9338 (7785, 11202)	66578 (54346, 81563)	1478	13151 (12184, 14196)	78699 (72326, 85634)	0.7 (0.6, 0.9)	0.8 (0.7, 1.1)
	Placebo	160	10144 (8912, 11548)	10083 (8854, 11482)	328	14702 (12384, 17454)	14779 (12431, 17571)	0.7 (0.6, 0.9)	0.7 (0.5, 0.8)
	Ratio of GM (mRNA-1345/Placebo) (95% CI)		0.9 (0.7, 1.2)	6.6 (5.2, 8.4)		0.9 (0.7, 1.1)	5.3 (4.4, 6.5)		

bAb, binding antibody; ARD, acute respiratory disease; CI, confidence interval; IgG, immunoglobulin G; GM geometric mean; nAb, neutralizing antibody; postF, postfusion; preF, prefusion; RSV, respiratory syncytial virus.

Table S8. Covariate-adjusted HRs of each RSV endpoint per 10-fold increase in each Day 29 antibody marker in vaccine and placebo recipients in Day 29 case-cohort set. Baseline risk factors were adjusted in the univariable inverse probability of sampling weighted Cox regression model, including the actual stratification factors age and LRTD at-risk, and baseline risk score.

Endpoint	Immunologic Marker	No. Cases/ No. At-Risk*	Attack Rate	HR per 10-Fold Increase Point Est. (95% CI)	P-Value (2-sided)	FWER Adjusted P-Value†
RSV-LRTD-2+	RSV-A nAb (IU/mL)	44/17555	0.0025	0.44 (0.30,0.65)	<0.001	<0.001
	RSV-B nAb (IU/mL)	44/17553	0.0025	0.42 (0.25,0.72)	0.001	0.002
	RSV preF IgG bAb (AU/mL)	44/17600	0.0025	0.40 (0.23,0.68)	0.001	0.002
	RSV postF IgG bAb (AU/mL)	45/17600	0.0026	0.55 (0.33,0.89)	0.016	0.016
	Placebo	115/17470	0.0066			
RSV-LRTD-3+	RSV-A nAb (IU/mL)	19/17594	0.0011	0.41 (0.20,0.84)	0.014	0.056
	RSV-B nAb (IU/mL)	19/17593	0.0011	0.55 (0.25,1.24)	0.151	0.183
	RSV preF IgG bAb (AU/mL)	19/17637	0.0011	0.47 (0.21,1.04)	0.063	0.183
	RSV postF IgG bAb (AU/mL)	19/17637	0.0011	0.60 (0.28,1.28)	0.183	0.183
	Placebo	51/17395	0.0029			
RSV-ARD	RSV-A nAb (IU/mL)	79/17601	0.0045	0.45 (0.28,0.71)	0.001	0.003
	RSV-B nAb (IU/mL)	80/17546	0.0046	0.42 (0.25,0.73)	0.002	0.003
	RSV preF IgG bAb (AU/mL)	80/17600	0.0045	0.36 (0.21,0.63)	<0.001	<0.001
	RSV postF IgG bAb (AU/mL)	80/17600	0.0045	0.46 (0.28,0.77)	0.003	0.003
	Placebo	162/17463	0.0093			

Age, LRTD at-risk, and baseline risk scores were adjusted in the Cox PH model. The maximum failure event time after the Day 29 visit is 345 days.

*No. of cases: Estimated number of participants receiving vaccine or placebo with RSV endpoint (RSV-LRTD-2+, RSV-LRTD-3+, and RSV-ARD) onset during the study period. No. at-risk: Estimated number of participants receiving vaccine or placebo not experiencing RSV endpoint (RSV-LRTD-2+, RSV-LRTD-3+, and RSV-ARD) onset by 7 days after the Day 29 visit. The estimated No. of cases and No. at-risk are slightly different due to the variability of the number of participants with observed eligible antibody marker data.

†FWER-adjusted P-values (two-sided) are calculated by each RSV endpoint using the Hommel method.

ARD, acute respiratory disease; bAb, binding antibody; Est, estimate; FWER, family-wise error rate; HR, hazard ratio; LRTD, lower respiratory tract disease; nAb, neutralizing antibody; PH, proportional hazards; postF, postfusion; preF, prefusion; RSV, respiratory syncytial virus.

Table S9. Interaction analysis between each antibody marker (RSV-A nAb, RSV-B nAb, RSV preF IgG bAb, RSV postF IgG bAb) and the treatment in vaccine and placebo recipients in the Day 29 case-cohort set for each RSV endpoint (RSV-LRTD-2+, RSV-LRTD-3+, and RSV-ARD).

Univariable inverse probability of sampling weighted Cox PH regression model was applied to fit each RSV endpoint by the treatment, an individual Day 29 antibody marker, and the interaction of treatment and the individual Day 29 antibody marker, adjusted by the baseline risk factors including the actual stratification factors age and RSV-LRTD at-risk, and baseline risk score. FWER-adjusted P-value (two-sided) is calculated by Hommel's method.

	RSV-LRTD-2+		RSV-LRTD-3+		RSV-ARD	
**Interaction Term**	**P-Value (2-sided)**	**FWER Adjusted P-Value**	**P-Value (2-sided)**	**FWER Adjusted P-Value**	**P-Value (2-sided)**	**FWER Adjusted P-Value**
Treatment*RSV-A nAb	0.222	0.688	0.045	0.179	0.301	0.602
Treatment*RSV-B nAb	0.688	0.688	0.778	0.778	0.839	0.839
Treatment*RSV preF IgG bAb	0.474	0.688	0.312	0.778	0.286	0.573
Treatment*RSV postF IgG bAb	0.550	0.688	0.540	0.778	0.059	0.238

ARD, acute respiratory disease; bAb, binding antibody; FWER, family-wise error rate; IgG, immunoglobulin G; LRTD, lower respiratory tract disease; nAb, neutralizing antibody; PH, proportional hazards; postF, postfusion; preF, prefusion; RSV, respiratory syncytial virus.

Table S10. Covariate-adjusted HRs of each RSV endpoint in low, medium, and high tertiles of vaccine recipients by each Day 29 antibody marker compared with the placebo recipients in the Day 29 case-cohort set.

Baseline risk factors were adjusted in the univariable (qualitative) inverse probability of sampling weighted Cox regression model, including the actual stratification factors age and LRTD at-risk, and baseline risk score.

Endpoint	Immunologic Marker	Tertile	No. Cases/ No. At-Risk*	Attack Rate	HR Point Est. (95% CI)	P-Value (2-sided)	FWER Adjusted P-Value†
RSV-LRTD-2+	RSV-A nAb (IU/mL)	Low (< 11640 AU/mL)	19/5852	0.0032	0.44 (0.25,0.77)	0.004	0.012
		Med (11640 to 32015 AU/mL)	15/5860	0.0026	0.37 (0.20,0.69)	0.002	0.008
		High (> 32015 AU/mL)	10/5842	0.0017	0.27 (0.13,0.54)	<0.001	<0.001
	RSV-B nAb (IU/mL)	Low (< 3833 AU/mL)	23/5849	0.0039	0.49 (0.29,0.86)	0.012	0.012
		Med (3833 to 10371 AU/mL)	15/5858	0.0026	0.37 (0.20,0.68)	0.001	0.006
		High (> 10371 AU/mL)	6/5846	0.0010	0.17 (0.07,0.41)	<0.001	<0.001
	RSV preF IgG bAb (AU/mL)	Low (< 57656 AU/mL)	22/5881	0.0037	0.48 (0.28,0.83)	0.008	0.012
		Med (57656 to 109395 AU/mL)	15/5860	0.0026	0.37 (0.20,0.69)	0.002	0.008
		High (> 109395 AU/mL)	7/5860	0.0012	0.20 (0.09,0.45)	<0.001	<0.001
	RSV postF IgG bAb (AU/mL)	Low (< 57339 AU/mL)	16/5870	0.0027	0.35 (0.19,0.65)	0.001	0.006
		Med (57339 to 120009 AU/mL)	15/5870	0.0026	0.40 (0.22,0.73)	0.003	0.009
		High (> 120009 AU/mL)	13/5861	0.0022	0.34 (0.18,0.64)	0.001	0.006
	Placebo		116/17474	0.0066			
RSV-LRTD-3+	RSV-A nAb (IU/mL)	Low (< 11320 AU/mL)	7/5853	0.0012	0.27 (0.08,0.86)	0.027	0.082
		Med (11320 to 30827 AU/mL)	6/5875	0.0010	0.25 (0.08,0.76)	0.014	0.068
		High (> 30827 AU/mL)	6/5867	0.0010	0.31 (0.11,0.83)	0.020	0.080
	RSV-B nAb (IU/mL)	Low (< 3671 AU/mL)	12/5869	0.0020	0.46 (0.18,1.18)	0.107	0.107
		Med (3671 to 9871 AU/mL)	4/5856	0.0007	0.16 (0.04,0.58)	0.005	0.040
		High (> 9871 AU/mL)	3/5868	0.0005	0.16 (0.04,0.60)	0.006	0.043
	RSV preF IgG bAb (AU/mL)	Low (< 56876 AU/mL)	9/5875	0.0015	0.31 (0.10,0.95)	0.041	0.107
		Med (56876 to 108128 AU/mL)	7/5862	0.0012	0.31 (0.11,0.87)	0.026	0.082
		High (> 108128 AU/mL)	3/5900	0.0005	0.17 (0.05,0.59)	0.005	0.040
	RSV postF IgG bAb (AU/mL)	Low (< 57334 AU/mL)	6/5848	0.0010	0.21 (0.06,0.70)	0.011	0.055
		Med (57334 to 120383 AU/mL)	8/5879	0.0014	0.47 (0.20,1.12)	0.089	0.107
		High (> 120383 AU/mL)	5/5910	0.0008	0.20 (0.06,0.67)	0.009	0.054
	Placebo		51/17482	0.0029			
RSV-ARD	RSV-A nAb (IU/mL)	Low (< 12079 AU/mL)	39/5860	0.0067	0.77 (0.49,1.24)	0.283	0.283
		Med (12079 to 33296 AU/mL)	23/5875	0.0039	0.41 (0.24,0.72)	0.002	0.016
		High (> 33296 AU/mL)	16/5866	0.0027	0.36 (0.20,0.64)	0.001	0.010
	RSV-B nAb (IU/mL)	Low (< 3957 AU/mL)	38/5874	0.0065	0.71 (0.44,1.13)	0.151	0.283
		Med (3957 to 10596 AU/mL)	28/5776	0.0048	0.57 (0.34,0.94)	0.029	0.145
		High (> 10596 AU/mL)	13/5896	0.0022	0.27 (0.14,0.52)	<0.001	<0.001
	RSV preF IgG bAb (AU/mL)	Low (< 58294 AU/mL)	39/5869	0.0066	0.73 (0.46,1.16)	0.179	0.283

Endpoint	Immunologic Marker	Tertile	No. Cases/ No. At-Risk*	Attack Rate	HR Point Est. (95% CI)	P-Value (2-sided)	FWER Adjusted P-Value†
		Med (58294 to 110821 AU/mL)	26/5878	0.0044	0.52 (0.30,0.88)	0.015	0.087
		High (> 110821 AU/mL)	14/5852	0.0024	0.30 (0.16,0.56)	<0.001	<0.001
	RSV postF IgG bAb (AU/mL)	Low (< 57339 AU/mL)	27/5901	0.0046	0.49 (0.29,0.83)	0.008	0.052
		Med (57339 to 121946 AU/mL)	31/5839	0.0053	0.65 (0.40,1.07)	0.091	0.268
		High (> 121946 AU/mL)	21/5860	0.0036	0.43 (0.24,0.75)	0.003	0.024
	Placebo		164/17467	0.0094			

Age, LRTD at-risk, and baseline risk scores were adjusted in the Cox PH model. The maximum failure event time after the Day 29 visit is 345 days.

*No. of cases: Estimated number of participants receiving vaccine or placebo with RSV endpoint (RSV-LRTD-2+, RSV-LRTD-3+, and RSV-ARD) onset during the study period. No. at-risk: Estimated number of participants receiving vaccine or placebo not experiencing RSV endpoint (RSV-LRTD-2+, RSV-LRTD-3+, and RSV-ARD) onset by 7 days after the Day 29 visit. The estimated no. of cases and no. at-risk are slightly different due to the variability of the number of participants with observed eligible antibody marker data.

†FWER-adjusted P-values (two-sided) are calculated by each RSV endpoint using the Hommel method.

ARD, acute respiratory disease; bAb, binding antibody; FWER, family-wise error rate; HR, hazard ratio; IgG, immunoglobulin G; LRTD, lower respiratory tract disease; Med, medium; nAb, neutralizing antibody; PH, proportional hazards; postF, postfusion; preF, prefusion; RSV, respiratory syncytial virus.

Table S11. Covariate-adjusted HRs of each RSV endpoint caused by RSV subtypes A and B per 10-fold increase in each Day 29 antibody marker in vaccine and placebo recipients in the Day 29 case-cohort set.

Baseline risk factors were adjusted in the univariable inverse probability of sampling weighted Cox regression model, including the actual stratification factors age and LRTD at-risk, and baseline risk score.

Endpoint	Immunologic Marker	No. Cases/ No. At-Risk*	Attack Rate	Hazard Ratio per 10-Fold Increase Point Est. (95% CI)	P-Value (2-sided)	FWER Adjusted P-Value†
RSV-LRTD-2+ RSV Subtype A	RSV-A nAb (IU/mL)	22/17555	0.0013	0.39 (0.24,0.65)	<0.001	<0.001
	RSV-B nAb (IU/mL)	22/17553	0.0013	0.56 (0.29,1.11)	0.096	0.096
	RSV preF IgG bAb (AU/mL)	22/17600	0.0013	0.36 (0.18,0.69)	0.002	0.006
	RSV postF IgG bAb (AU/mL)	22/17600	0.0013	0.44 (0.25,0.80)	0.007	0.014
	Placebo	68/17470	0.0039			
RSV-LRTD-2+ RSV Subtype B	RSV-A nAb (IU/mL)	21/17555	0.0012	0.48 (0.30,0.75)	0.001	0.003
	RSV-B nAb (IU/mL)	21/17553	0.0012	0.28 (0.14,0.57)	<0.001	<0.001
	RSV preF IgG bAb (AU/mL)	21/17600	0.0012	0.42 (0.22,0.79)	0.007	0.014
	RSV postF IgG bAb (AU/mL)	21/17600	0.0012	0.71 (0.38,1.34)	0.292	0.292
	Placebo	48/17470	0.0027			
RSV-LRTD-3+ RSV Subtype A	RSV-A nAb (IU/mL)	10/17594	0.0006	0.26 (0.09,0.71)	0.009	0.036
	RSV-B nAb (IU/mL)	10/17593	0.0006	0.68 (0.22,2.14)	0.510	0.510
	RSV preF IgG bAb (AU/mL)	10/17637	0.0006	0.38 (0.14,1.06)	0.065	0.195
	RSV postF IgG bAb (AU/mL)	10/17637	0.0006	0.64 (0.27,1.53)	0.314	0.510
	Placebo	29/17395	0.0017			
RSV-LRTD-3+ RSV Subtype B	RSV-A nAb (IU/mL)	9/17594	0.0005	0.64 (0.30,1.37)	0.250	0.333
	RSV-B nAb (IU/mL)	9/17593	0.0005	0.44 (0.17,1.16)	0.099	0.333
	RSV preF IgG bAb (AU/mL)	9/17637	0.0005	0.53 (0.20,1.45)	0.217	0.333
	RSV postF IgG bAb (AU/mL)	9/17637	0.0005	0.60 (0.21,1.70)	0.333	0.333
	Placebo	23/17395	0.0013			
RSV-ARD RSV Subtype A	RSV-A nAb (IU/mL)	40/17601	0.0023	0.42 (0.24,0.72)	0.002	0.004
	RSV-B nAb (IU/mL)	40/17546	0.0023	0.50 (0.26,0.94)	0.031	0.031
	RSV preF IgG bAb (AU/mL)	40/17600	0.0023	0.34 (0.18,0.65)	0.001	0.004
	RSV postF IgG bAb (AU/mL)	40/17600	0.0023	0.42 (0.23,0.74)	0.003	0.006
	Placebo	91/17463	0.0052			
RSV-ARD RSV Subtype B	RSV-A nAb (IU/mL)	38/17601	0.0022	0.46 (0.27,0.79)	0.004	0.008
	RSV-B nAb (IU/mL)	39/17546	0.0022	0.35 (0.18,0.68)	0.002	0.006
	RSV preF IgG bAb (AU/mL)	39/17600	0.0022	0.36 (0.18,0.72)	0.003	0.006
	RSV postF IgG bAb (AU/mL)	39/17600	0.0022	0.52 (0.29,0.95)	0.034	0.034
	Placebo	72/17463	0.0041			

Age, LRTD at-risk, and baseline risk scores were adjusted in the Cox PH model. The maximum failure event time after the Day 29 visit is 345 days.
*No. of cases: Estimated number of participants receiving vaccine or placebo with RSV endpoint (RSV-LRTD-2+, RSV-LRTD-3+, and RSV-ARD) onset during the study period . No. at-risk: Estimated number of participants receiving vaccine or placebo not experiencing RSV endpoint (RSV-LRTD-2+, RSV-LRTD-3+, and RSV-ARD) onset by 7 days after the Day 29 visit. The estimated No. of cases and No. at-risk are slightly different due to the variability of the number of participants with observed eligible antibody marker data.

[†]FWER-adjusted p-values (two-sided) are calculated by each RSV endpoint using the Hommel method.

ARD, acute respiratory disease; bAb, binding antibody; Est, estimate; FWER, family-wise error rate; HR, hazard ratio; IgG, immunoglobulin G; LRTD, lower respiratory tract disease; Med, medium; nAb, neutralizing antibody; PH, proportional hazards; postF, postfusion; preF, prefusion; RSV, respiratory syncytial virus.

Table S12. Fold-rise antibody marker geometric means by case/non-case strata and by vaccine and placebo in the Day 29 case-cohort set for RSV-LRTD-2+.

		Cases Fold-Change (95% CI)		Non-Cases Fold-Change (95% CI)		Ratio of Fold-Change (Cases/Non-Cases) (95% CI)
**Immunologic Marker**	**Treatment Group or Ratio of GM**	**n**	**mean (95% CI)**	**n**	**mean (95% CI)**	**FC (95% CI)**
RSV-A nAb (IU/mL)	mRNA-1345	44	13.3 (9.8, 18.2)	1516	8.6 (7.8, 9.5)	1.5 (1.1, 2.1)
	Placebo	114	1.0 (0.9, 1.0)	385	1.1 (1.0, 1.2)	0.9 (0.8, 1.0)
	Ratio of Fold-Change (mRNA-1345/Placebo) (95% CI)		13.6 (9.9, 18.7)		8.2 (7.2, 9.3)	
RSV-B nAb (IU/mL)	mRNA-1345	44	6.1 (4.6, 8.1)	1516	5.2 (4.8, 5.7)	1.2 (0.9, 1.6)
	Placebo	114	1.0 (0.9, 1.1)	385	1.0 (0.9, 1.2)	1.0 (0.9, 1.1)
	Ratio of Fold-Change (mRNA-1345/Placebo) (95% CI)		6.0 (4.4, 8.1)		5.0 (4.4, 5.7)	
RSV preF IgG bAb (AU/mL)	mRNA-1345	44	9.9 (7.5, 13.2)	1516	8.1 (7.5, 8.7)	1.2 (0.9, 1.7)
	Placebo	114	1.0 (0.9, 1.0)	385	1.1 (1.0, 1.1)	0.9 (0.9, 1.0)
	Ratio of Fold-Change (mRNA-1345/Placebo) (95% CI)		10.2 (7.7, 13.6)		7.6 (6.9, 8.3)	
RSV postF IgG bAb (AU/mL)	mRNA-1345	44	7.0 (5.3, 9.3)	1516	6.1 (5.6, 6.5)	1.2 (0.9, 1.5)
	Placebo	114	1.0 (1.0, 1.0)	385	1.1 (1.0, 1.1)	0.9 (0.9, 1.0)
	Ratio of Fold-Change (mRNA-1345/Placebo) (95% CI)		7.1 (5.4, 9.4)		5.7 (5.2, 6.3)	

bAb, binding antibody; CI, confidence interval; FC, fold-change; GM, geometric mean; IgG, immunoglobulin G; LRTD, lower respiratory tract disease; nAb, neutralizing antibody; postF, postfusion; preF, prefusion; RSV, respiratory syncytial virus.

Table S13. Fold-rise antibody marker geometric means by case/non-case strata and by vaccine and placebo in the Day 29 case-cohort set for RSV-LRTD-3+.

Immunologic Marker	Treatment Group or Ratio of GM	Cases Fold-Change (95% CI)		Non-Cases Fold-Change (95% CI)		Ratio of Fold-Change (Cases/Non-Cases) (95% CI)
		n	mean (95% CI)	n	mean (95% CI)	FC (95% CI)
RSV-A nAb (IU/mL)	mRNA-1345	19	20.6 (13.9, 30.6)	1544	8.7 (7.9, 9.5)	2.4 (1.6, 3.6)
	Placebo	49	1.0 (0.9, 1.1)	459	1.0 (1.0, 1.1)	0.9 (0.8, 1.1)
	Ratio of Fold-Change (mRNA-1345/Placebo) (95% CI)		21.1 (14.0, 31.8)		8.3 (7.4, 9.3)	
RSV-B nAb (IU/mL)	mRNA-1345	19	6.6 (3.9, 11.3)	1544	5.2 (4.8, 5.7)	1.3 (0.7, 2.2)
	Placebo	49	1.0 (0.9, 1.1)	459	1.1 (1.0, 1.2)	0.9 (0.8, 1.0)
	Ratio of Fold-Change (mRNA-1345/Placebo) (95% CI)		6.9 (4.0, 11.8)		4.9 (4.4, 5.5)	
RSV preF IgG bAb (AU/mL)	mRNA-1345	19	13.3 (8.6, 20.7)	1544	8.1 (7.5, 8.6)	1.6 (1.1, 2.6)
	Placebo	49	1.0 (0.9, 1.0)	459	1.1 (1.0, 1.1)	0.9 (0.8, 1.0)
	Ratio of Fold-Change (mRNA-1345/Placebo) (95% CI)		13.9 (8.9, 21.6)		7.6 (7.0, 8.2)	
RSV postF IgG bAb (AU/mL)	mRNA-1345	19	7.4 (5.0, 10.9)	1544	6.1 (5.7, 6.6)	1.2 (0.8, 1.8)
	Placebo	49	1.0 (1.0, 1.0)	459	1.1 (1.0, 1.1)	0.9 (0.9, 1.0)
	Ratio of Fold-Change (mRNA-1345/Placebo) (95% CI)		7.5 (5.1, 11.2)		5.8 (5.3, 6.3)	

bAb, binding antibody; CI, confidence interval; FC, fold-change; GM, geometric mean; IgG, immunoglobulin G; LRTD, lower respiratory tract disease; nAb, neutralizing antibody; postF, postfusion; preF, prefusion; RSV, respiratory syncytial virus.

Table S14. Fold-rise antibody marker geometric means by case/non-case strata and by vaccine and placebo in the Day 29 case-cohort set for RSV-ARD.

Immunologic Marker	Treatment Group or Ratio of GM	Cases Fold-Change (95% CI)		Non-Cases Fold-Change (95% CI)		Ratio of Fold-Change (Cases/Non-Cases) (95% CI)
		n	mean (95% CI)	n	mean (95% CI)	FC (95% CI)
RSV-A nAb (IU/mL)	mRNA-1345	79	11.2 (8.8, 14.2)	1478	8.6 (7.8, 9.5)	1.3 (1.0, 1.7)
	Placebo	160	1.0 (0.9, 1.0)	328	1.0 (0.9, 1.1)	1.0 (0.9, 1.1)
	Ratio of Fold-Change (mRNA-1345/Placebo) (95% CI)		11.4 (8.9, 14.7)		8.9 (7.8, 10.2)	
RSV-B nAb (IU/mL)	mRNA-1345	79	5.7 (4.7, 7.1)	1478	5.3 (4.8, 5.8)	1.1 (0.9, 1.4)
	Placebo	160	1.0 (0.9, 1.1)	328	1.0 (0.9, 1.1)	1.0 (0.9, 1.2)
	Ratio of Fold-Change (mRNA-1345/Placebo) (95% CI)		5.7 (4.6, 7.1)		5.4 (4.8, 6.2)	
RSV preF IgG bAb (AU/mL)	mRNA-1345	79	9.3 (7.7, 11.3)	1478	8.0 (7.5, 8.7)	1.2 (0.9, 1.4)
	Placebo	160	1.0 (1.0, 1.0)	328	1.0 (1.0, 1.1)	1.0 (0.9, 1.0)
	Ratio of Fold-Change (mRNA-1345/Placebo) (95% CI)		9.5 (7.8, 11.5)		7.9 (7.3, 8.6)	
RSV postF IgG bAb (AU/mL)	mRNA-1345	79	7.1 (5.9, 8.6)	1478	6.0 (5.5, 6.5)	1.2 (1.0, 1.5)
	Placebo	160	1.0 (1.0, 1.0)	328	1.0 (1.0, 1.0)	1.0 (0.9, 1.0)
	Ratio of Fold-Change (mRNA-1345/Placebo) (95% CI)		7.2 (5.9, 8.7)		5.9 (5.4, 6.5)	

ARD, acute respiratory disease; bAb, binding antibody; CI, confidence interval; FC, fold-change; GM, geometric mean; IgG, immunoglobulin G; nAb, neutralizing antibody; postF, postfusion; preF, prefusion; RSV, respiratory syncytial virus.

Table S15. The weights assigned by the Super Learner algorithm to each individual model for predicting the occurrence of each RSV endpoint (RSV-LRTD-2+, RSV-LRTD-3+, and RSV-ARD) in placebo recipients in the per-protocol efficacy set for immune correlate analysis (PPESICA).

Endpoint	Learner	Screen	AUC	Weight
RSV-LRTD-2+	SL.gam	All	0.645	0.570
	SL.gam	screen.corP	0.609	0.167
	SL.glm	screen.corP	0.609	0.167
	SL.xgboost	All	0.601	0.096
	SL.gam	screen.glmnet	0.604	0.000
	SL.glm	All	0.624	0.000
	SL.glm	screen.glmnet	0.608	0.000
	SL.glm.interaction	screen.corP	0.589	0.000
	SL.glm.interaction	screen.glmnet	0.597	0.000
	SL.glmnet	All	0.599	0.000
	SL.mean	All	0.423	0.000
	SL.ranger	All	0.580	0.000
RSV-LRTD-3+	SL.gam	All	0.690	0.609
	SL.glm	All	0.681	0.296
	SL.ranger	All	0.615	0.091
	SL.glm	screen.glmnet	0.668	0.004
	SL.gam	screen.corP	0.641	0.000
	SL.gam	screen.glmnet	0.666	0.000
	SL.glm	screen.corP	0.641	0.000
	SL.glm.interaction	screen.corP	0.603	0.000
	SL.glm.interaction	screen.glmnet	0.624	0.000
	SL.glmnet	All	0.641	0.000
	SL.mean	All	0.368	0.000
	SL.xgboost	All	0.646	0.000
RSV-ARD	SL.gam	All	0.617	0.467
	SL.glm.interaction	screen.glmnet	0.600	0.259
	SL.gam	screen.glmnet	0.606	0.123
	SL.xgboost	All	0.562	0.078
	SL.glm.interaction	screen.corP	0.580	0.047
	SL.glm	screen.glmnet	0.600	0.026
	SL.gam	screen.corP	0.583	0.000
	SL.glm	All	0.595	0.000
	SL.glm	screen.corP	0.578	0.000
	SL.glmnet	All	0.592	0.000
	SL.mean	All	0.428	0.000
	SL.ranger	All	0.571	0.000

ARD, acute respiratory disease; AUC, area under the curve; LRTD, lower respiratory tract disease; RSV, respiratory syncytial virus.

Table S16. Predictors in learners with positive weights by the Super Learner algorithm for modeling the occurrence of RSV-LRTD-2+ in placebo recipients in the per-protocol efficacy set for immune correlate analysis (PPESICA) who had RSV-LRTD-2+ onset or censored more than 7 days after the Day 29 visit.

Learner	Screen	Weight	Predictors	Coefficients	Odds Ratio	Gain	Cover	Frequency
SL.gam	All	0.570	(Intercept)	-5.367	0.005	NA	NA	NA
SL.gam	All	0.570	s(AGE, 2)	0.009	1.009	NA	NA	NA
SL.gam	All	0.570	s(EFSBL, 2)	-0.095	0.909	NA	NA	NA
SL.gam	All	0.570	REGION1	-0.133	0.875	NA	NA	NA
SL.gam	All	0.570	SEX	-0.096	0.908	NA	NA	NA
SL.gam	All	0.570	LRTDRSKF	0.097	1.102	NA	NA	NA
SL.gam	All	0.570	HSTCOVID	0.133	1.142	NA	NA	NA
SL.gam	All	0.570	HOSPCOVD	0.023	1.023	NA	NA	NA
SL.gam	All	0.570	BMIGR1	-0.019	0.981	NA	NA	NA
SL.gam	All	0.570	PECOMINT	-0.011	0.989	NA	NA	NA
SL.gam	All	0.570	ASTHMA	0.255	1.290	NA	NA	NA
SL.gam	All	0.570	CRD	-0.782	0.457	NA	NA	NA
SL.gam	All	0.570	DIABETES	0.092	1.096	NA	NA	NA
SL.gam	All	0.570	CHF	0.038	1.039	NA	NA	NA
SL.gam	All	0.570	ALRD	0.053	1.054	NA	NA	NA
SL.gam	All	0.570	WBINCOME_UpperMiddleIncome	-0.117	0.890	NA	NA	NA
SL.gam	All	0.570	WBINCOME_HighIncome	0.094	1.099	NA	NA	NA
SL.gam	All	0.570	ETHNIC_Hispanic	-0.442	0.643	NA	NA	NA
SL.gam	All	0.570	ETHNIC_NotReported	-1.153	0.316	NA	NA	NA
SL.gam	All	0.570	ETHNIC_Unknown	-0.430	0.651	NA	NA	NA
SL.gam	screen.corP	0.167	(Intercept)	-5.194	0.006	NA	NA	NA
SL.gam	screen.corP	0.167	LRTDRSKF	0.076	1.079	NA	NA	NA
SL.gam	screen.corP	0.167	HSTCOVID	0.146	1.157	NA	NA	NA
SL.gam	screen.corP	0.167	PECOMINT	0.070	1.073	NA	NA	NA
SL.gam	screen.corP	0.167	ASTHMA	0.228	1.256	NA	NA	NA
SL.gam	screen.corP	0.167	WBINCOME_UpperMiddleIncome	0.018	1.018	NA	NA	NA
SL.gam	screen.corP	0.167	WBINCOME_HighIncome	0.138	1.148	NA	NA	NA
SL.gam	screen.corP	0.167	ETHNIC_Hispanic	-0.448	0.639	NA	NA	NA
SL.glm	screen.corP	0.167	(Intercept)	-5.194	0.006	NA	NA	NA
SL.glm	screen.corP	0.167	LRTDRSKF	0.076	1.079	NA	NA	NA
SL.glm	screen.corP	0.167	HSTCOVID	0.146	1.157	NA	NA	NA
SL.glm	screen.corP	0.167	PECOMINT	0.070	1.073	NA	NA	NA
SL.glm	screen.corP	0.167	ASTHMA	0.228	1.256	NA	NA	NA
SL.glm	screen.corP	0.167	WBINCOME_UpperMiddleIncome	0.018	1.018	NA	NA	NA
SL.glm	screen.corP	0.167	WBINCOME_HighIncome	0.138	1.148	NA	NA	NA
SL.glm	screen.corP	0.167	ETHNIC_Hispanic	-0.448	0.639	NA	NA	NA
SL.xgboost	All	0.096	AGE	NA	NA	0.361	0.358	0.417
SL.xgboost	All	0.096	EFSBL	NA	NA	0.219	0.176	0.191
SL.xgboost	All	0.096	SEX	NA	NA	0.090	0.073	0.080
SL.xgboost	All	0.096	PECOMINT	NA	NA	0.089	0.052	0.083
SL.xgboost	All	0.096	ETHNIC_Hispanic	NA	NA	0.068	0.059	0.027
SL.xgboost	All	0.096	ASTHMA	NA	NA	0.047	0.087	0.031
SL.xgboost	All	0.096	BMIGR1	NA	NA	0.033	0.037	0.044
SL.xgboost	All	0.096	DIABETES	NA	NA	0.030	0.034	0.035
SL.xgboost	All	0.096	HSTCOVID	NA	NA	0.017	0.019	0.014
SL.xgboost	All	0.096	WBINCOME_HighIncome	NA	NA	0.014	0.048	0.036
SL.xgboost	All	0.096	REGION1	NA	NA	0.013	0.020	0.017

Learner	Screen	Weight	Predictors	Coefficients	Odds Ratio	Gain	Cover	Frequency
SL.xgboost	All	0.096	WBINCOME_UpperMiddleIncome	NA	NA	0.011	0.017	0.012
SL.xgboost	All	0.096	LRTDRSKF	NA	NA	0.009	0.019	0.014

LRTD, lower respiratory tract disease; NA, not applicable; RSV, respiratory syncytial virus.

Table S17. Predictors in learners with positive weights by the Super Learner algorithm for modeling the occurrence of RSV-LRTD-3+ in placebo recipients in the per-protocol efficacy set for immune correlate analysis (PPESICA) who had RSV-LRTD-3+ onset or censored more than 7 days after the Day 29 visit.

Learner	Screen	Weight	Predictors	Coefficients	Odds Ratio	Importance
SL.gam	All	0.609	(Intercept)	-6.595	0.001	NA
SL.gam	All	0.609	s(AGE, 2)	-0.005	0.995	NA
SL.gam	All	0.609	s(EFSBL, 2)	-0.024	0.976	NA
SL.gam	All	0.609	REGION1	-0.218	0.804	NA
SL.gam	All	0.609	SEX	-0.215	0.807	NA
SL.gam	All	0.609	LRTDRSKF	0.052	1.053	NA
SL.gam	All	0.609	HSTCOVID	0.180	1.197	NA
SL.gam	All	0.609	HOSPCOVD	-0.923	0.397	NA
SL.gam	All	0.609	BMIGR1	0.157	1.170	NA
SL.gam	All	0.609	PECOMINT	0.430	1.537	NA
SL.gam	All	0.609	ASTHMA	0.180	1.197	NA
SL.gam	All	0.609	CRD	-0.812	0.444	NA
SL.gam	All	0.609	DIABETES	-0.124	0.883	NA
SL.gam	All	0.609	CHF	0.054	1.055	NA
SL.gam	All	0.609	ALRD	-1.135	0.321	NA
SL.gam	All	0.609	WBINCOME_UpperMiddleIncome	-0.182	0.834	NA
SL.gam	All	0.609	WBINCOME_HighIncome	0.187	1.206	NA
SL.gam	All	0.609	ETHNIC_Hispanic	-0.446	0.640	NA
SL.gam	All	0.609	ETHNIC_NotReported	-1.167	0.311	NA
SL.gam	All	0.609	ETHNIC_Unknown	-0.438	0.645	NA
SL.glm	All	0.296	(Intercept)	-6.704	0.001	NA
SL.glm	All	0.296	REGION1	-0.217	0.805	NA
SL.glm	All	0.296	AGE	-0.015	0.985	NA
SL.glm	All	0.296	SEX	-0.214	0.807	NA
SL.glm	All	0.296	LRTDRSKF	0.051	1.052	NA
SL.glm	All	0.296	HSTCOVID	0.177	1.194	NA
SL.glm	All	0.296	HOSPCOVD	-1.158	0.314	NA
SL.glm	All	0.296	BMIGR1	0.159	1.172	NA
SL.glm	All	0.296	EFSBL	-0.036	0.965	NA
SL.glm	All	0.296	PECOMINT	0.431	1.539	NA
SL.glm	All	0.296	ASTHMA	0.181	1.198	NA
SL.glm	All	0.296	CRD	-1.015	0.362	NA
SL.glm	All	0.296	DIABETES	-0.119	0.888	NA
SL.glm	All	0.296	CHF	0.053	1.054	NA
SL.glm	All	0.296	ALRD	-1.417	0.242	NA
SL.glm	All	0.296	WBINCOME_UpperMiddleIncome	-0.113	0.893	NA
SL.glm	All	0.296	WBINCOME_HighIncome	0.258	1.294	NA
SL.glm	All	0.296	ETHNIC_Hispanic	-0.447	0.640	NA
SL.glm	All	0.296	ETHNIC_NotReported	-1.463	0.232	NA
SL.glm	All	0.296	ETHNIC_Unknown	-0.546	0.579	NA
SL.ranger	All	0.091	AGE	NA	NA	2.930
SL.ranger	All	0.091	EFSBL	NA	NA	2.036
SL.ranger	All	0.091	SEX	NA	NA	0.601
SL.ranger	All	0.091	BMIGR1	NA	NA	0.456
SL.ranger	All	0.091	HSTCOVID	NA	NA	0.411
SL.ranger	All	0.091	ASTHMA	NA	NA	0.374
SL.ranger	All	0.091	LRTDRSKF	NA	NA	0.374
SL.ranger	All	0.091	CHF	NA	NA	0.357
SL.ranger	All	0.091	DIABETES	NA	NA	0.354
SL.ranger	All	0.091	REGION1	NA	NA	0.348

Learner	Screen	Weight	Predictors	Coefficients	Odds Ratio	Importance
SL.ranger	All	0.091	ETHNIC_Hispanic	NA	NA	0.269
SL.ranger	All	0.091	WBINCOME_HighIncome	NA	NA	0.176
SL.ranger	All	0.091	PECOMINT	NA	NA	0.122
SL.ranger	All	0.091	WBINCOME_UpperMiddleIncome	NA	NA	0.080
SL.ranger	All	0.091	ETHNIC_NotReported	NA	NA	0.015
SL.ranger	All	0.091	ALRD	NA	NA	0.015
SL.ranger	All	0.091	HOSPCOVD	NA	NA	0.008
SL.ranger	All	0.091	CRD	NA	NA	0.005
SL.ranger	All	0.091	ETHNIC_Unknown	NA	NA	0.005
SL.glm	screen.glmnet	0.004	(Intercept)	-6.704	0.001	NA
SL.glm	screen.glmnet	0.004	REGION1	-0.217	0.805	NA
SL.glm	screen.glmnet	0.004	SEX	-0.214	0.807	NA
SL.glm	screen.glmnet	0.004	LRTDRSKF	0.051	1.052	NA
SL.glm	screen.glmnet	0.004	HSTCOVID	0.177	1.194	NA
SL.glm	screen.glmnet	0.004	HOSPCOVD	-1.158	0.314	NA
SL.glm	screen.glmnet	0.004	BMIGR1	0.160	1.174	NA
SL.glm	screen.glmnet	0.004	EFSBL	-0.038	0.963	NA
SL.glm	screen.glmnet	0.004	PECOMINT	0.431	1.539	NA
SL.glm	screen.glmnet	0.004	ASTHMA	0.181	1.198	NA
SL.glm	screen.glmnet	0.004	CRD	-1.015	0.362	NA
SL.glm	screen.glmnet	0.004	DIABETES	-0.119	0.888	NA
SL.glm	screen.glmnet	0.004	CHF	0.053	1.054	NA
SL.glm	screen.glmnet	0.004	ALRD	-1.417	0.242	NA
SL.glm	screen.glmnet	0.004	WBINCOME_UpperMiddleIncome	-0.107	0.899	NA
SL.glm	screen.glmnet	0.004	WBINCOME_HighIncome	0.264	1.302	NA
SL.glm	screen.glmnet	0.004	ETHNIC_Hispanic	-0.447	0.640	NA
SL.glm	screen.glmnet	0.004	ETHNIC_NotReported	-1.463	0.232	NA
SL.glm	screen.glmnet	0.004	ETHNIC_Unknown	-0.546	0.579	NA

LRTD, lower respiratory tract disease; NA, not applicable; RSV, respiratory syncytial virus.

Table S18. Predictors in learners with positive weights by the Super Learner algorithm for modeling the occurrence of RSV-ARD in placebo recipients in the per-protocol efficacy set for immune correlate analysis who had RSV-ARD onset or censored more than 7 days after the Day 29 visit.

Learner	Screen	Weight	Predictors	Coefficients	Odds Ratio	Gain	Cover	Frequency
SL.gam	All	0.467	(Intercept)	-4.942	0.007	NA	NA	NA
SL.gam	All	0.467	s(AGE, 2)	-0.006	0.994	NA	NA	NA
SL.gam	All	0.467	s(EFSBL, 2)	-0.110	0.896	NA	NA	NA
SL.gam	All	0.467	REGION1	0.103	1.108	NA	NA	NA
SL.gam	All	0.467	SEX	-0.168	0.845	NA	NA	NA
SL.gam	All	0.467	LRTDRSKF	0.142	1.153	NA	NA	NA
SL.gam	All	0.467	HSTCOVID	0.086	1.090	NA	NA	NA
SL.gam	All	0.467	HOSPCOVD	0.064	1.066	NA	NA	NA
SL.gam	All	0.467	BMIGR1	-0.016	0.984	NA	NA	NA
SL.gam	All	0.467	PECOMINT	-0.217	0.805	NA	NA	NA
SL.gam	All	0.467	ASTHMA	0.258	1.294	NA	NA	NA
SL.gam	All	0.467	CRD	-0.791	0.453	NA	NA	NA
SL.gam	All	0.467	DIABETES	0.168	1.183	NA	NA	NA
SL.gam	All	0.467	CHF	0.014	1.014	NA	NA	NA
SL.gam	All	0.467	ALRD	0.088	1.092	NA	NA	NA
SL.gam	All	0.467	WBINCOME_UpperMiddleIncome	0.153	1.165	NA	NA	NA
SL.gam	All	0.467	WBINCOME_HighIncome	0.016	1.016	NA	NA	NA
SL.gam	All	0.467	ETHNIC_Hispanic	-0.435	0.647	NA	NA	NA
SL.gam	All	0.467	ETHNIC_NotReported	-1.180	0.307	NA	NA	NA
SL.gam	All	0.467	ETHNIC_Unknown	-0.439	0.645	NA	NA	NA
SL.glm.interaction	screen.glmnet	0.259	(Intercept)	-5.850	0.003	NA	NA	NA
SL.glm.interaction	screen.glmnet	0.259	REGION1	0.046	1.047	NA	NA	NA
SL.glm.interaction	screen.glmnet	0.259	SEX	-0.614	0.541	NA	NA	NA
SL.glm.interaction	screen.glmnet	0.259	LRTDRSKF	-1.904	0.149	NA	NA	NA
SL.glm.interaction	screen.glmnet	0.259	HSTCOVID	0.053	1.054	NA	NA	NA
SL.glm.interaction	screen.glmnet	0.259	HOSPCOVD	-0.829	0.436	NA	NA	NA
SL.glm.interaction	screen.glmnet	0.259	EFSBL	-0.099	0.906	NA	NA	NA
SL.glm.interaction	screen.glmnet	0.259	ASTHMA	0.058	1.060	NA	NA	NA
SL.glm.interaction	screen.glmnet	0.259	CRD	-0.996	0.369	NA	NA	NA
SL.glm.interaction	screen.glmnet	0.259	ALRD	-0.670	0.512	NA	NA	NA
SL.glm.interaction	screen.glmnet	0.259	ETHNIC_Hispanic	-0.506	0.603	NA	NA	NA
SL.glm.interaction	screen.glmnet	0.259	ETHNIC_NotReported	-1.453	0.234	NA	NA	NA
SL.glm.interaction	screen.glmnet	0.259	ETHNIC_Unknown	-0.541	0.582	NA	NA	NA
SL.glm.interaction	screen.glmnet	0.259	REGION1:SEX	-0.070	0.932	NA	NA	NA
SL.glm.interaction	screen.glmnet	0.259	REGION1:LRTDRSKF	-0.103	0.902	NA	NA	NA
SL.glm.interaction	screen.glmnet	0.259	REGION1:HSTCOVID	0.043	1.044	NA	NA	NA
SL.glm.interaction	screen.glmnet	0.259	REGION1:HOSPCOVD	0.056	1.058	NA	NA	NA
SL.glm.interaction	screen.glmnet	0.259	REGION1:EFSBL	0.085	1.089	NA	NA	NA
SL.glm.interaction	screen.glmnet	0.259	REGION1:ASTHMA	-0.001	0.999	NA	NA	NA
SL.glm.interaction	screen.glmnet	0.259	REGION1:CRD	0.011	1.011	NA	NA	NA
SL.glm.interaction	screen.glmnet	0.259	REGION1:ALRD	0.395	1.484	NA	NA	NA
SL.glm.interaction	screen.glmnet	0.259	REGION1:ETHNIC_Hispanic	-0.124	0.883	NA	NA	NA
SL.glm.interaction	screen.glmnet	0.259	REGION1:ETHNIC_NotReported	0.000	1.000	NA	NA	NA
SL.glm.interaction	screen.glmnet	0.259	REGION1:ETHNIC_Unknown	0.003	1.003	NA	NA	NA
SL.glm.interaction	screen.glmnet	0.259	SEX:LRTDRSKF	-2.066	0.127	NA	NA	NA
SL.glm.interaction	screen.glmnet	0.259	SEX:HSTCOVID	0.033	1.034	NA	NA	NA
SL.glm.interaction	screen.glmnet	0.259	SEX:HOSPCOVD	-0.023	0.977	NA	NA	NA
SL.glm.interaction	screen.glmnet	0.259	SEX:EFSBL	-0.128	0.880	NA	NA	NA
SL.glm.interaction	screen.glmnet	0.259	SEX:ASTHMA	0.186	1.204	NA	NA	NA

Learner	Screen	Weight	Predictors	Coefficients	Odds Ratio	Gain	Cover	Frequency
SL.glm.interaction	screen.glmnet	0.259	SEX:CRD	0.033	1.034	NA	NA	NA
SL.glm.interaction	screen.glmnet	0.259	SEX:ALRD	-0.046	0.955	NA	NA	NA
SL.glm.interaction	screen.glmnet	0.259	SEX:ETHNIC_Hispanic	0.106	1.112	NA	NA	NA
SL.glm.interaction	screen.glmnet	0.259	SEX:ETHNIC_NotReported	0.029	1.029	NA	NA	NA
SL.glm.interaction	screen.glmnet	0.259	SEX:ETHNIC_Unknown	0.005	1.005	NA	NA	NA
SL.glm.interaction	screen.glmnet	0.259	LRTDRSKF:HSTCOVID	-0.004	0.996	NA	NA	NA
SL.glm.interaction	screen.glmnet	0.259	LRTDRSKF:HOSPCOVD	-0.034	0.967	NA	NA	NA
SL.glm.interaction	screen.glmnet	0.259	LRTDRSKF:EFSBL	-0.012	0.988	NA	NA	NA
SL.glm.interaction	screen.glmnet	0.259	LRTDRSKF:ASTHMA	0.058	1.060	NA	NA	NA
SL.glm.interaction	screen.glmnet	0.259	LRTDRSKF:CRD	0.009	1.009	NA	NA	NA
SL.glm.interaction	screen.glmnet	0.259	LRTDRSKF:ALRD	-0.390	0.677	NA	NA	NA
SL.glm.interaction	screen.glmnet	0.259	LRTDRSKF:ETHNIC_Hispanic	-0.118	0.889	NA	NA	NA
SL.glm.interaction	screen.glmnet	0.259	LRTDRSKF:ETHNIC_NotReported	-0.003	0.997	NA	NA	NA
SL.glm.interaction	screen.glmnet	0.259	LRTDRSKF:ETHNIC_Unknown	-0.011	0.989	NA	NA	NA
SL.glm.interaction	screen.glmnet	0.259	HSTCOVID:EFSBL	-0.132	0.876	NA	NA	NA
SL.glm.interaction	screen.glmnet	0.259	HSTCOVID:ASTHMA	-0.014	0.986	NA	NA	NA
SL.glm.interaction	screen.glmnet	0.259	HSTCOVID:CRD	0.003	1.003	NA	NA	NA
SL.glm.interaction	screen.glmnet	0.259	HSTCOVID:ALRD	0.034	1.035	NA	NA	NA
SL.glm.interaction	screen.glmnet	0.259	HSTCOVID:ETHNIC_Hispanic	0.062	1.064	NA	NA	NA
SL.glm.interaction	screen.glmnet	0.259	HSTCOVID:ETHNIC_NotReported	-0.007	0.993	NA	NA	NA
SL.glm.interaction	screen.glmnet	0.259	HSTCOVID:ETHNIC_Unknown	-0.002	0.998	NA	NA	NA
SL.glm.interaction	screen.glmnet	0.259	HOSPCOVD:EFSBL	-0.054	0.947	NA	NA	NA
SL.glm.interaction	screen.glmnet	0.259	HOSPCOVD:ASTHMA	0.096	1.101	NA	NA	NA
SL.glm.interaction	screen.glmnet	0.259	HOSPCOVD:CRD	0.007	1.007	NA	NA	NA
SL.glm.interaction	screen.glmnet	0.259	HOSPCOVD:ALRD	0.041	1.042	NA	NA	NA
SL.glm.interaction	screen.glmnet	0.259	HOSPCOVD:ETHNIC_Hispanic	0.706	2.026	NA	NA	NA
SL.glm.interaction	screen.glmnet	0.259	HOSPCOVD:ETHNIC_NotReported	0.132	1.141	NA	NA	NA
SL.glm.interaction	screen.glmnet	0.259	EFSBL:ASTHMA	0.063	1.065	NA	NA	NA
SL.glm.interaction	screen.glmnet	0.259	EFSBL:CRD	0.004	1.004	NA	NA	NA
SL.glm.interaction	screen.glmnet	0.259	EFSBL:ALRD	0.023	1.023	NA	NA	NA
SL.glm.interaction	screen.glmnet	0.259	EFSBL:ETHNIC_Hispanic	0.334	1.397	NA	NA	NA
SL.glm.interaction	screen.glmnet	0.259	EFSBL:ETHNIC_NotReported	0.024	1.024	NA	NA	NA
SL.glm.interaction	screen.glmnet	0.259	EFSBL:ETHNIC_Unknown	0.013	1.013	NA	NA	NA
SL.glm.interaction	screen.glmnet	0.259	ASTHMA:CRD	-0.006	0.994	NA	NA	NA
SL.glm.interaction	screen.glmnet	0.259	ASTHMA:ALRD	0.024	1.024	NA	NA	NA
SL.glm.interaction	screen.glmnet	0.259	ASTHMA:ETHNIC_Hispanic	-0.227	0.797	NA	NA	NA
SL.glm.interaction	screen.glmnet	0.259	ASTHMA:ETHNIC_NotReported	-0.019	0.981	NA	NA	NA
SL.glm.interaction	screen.glmnet	0.259	ASTHMA:ETHNIC_Unknown	0.002	1.002	NA	NA	NA
SL.glm.interaction	screen.glmnet	0.259	CRD:ALRD	-0.005	0.995	NA	NA	NA
SL.glm.interaction	screen.glmnet	0.259	CRD:ETHNIC_Hispanic	0.020	1.020	NA	NA	NA
SL.glm.interaction	screen.glmnet	0.259	ALRD:ETHNIC_Hispanic	-0.686	0.504	NA	NA	NA
SL.glm.interaction	screen.glmnet	0.259	ALRD:ETHNIC_NotReported	-0.007	0.993	NA	NA	NA
SL.gam	screen.glmnet	0.123	(Intercept)	-4.948	0.007	NA	NA	NA
SL.gam	screen.glmnet	0.123	s(EFSBL, 2)	-0.089	0.915	NA	NA	NA
SL.gam	screen.glmnet	0.123	REGION1	0.048	1.049	NA	NA	NA
SL.gam	screen.glmnet	0.123	SEX	-0.165	0.848	NA	NA	NA
SL.gam	screen.glmnet	0.123	LRTDRSKF	0.074	1.077	NA	NA	NA
SL.gam	screen.glmnet	0.123	HSTCOVID	0.089	1.093	NA	NA	NA
SL.gam	screen.glmnet	0.123	HOSPCOVD	0.064	1.066	NA	NA	NA
SL.gam	screen.glmnet	0.123	ASTHMA	0.171	1.186	NA	NA	NA
SL.gam	screen.glmnet	0.123	CRD	-0.803	0.448	NA	NA	NA
SL.gam	screen.glmnet	0.123	ALRD	0.070	1.073	NA	NA	NA
SL.gam	screen.glmnet	0.123	ETHNIC_Hispanic	-0.351	0.704	NA	NA	NA
SL.gam	screen.glmnet	0.123	ETHNIC_NotReported	-1.182	0.307	NA	NA	NA
SL.gam	screen.glmnet	0.123	ETHNIC_Unknown	-0.438	0.645	NA	NA	NA

Learner	Screen	Weight	Predictors	Coefficients	Odds Ratio	Gain	Cover	Frequency
SL.xgboost	All	0.078	AGE	NA	NA	0.378	0.318	0.398
SL.xgboost	All	0.078	EFSBL	NA	NA	0.193	0.219	0.197
SL.xgboost	All	0.078	PECOMINT	NA	NA	0.070	0.047	0.053
SL.xgboost	All	0.078	SEX	NA	NA	0.070	0.063	0.081
SL.xgboost	All	0.078	ETHNIC_Hispanic	NA	NA	0.056	0.072	0.030
SL.xgboost	All	0.078	BMIGR1	NA	NA	0.050	0.046	0.047
SL.xgboost	All	0.078	ASTHMA	NA	NA	0.036	0.058	0.032
SL.xgboost	All	0.078	HSTCOVID	NA	NA	0.036	0.041	0.035
SL.xgboost	All	0.078	LRTDRSKF	NA	NA	0.033	0.033	0.025
SL.xgboost	All	0.078	DIABETES	NA	NA	0.025	0.028	0.031
SL.xgboost	All	0.078	WBINCOME_HighIncome	NA	NA	0.023	0.025	0.027
SL.xgboost	All	0.078	REGION1	NA	NA	0.017	0.034	0.026
SL.xgboost	All	0.078	WBINCOME_UpperMiddleIncome	NA	NA	0.013	0.017	0.017
SL.glm.interaction	screen.corP	0.047	(Intercept)	-5.049	0.006	NA	NA	NA
SL.glm.interaction	screen.corP	0.047	SEX	-0.162	0.850	NA	NA	NA
SL.glm.interaction	screen.corP	0.047	ASTHMA	-0.072	0.931	NA	NA	NA
SL.glm.interaction	screen.corP	0.047	WBINCOME_UpperMiddleIncome	0.372	1.451	NA	NA	NA
SL.glm.interaction	screen.corP	0.047	WBINCOME_HighIncome	0.460	1.584	NA	NA	NA
SL.glm.interaction	screen.corP	0.047	ETHNIC_Hispanic	-0.476	0.621	NA	NA	NA
SL.glm.interaction	screen.corP	0.047	SEX:ASTHMA	0.109	1.115	NA	NA	NA
SL.glm.interaction	screen.corP	0.047	SEX:WBINCOME_UpperMiddleIncome	-0.021	0.979	NA	NA	NA
SL.glm.interaction	screen.corP	0.047	SEX:WBINCOME_HighIncome	-0.032	0.969	NA	NA	NA
SL.glm.interaction	screen.corP	0.047	SEX:ETHNIC_Hispanic	0.050	1.051	NA	NA	NA
SL.glm.interaction	screen.corP	0.047	ASTHMA:WBINCOME_UpperMiddleIncome	1.567	4.792	NA	NA	NA
SL.glm.interaction	screen.corP	0.047	ASTHMA:WBINCOME_HighIncome	1.601	4.958	NA	NA	NA
SL.glm.interaction	screen.corP	0.047	ASTHMA:ETHNIC_Hispanic	-0.169	0.845	NA	NA	NA
SL.glm.interaction	screen.corP	0.047	WBINCOME_UpperMiddleIncome:ETHNIC_Hispanic	0.278	1.320	NA	NA	NA
SL.glm	screen.glmnet	0.026	(Intercept)	-4.988	0.007	NA	NA	NA
SL.glm	screen.glmnet	0.026	REGION1	0.050	1.051	NA	NA	NA
SL.glm	screen.glmnet	0.026	SEX	-0.167	0.846	NA	NA	NA
SL.glm	screen.glmnet	0.026	LRTDRSKF	0.074	1.077	NA	NA	NA
SL.glm	screen.glmnet	0.026	HSTCOVID	0.089	1.093	NA	NA	NA
SL.glm	screen.glmnet	0.026	HOSPCOVD	0.063	1.065	NA	NA	NA
SL.glm	screen.glmnet	0.026	EFSBL	-0.106	0.899	NA	NA	NA
SL.glm	screen.glmnet	0.026	ASTHMA	0.171	1.186	NA	NA	NA
SL.glm	screen.glmnet	0.026	CRD	-0.936	0.392	NA	NA	NA
SL.glm	screen.glmnet	0.026	ALRD	0.070	1.073	NA	NA	NA
SL.glm	screen.glmnet	0.026	ETHNIC_Hispanic	-0.352	0.703	NA	NA	NA
SL.glm	screen.glmnet	0.026	ETHNIC_NotReported	-1.379	0.252	NA	NA	NA
SL.glm	screen.glmnet	0.026	ETHNIC_Unknown	-0.510	0.600	NA	NA	NA

ARD, acute respiratory disease; NA, not applicable; RSV, respiratory syncytial virus.

DocuSign Envelope ID: E2A985B4-0319-4230-B508-9A9089A8A58A

ModernaTX, Inc.

Statistical Analysis Plan for Assessing the Immune Correlates of Protection in the Phase 2/3 Randomized, Observe-Blind, Placebo-Controlled Study of the mRNA-1345, an mRNA Vaccine Targeting Respiratory Syncytial Virus (RSV), in Adults ≥ 60 Years of Age

Author(s): Chong Ma, Lingyi Zheng, Jiejun Du, Lan Lan

Document type: Statistical Analysis Plan

Document version: Version 1.0

Document date: 01-September-2023

CONFIDENTIAL – DO NOT DISTRIBUTE OUTSIDE OF MODERNATX, INC. WITHOUT WRITTEN PERMISSION.

TABLE OF CONTENTS

Contents

Abbreviations 3

1. Introduction 5

2. Study Cohorts and Endpoints 5

2.1 Study Cohort for Immune Correlates Analyses 5

2.2 Immunogenicity SubCohort 6

2.3 Study endpoints 7

3. Immune Correlate Analysis Objectives 8

4. Methodology 9

4.1 Descriptive and graphical summary of Day 1, Day 29, and Day 181 antibody marker levels 10

4.2 Correlates of Risk Analyses 11

4.3 Correlates of Protection Analyses 12

5. References 12

APPROVAL 14

Abbreviations

Abbreviation	Definition
AE	adverse event
AESI	adverse event of special interest
ANCOVA	analysis of covariance
AR	adverse reaction
ARD	acute respiratory disease
ATC	Anatomical Therapeutic Chemical
bAb	binding antibodies
BMI	body mass index
ccIAS	case-cohort Immunogenicity Analysis Set
CHF	congestive heart failure
CI	confidence interval
COPD	chronic obstructive pulmonary disease
COVID-19	Coronavirus Disease 2019
CRF	case report form
CSP	clinical study protocol
DBP	data blinding plan
DSMB	data safety monitoring board
eCRF	electronic case report form
eDiary	electronic diary
EFS	Edmonton Frailty Scale
FAS	full analysis set
GLSM	geometric least squares mean
GM	geometric mean
GMC	geometric mean concentration
GMFR	geometric mean fold-rise
GMR	geometric mean titer ratio
GMT	geometric mean titer
IA	interim analysis
IcEv	intercurrent event
IM	intramuscular
IP	investigational product
IRT	Interactive Response Technology
LLOQ	lower limit of quantification
LRTD	lower respiratory tract disease
MAAE	medically-attended adverse event
MedDRA	Medical Dictionary for Regulatory Activities
mITT	Modified intent-to-treat
MMRM	mixed model for repeated measures
MTP	multiple testing procedure

CONFIDENTIAL – DO NOT DISTRIBUTE OUTSIDE OF MODERNATX, INC. WITHOUT WRITTEN PERMISSION.

nAb	neutralizing antibodies
NP	nasopharyngeal
PPE	per-protocol efficacy
PPI	per-protocol immunogenicity
PRO	Patient-Reported Outcome
PT	preferred term
QoL	quality of life
RCDF	reverse cumulative distribution function
RSV	Respiratory Syncytial Virus
RT-PCR	reverse transcription polymerase chain reaction
SAE	serious adverse event
SAP	statistical analysis plan
SAS	Statistical Analysis System
SD	standard deviation
SMQ	Standardised MedDRA Queries
SOC	system organ class
SoEs	Schedules of Events
SRR	seroresponse rate
TEAE	treatment-emergent adverse event
TFLs	Tables, Figures and Listings
ULOQ	upper limit of quantification
VE	vaccine efficacy
WHO-DD	World Health Organization Drug Dictionary
WPAI	Work Productivity and Activity Impairment Questionnaire

CONFIDENTIAL – DO NOT DISTRIBUTE OUTSIDE OF MODERNATX, INC. WITHOUT WRITTEN PERMISSION.

1. Introduction

This SAP describes the statistical analysis of antibody biomarkers measured at Day 1, Day 29, and Day 181 as immune correlates of risk and as immune correlates of protection against the RSV primary and secondary endpoints in the Phase 2/3 Randomized, Observe-Blind, Placebo-Controlled Study of the mRNA-1345 vaccine targeting Respiratory Syncytial Virus (RSV), in adults ≥ 60 years of age.

Study mRNA-1345-P301 is a Phase 2/3, randomized, observer-blind, placebo-controlled, case-driven study to evaluate the safety and efficacy of mRNA-1345 vaccine as compared with placebo targeting Respiratory Syncytial Virus (RSV) in adults ≥ 60 years of age as compared with placebo. The immunogenicity objectives in this study include evaluating the antibody response to a single dose of mRNA-1345 vaccine from baseline to 24 months post-injection (secondary objective) and evaluating immune response biomarkers after dosing with mRNA-1345 vaccine as potential correlates of protection or risk of RSV disease (exploratory objective).

In this trial, the estimated vaccine efficacy in the primary analysis against RSV-LRTD as defined by two or more symptoms was 83.7% (95% confidence interval: 66.1 to 92.2%) and the estimated vaccine efficacy against RSV-LRTD as defined by three or more symptoms was 82.4% (95% confidence interval: 34.8 to 95.3%). The immunogenicity assessments include the following:

- Viroclinics microneutralization [MN] assay: Live virus neutralization Ab level (ND50) to RSV-A and RSV-B in IU/mL
- PPD VAC69 assay: Serum binding Ab level to RSV in AU/mL

Section 2 describes the study cohort and endpoints for the immune correlate analysis, and especially emphasizes the immunogenicity subcohort for measuring the antibody marker data at Day 1, Day 29, and Day 181 based on the case-cohort sampling design. The details for the case-cohort sampling design and the immunogenicity sampling process are provided in the mRNA-1345-P301 SAP, Version 2.0 (dated 08 May 2023) and mRNA-1345-P301 Immunogenicity Subset Sampling Plan, Version 2.0 (dated 23 January 2023). Section 3 describes the immune correlate analyses objectives and section 4 outlines the principles and methodologies for implementing the descriptive and graphical summary, and the correlates of risk and correlates of protection analyses. Section 5 provides the references.

2. Study Cohorts and Endpoints

2.1 Study Cohort for Immune Correlates Analyses

The primary population for the immune correlates analysis consists of all participants in the Per-Protocol Efficacy (PPE) Set and have the baseline visit. This primary population is the study cohort for the immune correlates analyses, which refers to be the Per-Protocol Efficacy Set for Immune Correlates Analyses (PPESICA).

This study cohort (PPESICA) is used to study the serum antibody marker of interests assessed at different visits as correlates of risk and correlates of protection against the primary and secondary endpoints of interest. This study cohort could be further restricted per the antibody marker at a certain visit.

2.2 Immunogenicity SubCohort

The Immunogenicity Subcohort was planned to be obtained by the case-cohort sampling design (Prentice, 1986) according to *mRNA-1345-P301 Immunogenicity Subset Sampling Plan* in a stratified random sample of the eligible trial participants.

The participants in the Immunogenicity SubCohort are randomly selected based on three baseline characteristics from the participants in the Full Analysis Set (FAS), and the three baseline characteristics consist of Age (Age 60-74 vs Age ≥ 75), region (Northern Hemisphere vs Southern Hemisphere), and LRTD risk status (LRTD risk present vs LRTD risk absent), respectively. In total, the eligible participants are selected at random from seven strata as follows by combining each category of the three baseline characteristics, except that the participants with Age ≥ 75 and LRTD risk present are merged by regions. The Southern Hemisphere includes Argentina, Australia, Chile, New Zealand, and South Africa, and the Northern Hemisphere includes Bangladesh, Belgium, Canada, Colombia, Costa Rica, Finland, Germany, Mexico, Japan, Panama, Poland, Singapore, S. Korea, Spain, Taiwan, UK, USA (including Puerto Rico).

Table 1 describes the planned number of participants to be sampled from each of the 7 strata, constituting a total of approximately 2,128 participants, adjusting for approximately 10% of participants who may be excluded from the Per-protocol immunogenicity subset, may not have results due to issues with serum samples or assay processing, etc. with a target of approximately 1,920 participants in the per-protocol immunogenicity subset. For the mRNA-1345 μg group, if the total eligible subjects <444 in S3 stratum or <222 in other strata, select all subjects within each stratum; for the placebo group, if the total eligible subjects <88 in S3 stratum or <44 in other strata, select all subjects within each stratum

- o S1 = Age 60-74, LRTD risk present, Northern Hemisphere;
- o S2 = Age 60-74, LRTD risk present, Southern Hemisphere;
- o S3 = Age ≥75, LRTD risk present (merged with regions);
- o S4 = Age 60-74, LRTD risk absent, Northern Hemisphere;
- o S5= Age 60-74, LRTD risk absent, Southern Hemisphere;
- o S6 = Age ≥75, LRTD risk absent, Northern Hemisphere;
- o S7 = Age ≥75, LRTD risk absent, Southern Hemisphere.

In addition, the antibody biomarkers are measured for all post-injection RSV-ARD cases with positive RT-PCR post-injection and eligible RSV-ARD symptoms that count both early cases (before 14 days after injection) and primary cases (between 14 days up to 24 months after injection).

The immune correlates analysis will be performed in the Per-Protocol Immunogenicity Case-Cohort (PPICC), which is defined as all the subjects from the Per-Protocol Efficacy Set for Immune Correlates Analysis (PPESICA) who have antibody marker data, in the Immunogenicity SubCohort or selected RSV-ARD cases for the studied endpoints, restricted to further inclusion/exclusion criteria as needed. For studying the Day 29 marker as a correlate of risk and of protection against a primary or secondary endpoint, the analysis set is Day 29 case-cohort set, which consists of the participants in the Per-Protocol Immunogenicity Case-Cohort (PPICC) who are non-cases and cases with onset date starting at least 7 days after Day 29. Similarly, for studying Day 181 markers as a correlate of risk and of protection against a primary or secondary endpoint, the analysis set is Day 181 case-cohort set, which consists of the participants in the Per-Protocol Immunogenicity Case-Cohort (PPICC) who are non-cases and cases with onset date starting at least 7 days after Day 181.

Table 1. Numbers of Participants Randomly Sampled into the Random Subcohort

	Number of participants in per-protocol immunogenicity subset (Total N=1920)						
Baseline Covariate Strata	S1	S2	S3	S4	S5	S6	S7
mRNA-1345 50 μg	200	200	400	200	200	200	200
Placebo	40	40	80	40	40	40	40
	Number of participants in Random Subcohort (Total N=2128) Adjusting for approximately 10% participants who may be excluded from the Per-protocol immunogenicity subset						
mRNA-1345 50 μg	222	222	444	222	222	222	222
Placebo	44	44	88	44	44	44	44

2.3 Study endpoints

The correlates of risk and protection related to RSV infection of the antibody markers at a certain visit (e.g., Day 29 marker) will be assessed for the following efficacy endpoints. Please refer to mRNA-1345-P301 SAP Version 2.0 for details to define and derive cases of efficacy endpoints.

- The first occurrence of RSV-LRTD with two or more symptoms within the period of 14 days post-injection up to 12 months post-injection.
- The first occurrence of RSV-LRTD with three or more symptoms within the period of 14 days post-injection up to 12 months post-injection.
- The first occurrence of RSV-ARD within the period of 14 days post-injection up to 12 months post-injection.

In addition, the following efficacy endpoints but not limited to may also be assessed.

- The first hospitalization associated with RSV-ARD or RSV-LRTD within the period of 14 days post-injection up to 12 months post-injection. If the total number of cases is <20, cases up to 24

months post-injection will be used.

- All-cause hospitalizations within the period of 14 days post-injection up to 12 months post-injection. If the total number of cases is <20, cases up to 24 months post-injection will be used.
- All-cause LRTD with two or more symptoms onwards within the period of 14 days post-injection up to 12 months post-injection .
- The first episode of RSV-LRTD with 3 or more symptoms within the period of 14 days up to 24 months post-injection.
- RSV-LRTD or RSV-ARD cases by RSV subtype A and subtype B.

The correlates of risk and protection over different time periods for selected efficacy endpoints may also be assessed.

In Study P301, efficacy was demonstrated at the first preplanned IA (30 Nov 2022) with the two primary efficacy endpoints RSV-LRTD with ≥2 symptoms, and RSV-LRTD with ≥3 symptoms meeting the pre-specified alpha-adjusted success criterion. An additional analysis of efficacy (30 Apr 2023 data cutoff date) was performed when >90% of study participants had completed at least 6 months of follow-up. At the Additional Analysis of Efficacy, there were a total of 271 cases of RSV-ARD, 174 total cases of RSV-LRTD with ≥2 symptoms and 70 cases of RSV-LRTD with ≥3 symptoms. All the 271 RSV-ARD cases were expected to have antibody marker data.

Besides the RSV-ARD cases that met protocol-defined efficacy endpoint of the first occurrence of RSV-ARD within the period of 14 days post-injection up to 12 months post-injection, additional 24 RSV-ARD cases were also expected to have antibody marker data. These cases include early RSV-ARD cases occurred before Day 14, RSV-ARD cases occurred after Month 12 and up to Month 24, and RSV-ARD cases based on the alternative definition (i.e., regardless of results from a certified lab or not).

The first correlate analysis will be performed using the data based on the 30 Apr 2023 data cutoff date. Additional correlate analysis will be performed after Day 181 immunogenicity data are available, and further correlate analysis may be performed.

3. Immune Correlate Analysis Objectives

The primary objective in this immune correlate analysis is to study the immune response biomarkers after dosing with mRNA-1345 vaccine as potential correlates of protection (CoP) and correlates of risk (CoR) of RSV disease.

1. To assess Day 29 nAb as a correlate of risk (CoR) against RSV
2. To assess Day 29 nAb as a correlate of protection (CoP) against RSV
3. To assess fold-rise in RSV nAb from Day 1 to Day 29 as a CoR against RSV
4. To assess fold-rise in RSV nAb from Day 1 to Day 29 as a CoP against RSV

5. To assess Day 1 nAb as a CoR against RSV
6. To assess whether the RSV nAb decays from Day 29 to Day 181
7. To assess whether Day 29 nAb as a CoR is modified by Day 1 antibody values

Note that the immunogenicity serum antibody assessments are conducted on Day 1, Day 15 (for phase 2), Day 29, Day 181, Day 365, Day 546, and Day 730, respectively. In this immune correlate analysis plan, the Day 29 and Day 181 antibody markers are of the primary interests for correlates of risk and correlates of protection analyses. The longitudinal analysis of the antibody marker levels at all visits is also of interests to study the duration of vaccine efficacy, and such analysis will be conducted as needed.

The CoP analyses aim to demonstrate the vaccination against different endpoints of interests by studying the causality relationship between the antibody markers and such endpoints by comparing vaccine to placebo recipients. It can directly explain how an antibody marker can predict the vaccine efficacy, which could be used as a surrogate endpoint for accelerated approval and provide fundamental evidence for the further development of the RSV vaccine.

And the CoR analyses aim to establish the credible associations of antibody markers with future risk of the endpoints of interests in the vaccinated participants in this immunogenicity case-cohort. It can provide the straightforward interpretation of how much and how long the vaccine can prevent the occurrence of different endpoints of interest. The CoP and CoR analyses complement each other, and both can demonstrate the vaccine efficacy from different perspectives.

Note that the CoR analysis for Day 1 nAb will be implemented in the placebo arm.

4. Methodology

This section describes the methodology principles to conduct the CoR and CoP analyses, and the four antibody markers, RSV-A and RSV-B neutralization antibodies and RSV Pre-F and Post-F binding antibodies, on Day 1, Day 29, and Day 181 are of the primary interests for the vaccine efficacy analyses. Note that each of the four antibody markers will be analyzed individually as a CoR or CoP against the study endpoints of interest, and the RSV-A and RSV-B neutralization antibodies will be prioritized in this immune correlate analysis. To simplify the notation, Day 29 antibody marker is used as a template antibody marker to describe the following analyses, and such analyses will be repeated for Day 181 antibody marker. And the primary endpoint of the first occurrence of RSV-LRTD with at least two symptoms is used to describe the following immune correlates analyses. Other endpoints of interests can follow the similar approach for immune correlates analyses.

In principle all CoR and CoP modeling will be adjusted by the impactful baseline risk covariates, and such potential baseline risk covariates include but not limited to:

- Risk factors for LRTD: CHF/COPD absent vs. present
- Sex: Male, Female
- BMI group
- Age: [60 years, 75 years) vs ≥ 75 years
- Age: [60 years, 70 years), [70 years, 80 years), ≥ 80 years
- Region: Northern Hemisphere vs. Southern Hemisphere
- World bank region: North America/Europe, Central/Latin America/Africa, or Asian Pacific
- World bank country classification by income level 2022: Lower-middle-income economies, Upper-middle-income economies, or High-income countries
- Comorbidities of interest: 0 and ≥ 1 pre-existing comorbidities of interest
- Frailty status: Fit (0 - 3), Vulnerable (4 - 5) or Frail (6 or more) based on Edmonton Frail Scale total score
- History of COVID-19: yes vs. no
- Hospitalization due to COVID-19: yes vs. no
- Ethnicity: Hispanic or Latino vs. Not Hispanic or Latino

Other characteristics may also be investigated such as target medical history of COPD, asthma, any chronic respiratory disease (other than COPD or asthma), any pulmonary disease (other than COPD or asthma), diabetes, congestive heart failure, advanced liver or renal disease, and immune disease. To increase the power of the antibody marker in the CoR and CoP analyses, machine learning methods may be applied to the whole placebo participants for screening the potential significant baseline risk covariates associated with the primary and key secondary endpoints. The top-ranked significant baseline risk covariates will be adjusted in the CoR and CoP analyses.

4.1 Descriptive and graphical summary of Day 1, Day 29, and Day 181 antibody marker levels

- Demographics and clinical characteristics of participants in the Per-Protocol Immunogenicity Case-Cohort (PPICC).
- Sample sizes of vaccine recipients included in the Per-Protocol Immunogenicity Case-Cohort (PPICC) in the immune correlates analyses, by baseline sampling strata and case/non-case strata
- Correlations between Day 1 and Day 29 antibody markers in the vaccine recipients in the Per-Protocol Immunogenicity Case-Cohort (PPICC)
- Correlations between Day 1 and Day 181 antibody markers in the vaccine recipients in the Per-Protocol Immunogenicity Case-Cohort (PPICC)
- Distribution (boxplot) of RSV neutralization titers and Post-F and Pre-F IgG antibody on Day 1, Day 29, and Day 181 by case/non-case status of the primary and secondary endpoints of interest in vaccine recipients and in placebo recipients respectively in the Per-Protocol Immunogenicity Case-Cohort (PPICC)

- Scatter plot of Day 1, Day 29, and Day 181 marker data by age for vaccine recipients and placebo recipients respectively in the Per-Protocol Efficacy Immunogenicity Case-Cohort (PPICC)
- Inverse probability sampling (IPS)-weighted empirical reverse cumulative distribution function (RCD) curves for Day 29 and Day 181 marker data in the vaccine recipients in the Per-Protocol Immunogenicity Case-Cohort (PPICC)

4.2 Correlates of Risk Analyses

The CoR analyses aim to assess the Day 29 antibody marker (RSV neutralization antibody against RSV-A and RSV-B and the Post-F, Pre-F IgG antibody) individually as correlates of risk with the primary and secondary endpoints of interest by adjusting for the impactful baseline risk covariates that will be selected using the machine learning method in the whole placebo participants. The CoR analyses focus on the vaccine recipients in the Per-Protocol Immunogenicity Case-Cohort (PPICC).

For the CoR analyses, the weighted Cox proportional hazards regression is used to estimate the relative risks and absolute risks of the primary and secondary endpoints of interest across continuous marker levels and the ordered trinary or binary marker levels. The inverse probability sampling (IPS) weight is applied to account for the potential imbalance and confounding factors in the Per-Protocol Immunogenicity Case-Cohort (PPICC) compared to the whole participants in the study. The IPS weight can be estimated using the following formula:

$$\widehat{w}_i(x) = N_x/n_x$$

Where $\widehat{w}_i(x)$ is the weight for the subject i within the stratum x (16 strata defined by PPICC vaccine group case, PPICC placebo group case, PPICC vaccine group non-cases divided into the 7 demographic strata, and PPICC vaccine group non-cases divided into the 7 demographic strata), and N_x is the total size of the stratum x in the whole Per-Protocol Efficacy Set for Immune Correlates Analyses (PPESICA), and the n_x is the size of the stratum x in the Per-Protocol Immunogenicity Case-Cohort (PPICC), respectively. Under the Cox proportional regression model, for the vaccine recipients the marginalized conditional risk at the antibody marker level s from time t through the final time point t_F of follow-up adjusted by the baseline factors $risk_1(t|s) = E_X[P(T \le t|s, X, A = 1)]$ is estimated by

$$\widehat{risk}_1(t|s) = \frac{\sum_{i=1}^{n} \widehat{w}_i(x)(1 - \hat{S}_1(t|s, X_i))}{\sum_{i=1}^{n} \widehat{w}_i(x)}$$

Similarly, the marginalized conditional risk for the placebo arm $\widehat{risk}_0(t_F)$ can be estimated by a Cox model marginalizing over the same baseline factors as for the analysis of the vaccine arm.

In addition, the alternative marginalized conditional risk parameter $risk_1(t|S \ge s) = E_X[P(T \le t|S \ge s, X, A = 1)]$ will be estimated by

$$\widehat{risk}_1(t|S \ge s) = \frac{\sum_{i=1}^{n} \widehat{w}_i(x)(1 - \hat{S}_1(t|S \ge s, X_i))}{\sum_{i=1}^{n} \widehat{w}_i(x)}$$

This parameter will be used to assess the vaccine efficacy of the subgroup with antibody marker levels

above a threshold.

All the parameters will be calculated with the point estimate and the 95% confidence interval using bootstrapping, and the estimated parameters will be demonstrated by appropriate plots. Multiple hypotheses testing will be adjusted using the Benjamini-Hochberg FDR approach.

4.3 Correlates of Protection Analyses

The CoP analyses aim to 1) assess the Day 29 antibody markers (RSV neutralization antibody against RSV-A and RSV-B and the Post-F, Pre-F IgG antibody) individually as correlates of vaccine efficacy (VE) in vaccine recipients; 2) assess the controlled vaccine efficacy for Day 29 antibody markers by comparing vaccine to placebo. The CoP analyses will also adjust the baseline risk factors as in the CoR analyses.

The controlled vaccine efficacy curve $CVE(s)$ will be studied and the definition is

$$CVE(s) = 1 - \frac{P(Y(1,s) = 1)}{P(Y(0) = 1)}$$

$CVE(s)$ represents the relative decrease in the risk of the occurrence of an endpoint of interest obtained by taking vaccine compared to taking placebo at a certain Day 29 antibody marker level. In the formula of $CVE(s)$, $P(Y(1,s) = 1)$ can be estimated by the marginalized conditional risk $\widehat{risk}_1(t_F|s)$ for the vaccine recipients, and $P(Y(0) = 1)$ can be estimated by the marginalized conditional risk $\widehat{risk}_0(t_F)$, respectively. Note that these two parameters are the same as the ones calculated in the CoR analyses.

Besides using the time-to-event endpoints in the CoR and CoP analyses, it is of interests to study the vaccination against the corresponding binary endpoints as CoR and CoP respectively. For the binary endpoints, the logistic regression model is used to assess the vaccine efficacy of antibody biomarker data by adjusting the baseline risk covariates. The ROC plot is used to assess the predictive performance of such fitted logistic regression model and the point estimate and 95% confidence interval will be displayed.

Note that the methodology principles described above are very concise, and other methods could be applied based on the suggestions from the subject matter experts.

5. References

Gilbert, Peter B., David C. Montefiori, Adrian B. McDermott, Youyi Fong, David Benkeser, Weiping Deng, Honghong Zhou et al. "Immune correlates analysis of the mRNA-1273 COVID-19 vaccine efficacy clinical trial." Science 375, no. 6576 (2022): 43-50.

Ge, Yuancai, Hang Chu, Jiyi Chen, Peiyuan Zhuang, Qianyi Feng, William R. Smith, Pei Dong,

DocuSign Envelope ID: E2A985B4-0319-4230-B508-9A9089A8A58A

Mingxin Ye, and Jianfeng Shen. "Ultrathin MoS2 nanosheets decorated hollow CoP heterostructures for enhanced hydrogen evolution reaction." ACS Sustainable Chemistry & Engineering 7, no. 11 (2019): 10105-10111.

Fintzi, Jonathan, and Dean Follmann. "Assessing vaccine durability in randomized trials following placebo crossover." Statistics in Medicine 40, no. 27 (2021): 5983-6007.

Zou, Guangyong. "A modified poisson regression approach to prospective studies with binary data." American journal of epidemiology 159, no. 7 (2004): 702-706.

Lumley, Thomas, and Alastair Scott. "Fitting regression models to survey data." Statistical Science (2017): 265-278.

Diaz-Quijano, Fredi A. "A simple method for estimating relative risk using logistic regression." BMC medical research methodology 12 (2012): 1-6.

Robins, James M., Miguel Angel Hernan, and Babette Brumback. "Marginal structural models and causal inference in epidemiology." Epidemiology (2000): 550-560.

Zhuang, Yingying, Ying Huang, and Peter B. Gilbert. "Simultaneous inference of treatment effect modification by intermediate response endpoint principal strata with application to vaccine trials." The international journal of biostatistics 16, no. 1 (2019): 20180058.

Huang, Ying, Peter B. Gilbert, and Julian Wolfson. "Design and estimation for evaluating principal surrogate markers in vaccine trials." Biometrics 69, no. 2 (2013): 301-309.

Follmann, Dean. "Augmented designs to assess immune response in vaccine trials." Biometrics 62, no. 4 (2006): 1161-1169.

Gilbert, Peter B., and Michael G. Hudgens. "Evaluating candidate principal surrogate endpoints." Biometrics 64, no. 4 (2008): 1146-1154.

Vakulenko-Lagun, Bella, Micha Mandel, and Rebecca A. Betensky. "Inverse probability weighting methods for cox regression with right-truncated data." Biometrics 76, no. 2 (2020): 484-495.

Prentice, R. (1986), "case-cohort design for epidemiologic cohort studies and disease prevention trials." Biometrika, 73, 1-11.

DocuSign Envelope ID: E2A985B4-0319-4230-B508-9A9089A8A58A

APPROVAL

Protocol Number:	mRNA-1345-P301
Document Description:	Statistical Analysis Plan for Assessing the Immune Correlates of Protection
Version Number:	1.0
Effective Date:	01 September 2023

Chong Ma, Senior Manager, Biostatistics

DocuSigned by:
Chong Ma
42C0292B91C447C...

01 September 2023

Signature Date

Jiejun Du, Director, Biostatistics

DocuSigned by:
Jiejun Du
4560328E3C0C420...

Signature Date

Lan Lan, Director, Biostatistics

DocuSigned by:
Lan Lan
875BCED8851E41E...

01 September 2023

Signature Date

Lingyi Zheng, Senior Director, Biostatistics

DocuSigned by:
Lingyi Zheng
F50F92813A5D4D8...

01 September 2023

Signature Date

Eleanor Wilson, Senior Director, Clinical Development

DocuSigned by:
Eleanor Wilson
EE210569A412436...

01 September 2023

Signature Date

ModernaTX, Inc.

mRNA-1345-P301

A PHASE 2/3, RANDOMIZED, OBSERVER-BLIND, PLACEBO-CONTROLLED STUDY TO EVALUATE THE SAFETY AND EFFICACY OF mRNA-1345, AN mRNA VACCINE TARGETING RESPIRATORY SYNCYTIAL VIRUS (RSV), IN ADULTS ≥ 60 YEARS OF AGE

Immunogenicity Subset Sampling Plan

Version 2.0
Version Date: 23 January, 2023

Prepared by:

PPD
3575 Quakerbridge Road, Suite 201
Hamilton, New Jersey (NJ), 08619
USA

Table of Contents

1 Document History **3**

2 Overview **4**

3 Data Cutoff for Sampling of Random SubCohort **4**

4 Random SubCohort **5**

4.1 Eligibility Criteria *5*

4.2 Sample Size *5*

4.3 Sampling Method *6*

4.4 Random SubCohort List *7*

5 Post-injection RSV-ARD Cases **8**

6 Roles and Responsibilities Related to the Sampling and Maintenance of the Random SubCohort and Post-injection RSV-ARD Cases **9**

7 APPROVAL **10**

1 Document History

Version	Date	Changes
V1.0	27 September, 2022	Original Version (Version 1.0)
V2.0	23 January, 2023	1. Clarified the immunogenicity objectives and ccIAS cohort definition in Section 1 Overview.<br>2. Added Section 3 data cutoff date of sampling<br>3. Updated the stratum definition in Section 4.1<br>4. Removed Section 6 ccIAS cohort list.<br>5. Updated Section 7.

2 Overview

There are two immunogenicity objectives in this study: to evaluate the antibody response to a single dose of mRNA-1345 vaccine from baseline to 24 months post-injection (secondary objective) and to evaluate immune response biomarkers after dosing with mRNA-1345 vaccine as potential correlates of protection or risk of RSV disease (exploratory objective). Antibody marker data will be assessed in a subset of trial participants selected using a case-cohort sampling design (Prentice (1986)). This participant subset is referred as the case-cohort Immunogenicity Analysis Set (ccIAS). The ccIAS consists of a random stratified sample of all trial participants (Random SubCohort), augmented with all trial participants with any post-injection RSV-ARD cases defined as eligible RSV-ARD symptoms and positive RT-PCR post injection, i.e.,

ccIAS cohort = Random SubCohort + Post-injection RSV-ARD cases.

This document describes the sampling process of the ccIAS cohort selection:

- Random SubCohort: PPD unblinded biostatistician will provide the rationale of selecting Random SubCohort for assessment of immunogenicity and details of how the Random SubCohort will be drawn.
- Post-injection RSV-ARD cases: PPD unblinded biostatistician will provide the list of post-injection RSV-ARD cases in blinded and unblinded fashion to selected study team personnel. The list of post-injection RSV-ARD cases may be provided as ongoing basis as determined by the study team.

After the completion of ccIAS cohort selection, the immunogenicity samples of the ccIAS cohort will be processed and the immunogenicity data will be available for the ccIAS cohort. For country specific submission, additional immunogenicity samples may be tested.

3 Data Cutoff for Sampling of Random SubCohort

The sampling process will be based on the same data cut used for the first interim analysis (IA1). A total of ~35538 participants (~96% of the planned sample size of 37000), which is adequately representative of the trial population, will be included for sampling. This approach preserves the consistency of participant population between the IA1 and the immunogenicity analyses, it also provides necessary time for the sponsor to process the immunogenicity samples without delaying subsequent trial activities as the sampling process could start before the trail reaches the full enrollment.

The data cutoff date and algorithm will be the same as the IA1. Specifically, the rules for generating the post-cut subject list for sampling are described below. More details can be found in the document of *mRNA-1345-P301 IA1 Data Cutoff–Definition, Algorithm, and Application*.

- If the participant has a randomization date, then only participants with randomization date on or prior to 31-OCT-2022 will be kept in the post-cut participant list.
- If the participant has no randomization date (i.e., were not randomized), then only participants with inform consent date on or prior to 31-OCT-2022 will be kept the post-cut participant list.

4 Random SubCohort

4.1 Eligibility Criteria

Within the post-cut participant list from Section 3, only participants in the FAS (ADSL.FASFL= 'Y') will be eligible for sampling into the Random SubCohort.

4.2 Sample Size

Participants are randomly sampled into the Random Subcohort within each stratum. The strata are defined based on the randomization strata and geographic region as follows:

- S1 = Age 60-74, LRTD risk present, Northern Hemisphere;
- S2 = Age 60-74, LRTD risk present, Southern Hemisphere;
- S3 = Age ≥75, LRTD risk present;
- S4 = Age 60-74, LRTD risk absent, Northern Hemisphere;
- S5= Age 60-74, LRTD risk absent, Southern Hemisphere;
- S6 = Age ≥75, LRTD risk absent, Northern Hemisphere;
- S7 = Age ≥75, LRTD risk absent, Southern Hemisphere.

Note that for S3, southern and northern hemisphere are combined to allow adequate participants for sampling.

Southern hemisphere includes Argentina, Australia, Chile, New Zealand, and South Africa; northern hemisphere includes: Bangladesh, Belgium, Canada, Colombia, Costa Rica, Finland, Germany, Mexico, Japan, Panama, Poland, Singapore, S. Korea, Spain, Taiwan, UK, USA (including Puerto Rico).

Table 1 describes the number of participants sampled from each of the 7 strata, constituting a total of approximately 2,128 participants, adjusting for approximately 10% of participants who may be excluded from the Per-protocol immunogenicity subset, may not have results due to issues with serum samples or assay processing, etc. with a target of approximately 1,920 participants in the per-protocol immunogenicity subset.

This sampling design enables characterization of immune response (bAb and nAb readouts) in all relevant subgroups. The sampling is implemented without replacement.

Table 1. Numbers of Participants Randomly Sampled into the Random Subcohort

	Number of participants in per-protocol immunogenicity subset (Total N=1920)						
Baseline Covariate Strata	S1	S2	S3	S4	S5	S6	S7
mRNA-1345 50 µg	200	200	400	200	200	200	200
Placebo	40	40	80	40	40	40	40
	Number of participants in Random Subcohort (Total N=2128)<br>Adjusting for approximately 10% participants who may be excluded from the Per-protocol immunogenicity subset						
mRNA-1345 50 µg	222	222	444	222	222	222	222
Placebo	44	44	88	44	44	44	44

4.3 Sampling Method

Step 1. Subset the eligible participants: Keep subjects (USUBJID) if FAS flag (ADSL.FASFL) ='Y'.

Step 2. Stratified random sampling algorithm

1) Assign subjects to following strata:
   - Set to S1 if ADSL.STRATFCT= '60 TO 74 YEARS AND LRTD RISK PRESENT' and ADSL.REGION1 ='Northern Hemisphere',
   - Set to S2 if ADSL.STRATFCT= '60 TO 74 YEARS AND LRTD RISK PRESENT' and ADSL.REGION1 ='Southern Hemisphere',
   - Set to S3 if ADSL.STRATFCT = '>= 75 YEARS AND LRTD RISK PRESENT'
   - Set to S4 if ADSL.STRATFCT = '60 TO 74 YEARS AND LRTD RISK ABSENT' and ADSL.REGION1 ='Northern Hemisphere',
   - Set to S5 if ADSL.STRATFCT = '60 TO 74 YEARS AND LRTD RISK ABSENT' and ADSL.REGION1 ='Southern Hemisphere',
   - Set to S6 if ADSL.STRATFCT = '>= 75 YEARS AND LRTD RISK ABSENT' and ADSL.REGION1 ='Northern Hemisphere',
   - Set to S7 if ADSL.STRATFCT = '>= 75 YEARS AND LRTD RISK ABSENT' and ADSL.REGION1 ='Southern Hemisphere',
2) For each of mRNA-1345 50 µg and placebo vaccination group, randomly draw number of subjects (ADSL.USUBJID) from each stratum (also see Table 1).

a) mRNA-1345 µg group: Randomly select 444 subjects from S3 stratum, and 222 subjects from each S1, S2, S4, S5, S6 and S7 stratum where vaccination group (ADSL.TRT01P) = ‘mRNA-1345 ug’ (rounding up).

   Random sampling will be performed in each stratum separately as follows:

   Step A: In each stratum, sort the data in ascending order by ADSL.USUBJID, assign a uniform pseudo-random number (SAS RAND('UNIFORM') call) to each eligible subject, using a seed of 145923. We refer to this uniform pseudo-random number as RANDOM.

   Step B: Sort the data in ascending order by the pseudo-random number (RANDOM) assigned in Step A.

   Step C: Keep (i.e., select) the first 444 subjects in S3 stratum and keep the first 222 subjects in other strata.

   If total eligible subjects <444 in S3 stratum or <222 in other strata, select all subjects within each stratum.

b) Placebo group: Randomly select 88 subjects from S3 stratum, and 44 subjects from each S1, S2, S4, S5, S6 and S7 stratum where vaccination group (ADSL.TRT01P) = ‘Placebo’ (rounding up). Randomization method described in a) will be used, except selecting first 88 subjects or 44 subjects in Step C. If total eligible subjects <88 or <44 in some strata, select all subjects within each stratum.

3) Concatenate the subsets selected in 2). Keep subject ID (USUBJID, SUBJID), stratification for randomization (ADSL.STRATFCT), Age (ADSL.AGE), Region (ADSL.REGION1), FAS flag (ADSL.FASFL), vaccination group (ADSL.TRT01P and ADSL.TRT01A), IP injection date (ADSL.TRTSDT), Data Extraction Date (ADSL.EXSTDT) and random number (RANDOM).

4.4 Random SubCohort List

Following the process described in Section 4.3, the Random SubCohort list will be produced as follows:

- Step 1. Create a list including unique subjects in the list of Random SubCohort. Set Random SubCohort flag RANDSFL=’Y’.
- Step 2. Create two versions of Random SubCohort list as follows:
  1) One version with all unblinded data: keep all variables in the list created in Step 1.
  2) One version without unblinded data: Drop vaccination group (ADSL.TRT01P and ADSL.TRT01A), random number (RANDOM), Random SubCohort flag (RANDSFL) from the list created in Step 1 to keep the list in blinded fashion.

5 Post-injection RSV-ARD Cases

The post-injection RSV-ARD cases will include subjects with eligible RSV-ARD symptoms and positive RT-PCR post injection (refer to Table 4 in Section 6.3.2.1 of the SAP). All the post-injection RSV-ARD cases including early cases that start within 14 days from injection will be counted. Both RSV-ARD cases based on primary and alternative definition will be counted.

Post-injection RSV-ARD cases are participants with at least one record where ADTTE.FASFL ='Y' and RDV-ARD case based on primary definition (ADTTE.PARAMCD='TARD24M'and ADTTE.EVNTDESC='Event between 14 Days up to 24 Months After Injection' or 'Early Event before 14 Days After Injection') or RDV-ARD case based on alternative definition (ADTTE.PARAMCD='TARD24MX' and ADTTE.EVNTDESC='Event between 14 Days up to 24 Months After Injection' or 'Early Event before 14 Days After Injection')

Keep subject ID (USUBJID, SUBJID), stratification for randomization (ADSL.STRATFCT), Age (ADSL.AGE), Region (ADSL.REGION1), FAS flag (ADSL.FASFL), vaccination group (ADSL.TRT01P and ADSL.TRT01A) and IP injection date (ADSL.TRTSDT), Data Extraction Date (ADSL.EXSTDT).

The list of post-injection RSV-ARD will be produced as follows:

- Step 1. If the subject with ADSL.RSIFL ='Y', then Set Random SubCohort flag RANDSFL='Y'. For RDV-ARD case based on primary definition, set ARDPRMFL="Y"; for RSV-ARD case based on alternative definition, set ARDALTFL="Y".
- Step 2. Set NEWCASFL ="Y" if the subject ID was not in the previous list; else set NEWCASFL ="N".
- Step 3. Create two versions of post-injection RSV-ARD case list as follows:
    1) One version with all unblinded data: keep all variables in the list created in Step 1.
    2) One version without unblinded data: Drop vaccination group (ADSL.TRT01P and ADSL.TRT01A) from the list created in Step 1 to keep the list in blinded fashion.

6 Roles and Responsibilities Related to the Sampling and Maintenance of the Random SubCohort and Post-injection RSV-ARD Cases

Table 2 describes roles and responsibilities related to the sampling and maintenance of the Random SubCohort and post-injection RSV-ARD cases .

Table 2. Roles and Responsibilities Related to the Sampling and Maintenance of the Random SubCohort and post-injection RSV-ARD cases

Role	Responsibilities
PPD Unblinded Biostatistician	- To extract stratified random sample of study participants (i.e., Random SubCohort)<br>- To provide the Random SubCohort list and the list of post-injection RSV-ARD cases in blinded and unblinded fashion to selected study team personnel
Study Biomarker Representative	- To receive the list of participants in the Random SubCohort list in blinded fashion and the list of post-injection RSV-ARD cases. The list of post-injection RSV-ARD cases may be provided as ongoing basis as determined by the study team.<br>- To facilitate with vendors to have immunogenicity samples of participants in the Random SubCohort and post-injection RSV-ARD cases processed

7 APPROVAL

Protocol Number: mRNA-1345-P301 Amendment 2

Document Description: Immunogenicity Subset Sampling Plan (Version 2.0)

Version Date: 23 Janurary 2023

Author(s):

Xiaoning Li, PhD, Biostatistician II, Biostatistics, PPD, part of Thermo Fisher Scientific

DocuSigned by:
Xiaoning Li

23-Jan-2023

Signer Name: Xiaoning Li
Signing Reason: I approve this document
Signing Time: 23-Jan-2023 | 6:22:10 PM GMT
749175B9F53F47779A657CFCC4A02BEB

Signature Date

Approved by:

Grace Chen, MD, Sr. Director, Clinical Development, Moderna

DocuSigned by:
Grace Chen

24-Jan-2023

Signer Name: Grace Chen
Signing Reason: I approve this document
Signing Time: 24-Jan-2023 | 10:54:42 PM EST
4515C7E88160498680567D6B50A32E5D

Signature Date

Archana Kapoor, PhD, Associate Director, Clinical Biomarkers, Moderna

DocuSigned by:
Archana Kapoor

23-Jan-2023

Signer Name: Archana Kapoor
Signing Reason: I have reviewed this document
Signing Time: 23-Jan-2023 | 2:28:42 PM EST
CE60E6159CAD49228C147DD7CC7F1C76

Signature Date

Jiejun Du, PhD, Director, Biostatistics, Moderna

DocuSigned by:
Jiejun Du

23-Jan-2023

Signer Name: Jiejun Du
Signing Reason: I approve this document
Signing Time: 23-Jan-2023 | 11:05:25 PM GMT
4560328E3C0C4209A8BA666BB9D1B0FC

Signature Date

DocuSign

Certificate Of Completion

Envelope Id: BEE27C16244A4C1BB549C6BD8B39593A		Status: Completed

Subject: Complete with DocuSign: mRNA-1345-P301_Immunogenicity_Sampling_Plan_V2_Clean_20230123.docx

Source Envelope:

Document Pages: 10	Signatures: 4	Envelope Originator:
Certificate Pages: 4	Initials: 0	Xiaoning Li
AutoNav: Enabled		929 N Front St
EnvelopeId Stamping: Disabled		Wilmington, NC 28401
Time Zone: (UTC) Monrovia, Reykjavik		Xiaoning.Li@ppd.com
		IP Address: 208.127.66.81

Record Tracking

Status: Original<br>23 January 2023 | 18:15	Holder: Xiaoning Li<br>Xiaoning.Li@ppd.com	Location: DocuSign

Signer Events	Signature	Timestamp
Archana Kapoor<br>Archana.Kapoor@modernatx.com<br>Associate Director<br>Moderna Inc.<br>Security Level: Email, Account Authentication (Required)	DocuSigned by:<br>Archana kapoor<br>Signer Name: Archana Kapoor<br>Signing Reason: I have reviewed this document<br>Signing Time: 23-Jan-2023 | 2:28:42 PM EST<br>CE60E6159CAD49228C147DD7CC7F1C76<br><br>Signature Adoption: Pre-selected Style<br>Signature ID:<br>CE60E615-9CAD-4922-8C14-7DD7CC7F1C76<br>Using IP Address: 185.172.189.72<br><br>With Signing Authentication via DocuSign password<br>With Signing Reasons (on each tab):<br>I have reviewed this document	Sent: 23 January 2023 | 18:21<br>Viewed: 23 January 2023 | 19:27<br>Signed: 23 January 2023 | 19:28

Electronic Record and Signature Disclosure:
Accepted: 23 January 2023 | 19:27
ID: 83898565-bcee-4077-afe0-978329d7c062

Signer Events	Signature	Timestamp
Grace Chen<br>grace.chen@modernatx.com<br>Security Level: Email, Account Authentication (Required)	Grace Chen<br><br>Signature Adoption: Pre-selected Style<br>Signature ID:<br>4515C7E8-8160-4986-8056-7D6B50A32E5D<br>Using IP Address: 185.172.189.88<br><br>With Signing Authentication via DocuSign password<br>With Signing Reasons (on each tab):<br>I approve this document	Sent: 23 January 2023 | 18:21<br>Resent: 25 January 2023 | 03:35<br>Resent: 25 January 2023 | 03:47<br>Viewed: 25 January 2023 | 03:54<br>Signed: 25 January 2023 | 03:54

Electronic Record and Signature Disclosure:
Accepted: 25 January 2023 | 03:54
ID: 360d8b44-39b7-4809-aa4f-ffd76cc831e9

Signer Events	Signature	Timestamp
Jiejun Du<br>jdu@modernatx.com<br>Security Level: Email, Account Authentication (Required), Login with SSO	DocuSigned by:<br>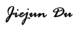<br>Signer Name: Jiejun Du<br>Signing Reason: I approve this document<br>Signing Time: 23-Jan-2023 | 11:05:25 PM GMT<br>4560328E3C0C4209A8BA666BB9D1B0FC<br><br>Signature Adoption: Pre-selected Style<br>Signature ID:<br>4560328E-3C0C-4209-A8BA-666BB9D1B0FC<br>Using IP Address: 185.172.189.88<br><br>With Signing Authentication via DocuSign password<br>With Signing Reasons (on each tab):<br>I approve this document	Sent: 23 January 2023 | 18:21<br>Resent: 23 January 2023 | 23:03<br>Viewed: 23 January 2023 | 23:05<br>Signed: 23 January 2023 | 23:05

Electronic Record and Signature Disclosure:
Accepted: 23 January 2023 | 23:05
ID: 6bd2e686-555b-407e-8428-ad9192e92ec5

Xiaoning Li<br>xiaoning.li@ppd.com<br>PPD 21 CFR Part 11<br>Security Level: Email, Account Authentication (Required)	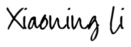<br><br>Signature Adoption: Pre-selected Style<br>Signature ID:<br>749175B9-F53F-4777-9A65-7CFCC4A02BEB<br>Using IP Address: 208.127.66.81<br><br>With Signing Authentication via DocuSign password<br>With Signing Reasons (on each tab):<br>I approve this document	Sent: 23 January 2023 | 18:21<br>Viewed: 23 January 2023 | 18:22<br>Signed: 23 January 2023 | 18:22

Electronic Record and Signature Disclosure:
Not Offered via DocuSign

In Person Signer Events	Signature	Timestamp

Editor Delivery Events	Status	Timestamp

Agent Delivery Events	Status	Timestamp

Intermediary Delivery Events	Status	Timestamp

Certified Delivery Events	Status	Timestamp

Carbon Copy Events	Status	Timestamp

Witness Events	Signature	Timestamp

Notary Events	Signature	Timestamp

Envelope Summary Events	Status	Timestamps
Envelope Sent	Hashed/Encrypted	23 January 2023 | 18:21
Envelope Updated	Security Checked	23 January 2023 | 23:03
Envelope Updated	Security Checked	25 January 2023 | 03:35
Envelope Updated	Security Checked	25 January 2023 | 03:47
Certified Delivered	Security Checked	23 January 2023 | 18:22
Signing Complete	Security Checked	23 January 2023 | 18:22
Completed	Security Checked	25 January 2023 | 03:54

Payment Events	Status	Timestamps

Electronic Record and Signature Disclosure

PPD has established a corporate policy regarding the appropriate use of electronic records and electronic signatures, POL-00392, Appropriate Use of Electronic Records and Electronic Signatures
