# Supplementary material for: Immune correlates analysis of mRNA-1345 RSV vaccine efficacy clinical trial
Source: Nat Commun. 2025 Jul 3;16:6118. doi: 10.1038/s41467-025-61153-x (PMC12229610; doi:10.1038/s41467-025-61153-x)
Supplement: Supplementary file 3 — Description of Additional Supplementary Files [file 41467_2025_61153_MOESM3_ESM.pdf]

Description of Additional Supplementary Files for

Immune Correlates Analysis of mRNA-1345 RSV Vaccine Efficacy Clinical Trial

Chong Ma et al

Corresponding author: Lingyi Zheng, lingyi.zheng@modernatx.com

Filename: Supplementary Code 1

Description: Supplementary Code 1 includes four key components – common folder, code folder, renv.lock file, and README.md file – which together enable the reproducibility of all analyses presented in the article using R version 4.2.2.
