# Supplementary material for: Immune correlates analysis of mRNA-1345 RSV vaccine efficacy clinical trial
Source: Nat Commun. 2025 Jul 3;16:6118. doi: 10.1038/s41467-025-61153-x (PMC12229610; doi:10.1038/s41467-025-61153-x)
Supplement: Supplementary file 5 — Reporting Summary [file 41467_2025_61153_MOESM5_ESM.pdf]

nature portfolio

Corresponding author(s): Lingyi Zheng

Last updated by author(s): May 20, 2025

Reporting Summary

Nature Portfolio wishes to improve the reproducibility of the work that we publish. This form provides structure for consistency and transparency in reporting. For further information on Nature Portfolio policies, see our Editorial Policies and the Editorial Policy Checklist.

Statistics

For all statistical analyses, confirm that the following items are present in the figure legend, table legend, main text, or Methods section.

n/a	Confirmed	
☐	☒	The exact sample size (*n*) for each experimental group/condition, given as a discrete number and unit of measurement
☐	☒	A statement on whether measurements were taken from distinct samples or whether the same sample was measured repeatedly
☐	☒	The statistical test(s) used AND whether they are one- or two-sided<br>*Only common tests should be described solely by name; describe more complex techniques in the Methods section.*
☐	☒	A description of all covariates tested
☐	☒	A description of any assumptions or corrections, such as tests of normality and adjustment for multiple comparisons
☐	☒	A full description of the statistical parameters including central tendency (e.g. means) or other basic estimates (e.g. regression coefficient) AND variation (e.g. standard deviation) or associated estimates of uncertainty (e.g. confidence intervals)
☐	☒	For null hypothesis testing, the test statistic (e.g. *F*, *t*, *r*) with confidence intervals, effect sizes, degrees of freedom and *P* value noted<br>*Give P values as exact values whenever suitable.*
☒	☐	For Bayesian analysis, information on the choice of priors and Markov chain Monte Carlo settings
☐	☒	For hierarchical and complex designs, identification of the appropriate level for tests and full reporting of outcomes
☐	☒	Estimates of effect sizes (e.g. Cohen's *d*, Pearson's *r*), indicating how they were calculated

Our web collection on statistics for biologists contains articles on many of the points above.

Software and code

Policy information about availability of computer code

Data collection	All analyses were done reproducibly based on R scripts in R version 4.2.2 that are provided with this paper.
Data analysis	All analyses were done reproducibly based on R scripts in R version 4.2.2 that are provided with this paper.

For manuscripts utilizing custom algorithms or software that are central to the research but not yet described in published literature, software must be made available to editors and reviewers. We strongly encourage code deposition in a community repository (e.g. GitHub). See the Nature Portfolio guidelines for submitting code & software for further information.

Data

Policy information about availability of data

All manuscripts must include a data availability statement. This statement should provide the following information, where applicable:

- Accession codes, unique identifiers, or web links for publicly available datasets
- A description of any restrictions on data availability
- For clinical datasets or third party data, please ensure that the statement adheres to our policy

As the trial is ongoing, access to patient-level data presented in this article and supporting clinical documents by qualified external researchers who provide methodologically sound scientific proposals may be available upon reasonable request for products or indications that have been approved by regulators in the relevant markets and subject to review from 24 months after study completion. Such requests can be made to Moderna Inc., 325 Binney Street, Cambridge, MA 02142 <<data_sharing@modernatx.com>>. A materials transfer and/or data access agreement with the sponsor will be required for accessing shared data. All other relevant data are presented in or provided with the paper. The protocol is available online: Wilson E, Goswami J, Baqui AH, et al. Efficacy and Safety of an mRNA-

Based RSV PreF Vaccine in Older Adults. N Engl J Med. 2023;389(24):2233-2244. doi:10.1056/NEJMoa2307079.

Research involving human participants, their data, or biological material

Policy information about studies with human participants or human data. See also policy information about sex, gender (identity/presentation), and sexual orientation and race, ethnicity and racism.

Reporting on sex and gender	No reporting by participant sex or gender was performed.
Reporting on race, ethnicity, or other socially relevant groupings	No reporting by participant race, ethnicity, or other socially relevant groupings was performed.
Population characteristics	Participants aged ≥60 years. Details of the inclusion/exclusion criteria were previously reported in Wilson NEJM 2023 (DOI: 10.1056/NEJMoa2307079).
Recruitment	From November 17, 2021, until December 23, 2022, 36,557 participants aged ≥60 years were randomly assigned in a 1:1 ratio to receive a single injection of mRNA-1345 50 μg or placebo at Day 1. Details of the recruitment were previously reported in Wilson NEJM 2023 (DOI: 10.1056/NEJMoa2307079).
Ethics oversight	The protocol was approved by an institutional review board (Advarra), and the trial is being conducted according to the principles of the International Council for Harmonisation Technical Requirements for Registration of Pharmaceuticals for Human Use, E6(R2) Good Clinical Practice guidelines, the Declaration of Helsinki, and all national, state, and local laws or regulations. Prior to being enrolled in the study, all participants provided written informed consent.

Note that full information on the approval of the study protocol must also be provided in the manuscript.

Field-specific reporting

Please select the one below that is the best fit for your research. If you are not sure, read the appropriate sections before making your selection.

☒ Life sciences ☐ Behavioural & social sciences ☐ Ecological, evolutionary & environmental sciences

For a reference copy of the document with all sections, see nature.com/documents/nr-reporting-summary-flat.pdf

Life sciences study design

All studies must disclose on these points even when the disclosure is negative.

Sample size	Sample size calculations have been previously published with the primary publication: DOI: 10.1056/NEJMoa2307079. This additional analysis includes data after >90% of participants (93.9% of safety set) had completed ≥6 months of study follow-up.
Data exclusions	A comprehensive data processing was conducted to guarantee the rigidity of immune correlate analysis. In the overall randomized study population (n=36557), 1394 participants were excluded per study exclusion criteria, e.g., participants did not belong to per-protocol efficacy set, did not have Day 29 visit, or Day 29 visit was out of (15, 43) days window after receiving the injection of vaccine or placebo. In the case-cohort immunogenicity analysis set (n=2130), 37 participants were excluded due to either not having efficacy data or not having both Day 1 and Day 29 antibody data. Additionally, participants who had early onset or censored of RSV endpoints within 7 days after Day 29 visit (i.e., n=34/22/48 by RSV-LRTD 2+/RSV-LRTD 3+/RSV-ARD) were excluded because of potential alteration of Day 29 antibody marker due to likely RSV infection.
Replication	mRNA-1345 P301 is a standalone phase 3 vaccine efficacy study in approximately 36,000 older adults. It demonstrated consistent reactogenicity, safety, and immunogenicity results observed in a phase 1 study (NCT04528719).
Randomization	Participants aged ≥60 years were randomly assigned in a 1:1 ratio to receive a single injection of mRNA-1345 50 μg or placebo at Day 1.
Blinding	Double-blind

Reporting for specific materials, systems and methods

We require information from authors about some types of materials, experimental systems and methods used in many studies. Here, indicate whether each material, system or method listed is relevant to your study. If you are not sure if a list item applies to your research, read the appropriate section before selecting a response.

Materials & experimental systems

n/a	Involved in the study
☐	☒ Antibodies
☒	☐ Eukaryotic cell lines
☒	☐ Palaeontology and archaeology
☒	☐ Animals and other organisms
☐	☒ Clinical data
☒	☐ Dual use research of concern
☒	☐ Plants

Methods

n/a	Involved in the study
☒	☐ ChIP-seq
☒	☐ Flow cytometry
☒	☐ MRI-based neuroimaging

Antibodies

Antibodies used

Microneutralization assay:
1. Anti-RSV antibody, obtained from MilliporeSigma, catalogue number MAB858-1, clone 133-1H.
2. Goat anti-mouse HRP obtained from Thermo Fisher Scientific, catalogue number A16072, polyclonal antibody.

Luminex® binding assay:
1. R-Phycoerythrin-conjugated AffiniPure F (Ab)2 Fragment Goat Anti-Human IgG, obtained from Jackson ImmunoResearch, catalogue number 109-116-098, polyclonal antibody.

Validation

All antibodies used in the assays were part of the assay validation. Assays were validated as per the FDA's method validation guidelines. All reagents were qualified as per the internal procedures at the testing labs.

Clinical data

Policy information about clinical studies
All manuscripts should comply with the ICMJEguidelines for publication of clinical research and a completedCONSORT checklist must be included with all submissions.

Clinical trial registration

NCT05127434

Study protocol

The full study protocol can be found as supplemental materials to the initial publication on this trial: DOI: 10.1056/NEJMoa2307079

Data collection

From November 17, 2021, until December 23, 2022, 36,557 participants aged ≥60 years from 22 countries were randomly assigned (1:1 ratio) to receive a single injection of mRNA-1345 50 µg or placebo.

Outcomes

The 2 primary efficacy endpoints were prevention of a first episode of RSV-LRTD with ≥2 or ≥3 lower respiratory symptoms between 14 days and 12 months following injection. Key secondary efficacy endpoints included prevention of a first episode of RSV-ARD with ≥1 symptom and prevention of first hospitalization associated with RSV-ARD or RSV-LRTD between 14 days and 12 months after injection. Secondary endpoints included efficacy to prevent a first episode of RSV-LRTD or RSV-ARD by RSV subtype (RSV-A and RSV-B). RSV-LRTD was defined as RSV infection confirmed by reverse transcription–polymerase chain reaction (RT-PCR) and new or worsening lower respiratory symptoms for ≥24 hours or confirmed RSV infection with radiologic evidence of pneumonia. RSV-ARD was defined as RT-PCR–confirmed RSV infection and new or worsening of ≥1 respiratory symptom for ≥24 hours. This study was designed to evaluate immune correlates as CoRs and CoPs against each RSV endpoint (RSV-LRTD-2+, RSV-LRTD-3+, and RSV-ARD) in the phase 3 trial of mRNA-1345.

Plants

Seed stocks

Report on the source of all seed stocks or other plant material used. If applicable, state the seed stock centre and catalogue number. If plant specimens were collected from the field, describe the collection location, date and sampling procedures.

Novel plant genotypes

Describe the methods by which all novel plant genotypes were produced. This includes those generated by transgenic approaches, gene editing, chemical/radiation-based mutagenesis and hybridization. For transgenic lines, describe the transformation method, the number of independent lines analyzed and the generation upon which experiments were performed. For gene-edited lines, describe the editor used, the endogenous sequence targeted for editing, the targeting guide RNA sequence (if applicable) and how the editor was applied.

Authentication

Describe any authentication procedures for each seed stock used or novel genotype generated. Describe any experiments used to assess the effect of a mutation and, where applicable, how potential secondary effects (e.g. second site T-DNA insertions, mosiacism, off-target gene editing) were examined.
